# Post-stroke Cognition is Associated with Stroke Survivor Quality of Life and Caregiver Outcomes: A Systematic Review and Meta-analysis

**DOI:** 10.1007/s11065-024-09635-5

**Published:** 2024-03-11

**Authors:** Renerus J. Stolwyk, Tijana Mihaljcic, Dana K. Wong, Diana Ramirez Hernandez, Brittany Wolff, Jeffrey M. Rogers

**Affiliations:** 1 School of Psychological Sciences and Turner Institute for Brain and Mental Health, Monash University, 18 Innovation Walk, Monash Clayton Campus, Melbourne, VIC 3800 Australia; 2 School of Psychology & Public Health, La Trobe University, Bundoora, Australia; 3 School of Psychological Science, The University of Western Australia, Perth, Australia; 4 neuroCare Group, Sydney, Australia

**Keywords:** Stroke; Quality of life, Caregiver burden, Cognition, Neuropsychological assessment

## Abstract

Disability arising from post-stroke cognitive impairment is a likely contributor to the poor quality of life (QoL) stroke survivors and their carers frequently experience, but this has not been summarily quantified. A systematic literature review and meta-analysis was completed examining the association between general and domain-specific post-stroke cognitive functioning and adult stroke survivor QoL, caregiver QoL, and caregiver burden. Five databases were systematically searched, and eligibility for inclusion, data extraction, and study quality were evaluated by two reviewers using a standardised protocol. Effects sizes (*r*) were estimated using a random effects model. Thirty-eight studies were identified, generating a sample of 7365 stroke survivors (median age 63.02 years, range 25–93) followed for 3 to 132 months post-stroke. Overall cognition (all domains combined) demonstrated a significant small to medium association with QoL, *r* = 0.23 (95% CI 0.18–0.28), *p* < 0.001. The cognitive domains of speed, attention, visuospatial, memory, and executive skills, but not language, also demonstrated a significant relationship with QoL. Regarding caregiver outcomes, 15 studies were identified resulting in a sample of 2421 caregivers (median age 58.12 years, range 18–82) followed for 3 to 84 months post-stroke. Stroke survivor overall cognitive ability again demonstrated a significant small to medium association with caregiver outcomes (QoL and burden combined), *r* = 0.17 (95% CI 0.10–0.24), *p* < 0.001. In conclusion, lower post-stroke cognitive performance is associated with significant reductions in stroke survivor QoL and poorer caregiver outcomes. Cognitive assessment is recommended early to identify those at risk and implement timely interventions to support both stroke survivors and their caregivers.

**Supplementary Information:**

The online version contains supplementary material available at 10.1007/s11065-024-09635-5.

## Introduction

Stroke is one of the leading causes worldwide of complex disability in adults (Adamson et al., 2004; Mendis, 2013; WHO, 2020), and stroke survivors frequently report lower quality of life (QoL) than age-matched controls (Aliyu et al., 2018; Goh et al., 2019). The World Health Organization (WHO) defines QoL as “individuals’ perceptions of their position in life in the context of the culture and value systems in which they live and in relation to their goals, expectations, standards and concerns”. (The WHOQOL Group, 1998). While there is no singular agreed definition of QoL, the concept is generally taken to refer to the capabilities and satisfaction individuals experience within domains such as physical, mental, and social functioning (Coons et al., 2000) and measures of QoL often include aspects of both functioning and perceptions and feelings about one’s life (Golomb et al., 2001). Not surprisingly, stroke severity and extent of disability have been shown to influence QoL (Sturm et al., 2004). Cognitive impairment is also increasingly recognised as a potential contributing factor to poor QoL (Barker-Collo et al., 2010a, b; Cumming et al., 2014; Verhoeven et al., 2011a, 2011b).

Cognitive impairment is a common consequence of stroke; two-thirds to three-quarters of stroke survivors experience cognitive difficulties depending on the timing and method of assessment (Jaillard et al., 2009; Jokinen et al., 2015; Renjen et al., 2015) and many identify support for cognition as a long-term unmet need (Andrew et al., 2014). The presence of either generalised and/or domain-specific impairment is highly predictive of chronic activity limitations and participation restrictions (Mole & Demeyere, 2020; Stolwyk et al., 2021; Watson et al., 2020). Therefore, it stands to reason that greater cognitive impairment may contribute to poorer QoL. A systematic review and meta-analysis by Watson et al. (2020) identified a significant relationship between several cognitive domains and QoL in individuals with acquired brain injuries. The review included domain-specific neuropsychological measures at variable stages of recovery. Studies using cognitive screening were not included, though these also may provide valuable information due to their widespread use. Moderator analysis to understand factors that may influence the association between stroke survivor cognition and outcome such as stroke survivor age and time post-stroke; quantifying the relationship between cognitive ability and QoL in a stroke survivor-only sample; and analysis using both screening and neuropsychological measures of cognition would be beneficial. Although cognitive screening measures are not as sensitive at detecting cognitive impairment as neuropsychological assessments (Jokinen et al., 2015), they are frequently used in research and clinical practice, and numerous international clinical guidelines for stroke care recommend that stroke survivors undergo cognitive screening followed by further neuropsychological evaluation if indicated (Canadian Stroke Best Practices, 2019; Royal College of Physicians, 2016; Stroke Foundation, 2021). Evaluation of screening measures can therefore be considered important for a more comprehensive synthesis of the research evidence, alongside evaluation of specific cognitive domains, which may have greater predictive power regarding outcomes, or may affect outcomes differently (Middleton et al., 2014; Nys et al., 2005), thereby informing biopsychosocial formulation. Another important factor to consider is the temporal relationship between cognition and outcome assessments. Concurrent assessment of stroke survivor cognition and outcome determines the association between the two variables at one point in time, while sequential assessment determines if cognitive performance earlier in stroke recovery is associated with outcome at a later time. Studies within this field vary with regards to concurrent versus sequential study designs, and delineating results between these approaches could help inform optimal timing of assessment.

Up to three-quarters of stroke survivors require assistance with their activities of daily living and receive informal care from family or friends (Dewey et al., 2002). Informal caregivers maintain a pivotal role in supporting stroke survivors in the community (Dewey et al., 2002; Di Carlo, 2009); however, this often comes at a cost (Rigby et al., 2009) referred to as caregiver burden (Montgomery et al., 1985). Caregiver burden effects 25–54% of carers (Rigby et al., 2009) and is associated with higher rates of anxiety and depression, poorer social and leisure time satisfaction, adverse effects on family relationships, increased mortality, and overall reduced quality of life (Anderson et al., 1995; Atteih et al., 2015; Haley et al., 2015; Parag et al., 2008; Schulz & Beach, 1999). Poorer cognitive functioning post-stroke may be associated with caregiver burden and QoL, but this association has limited empirical evaluation. A prior systematic literature review of caregiver burden following stroke included only five studies reporting stroke survivor cognition as a potential variable, and no clear association with caregiver burden emerged (Rigby et al., 2009). A more recent meta-analysis investigating variables that influence caregiver burden included only two studies that considered stroke survivor cognition as a variable (Zhu & Jiang, 2018), and again no conclusions could be drawn. This is potentially because the review focused on multiple predictors of caregiver burden without a specific focus on cognitive impairment. Therefore, a thorough examination of predictors of post-stroke caregiver burden with a particular emphasis on stroke survivor cognition is overdue. Again, the inclusion of both general and domain-specific cognitive assessments was considered important for a comprehensive evaluation of the literature, to firstly comment if stroke survivor cognition impacts on caregiver burden and secondly, if possible, evaluate if specific cognitive domains are associated with caregiver burden in different ways.

## Aim

Our aim was to perform a systematic literature and meta-analysis to quantitatively evaluate the relationship between post-stroke cognitive ability and stroke survivor QoL, caregiver QoL, and caregiver burden. The review focused on both general cognitive screening and neuropsychological testing of specific cognitive domains (i.e., neglect, speed, attention, visuospatial skills, language, memory, and executive function). Relationships with stroke survivor QoL and caregiver outcomes (QoL and burden) were examined at least 3 months after stroke.

## Method

### Eligibility Criteria

Original research studies published in peer review journals quantitatively investigating the relationship between cognition, measured using standardised screening tools or formal neuropsychological assessment tools, and measures of stroke survivor or caregiver QoL or caregiver burden were included. Caregiver anxiety, depression, or general wellbeing were not considered to be measures of caregiver QoL. Measures of QoL and caregiver burden were required to have been collected at least 3 months post-stroke. Cognition was required to be measured at either the same time as QoL and caregiver burden (concurrent collection) or at a prior baseline assessment (sequential collection). Additional inclusion criteria were (i) adult participants (> 18 years old); (ii) diagnosis of stroke caused by either infarction or intracerebral or subarachnoid haemorrhage; (iii) use of validated measures of cognition, QoL, and caregiver burden; and (iv) published in English. Exclusion criteria were (i) cohorts with primary diagnoses other than stroke (including vascular dementia); (ii) mixed samples including participants with traumatic brain injury, transient ischaemic attack, or dementia, with no separate reporting of stroke-only data; (iii) case series or case studies; (iv) use of only self or informant-reported measures of cognition; and (v) review studies.

### Information Sources

Ovid MEDLINE, Ovid PsycINFO, EBSCOhost CINAHL, Ovid Embase, and Elsevier Scopus were systematically searched with no restriction on the year of publication. Combinations of the following subject headings and key words were used across all databases: *stroke*, *cerebrovascular*, *CVA*, *brain vascular*, *brain ischemia*, *ischemic*, *infarct*, *occlusion*, *thrombus*, *cerebellar*, *cerebral*, *intracranial*, *MCA*, *anterior circulation*, *basal ganglia*, *posterior circulation*, *haemorrhage*, *subarachnoid haemorrhage*, *haematoma*, *bleed*, *post-stroke*, *cognition*, *neuropsychology*, *executive function*, *attention*, *concentration*, *memory*, *perceptual*, *awareness*, *insight*, *speed*, *learning*, *recall*, *self-monitoring*, *organisation*, *disorder*, *dysfunction*, *impairment*, *deficit*, *ability*, *difficulties*, *quality of life*, *activities of daily living*, *institutionalisation*, *mortality*, *caregivers*, *caregiver burden*, *function*, *activity*, *outcome*, *participation*, *independence*, *limitation*, *restriction*, *and dementia* (please see Supplementary materials for the full database search strategies). This resulted in a large volume of studies, and a separate systematic literature review and meta-analysis investigating the relationship between cognition and activity limitations and participation restrictions has been published elsewhere (Stolwyk et al., 2021). The following review focused on QoL and caregiver outcomes. The initial database search was conducted in March 2021 with an updated final search run on 29 May 2023. Reference lists from reviews and articles were also hand searched to identify other potentially relevant publications.

### Study Selection and Data Extraction

The database search results were uploaded to the Covidence systematic review software (Veritas Health Innovation), and duplicates were removed using the automated function. The eligibility assessment was performed independently by two researchers (TM and one trained research assistant BW, OZ, RK, CA, or SA) using a standardised protocol. Two researchers independently screened each title and abstract to assess the suitability for inclusion based on a checklist developed from the inclusion and exclusion criteria. Disagreements between reviewers were resolved by a third reviewer (RS). Publications considered eligible were independently reviewed in full text by two researchers (TM and either RS, JR, DW, DRH, BW, OZ). Disagreement was resolved by consensus, with a third author acting as arbitrator if an agreement could not be reached. For articles meeting the inclusion criteria, data on participants, study design, time since injury, cognition measures, outcome measures, and statistical analysis were extracted by TM, and the accuracy of the extraction and interpretation of effect directions was verified by DRH. In the updated 2023 search, the data were extracted by a research assistant (BW) and verified by TM. If the study had missing data, failed to report non-significant results, or the data were in a non-extractable format (e.g. regression weights), the corresponding author was contacted for additional information and/or clarification. If no response was received after two contact attempts either (1) the available data were extracted for inclusion in the review and the risk of reporting bias was acknowledged in the assessment of study quality; or (2) the study was excluded from the review. If the reviewers suspected that multiple articles may have been published using the same cohort, the authors were emailed for clarification. If there was no response, it was assumed that the articles represented a different cohort if sample size, follow-up period, and descriptive statistics differed.

### Data Items

#### Independent Variable Measurement

Given the lack of international consensus regarding test classifications, a pragmatic approach was taken to categorise tests into cognitive domains for the purposes of analysis (see Table 1). This allocation was based on a combination of information sources, primarily (i) test publisher technical manuals, (ii) key texts in the field of neuropsychological assessment (e.g. Lezak, 2012), (iii) review articles, and (iv) categorisations used in previous similar reviews (Watson et al., 2020). In cases of inconsistency across these sources, the authors, all of whom are registered clinical neuropsychologists, made an executive decision based on the overall weight of evidence. Some published studies presented study-defined cognitive domains based on an aggregate of multiple tests which were not consistent with the Table 1 classification and could not be separated based on the available data (e.g. Barker-Collo et al., 2010b, Chahal et al., 2011, Passier et al., 2012). In this instance the data were included in the domain reported in the published study, with consensus from current study authors that this is the main domain represented by the aggregate score, and highlighted in Table 1 and 3 by an asterisk. The only exception was the aggregate factor named ‘non-linguistic cognition’ in Dvorak et al. (2021) which comprised of tests assessing multiple aspects of cognition which could not be classified into one domain and, therefore, was only included in the overall cognition analysis. When a study reported more than one measure within a cognitive domain (e.g. multiple measures of executive function), all results were combined by the meta-analysis software into a single measure of effect.

**Table 1:** Measures of cognition by cognitive domain

Domain	Measures
Screening	Mini Mental Status Examination (MMSE) (Folstein et al., 1975); Korean- MMSE (Kang et al., 1997); Cantonese MMSE (Chiu et al., 1994) Montreal Cognitive Assessment (MoCA) (Nasreddine et al., 2005) Cambridge Cognition Examination (CAMCOG) (Roth et al., 1986) Telephone Interview for Cognitive Status (TICS) (Brandt et al., 1988) Clock drawing Community Screening Instrument for Dementia (CSI-D) (Hall et al., 2000) Frontal Assessment Battery (FAB) (Dubois et al., 2000)
Neglect	Behavioural Inattention Test (BIT)—Letter Cancellation (Wilson et al., 1987)
Visuospatial	WAIS (Wechsler, 1955) Block Design (BD) Matrix Reasoning (MR) Raven Colour Progressive Matrices (Raven & Raven, 2003) Rey–Osterrieth Complex Figure copy (Osterrieth, 1944; Rey, 1941) Repeatable Battery for the Assessment of Neuropsychological Status (RBANS) visuospatial items (Randolph et al., 1998) Judgement of Line Orientation (JLO) (Benton et al., 1994) Benton Facial Recognition Test (Benton et al., 1994) Motor Free Visual Perception Test (MFVPT) (Colarusso & Hammill, 1972)
Speed	Trail Making Test A (TMT A) (Reitan, 1955) Stroop—dots (MacLeod, 1991) Digit Symbol Coding (Coding) tasks
Attention	RBANS attention subtests (Randolph et al., 1998) Integrated Visual Auditory Continuous Performance Test (IVA-CPT) (Stanford & Turner, 2000) * Paced Auditory Serial Addition Test (PASAT) (Gronwall, 1977) WAIS (Wechsler, 1955) Digit Span Forward (DSF)
Language	Boston Naming Test (BNT) (Kaplan et al., 2001) Token Test (De Renzi & Vignolo, 1962) RBANS Language (Randolph et al., 1998) Controlled Oral Word Association Test (COWAT)—Semantic (Benton et al., 1994; Lezak, 2012) *; Category fluency tests Philadelphia Naming Test (PNT) (Roach et al., 1996) Western Aphasia Battery (WAB)—Revised (Kertesz, 2006) Boston Diagnostic Aphasia Examination (BDAE) (Goodglass et al., 2000)
Memory	Rey Auditory Verbal Learning Test (RAVLT) (Lezak, 2012; Rey, 1964; Taylor, 1959) Rey–Osterrieth Complex Figure (ROCF) (Osterrieth, 1944; Rey, 1941) Seoul Verbal Learning Test (Baek et al., 2012; Kang & Na, 2004) Word list learning test (Vilkki et al., 1998) Wechsler Memory Scale (WMS) (Wechsler, 1945)—various versions Visual Paired Associates (VPA) Doors Test (Baddleley et al., 1994) RBANS memory subtests (Randolph et al., 1998) Rivermead Behavioural Memory Test (RBMT) (Wilson et al., 1985; Wilson et al., 1989) Benton Visual Retention Test (Benton, 1945; Sivan, 1992)
Executive	TMT B; TMT AB combined score (TMT AB) (Reitan, 1955) Stroop (MacLeod, 1991) *all or colour words Delis-Kaplan Executive Function System (DKEFS)—Cognitive Fluency (Delis et al., 2001) COWAT—Phonemic Fluency (Benton et al., 1994; Lezak, 2012) * Brixton Spatial Anticipation Test (Burgess & Shallice, 1997) WAIS (Wechsler, 1955) DSB* Similarities (Si)

Attempts were made to allocate each test to the cognitive domain listed above. Some original study authors reported aggregate scores for a domain which were unable to be separated for the meta-analysis. The tests which were reported in multiple domains (due to the composite reporting) are highlighted using * in Tables 1 and 3

#### Outcome Measurement

Instruments were classified as QoL by author consensus (see Table 2) based on working definitions of the construct (The WHOQOL Group, 1998), instrument and item descriptions, and cross-checking with previous reviews (Geyh et al., 2007; von Steinbuechel et al., 2005; Watson et al., 2020). Instruments assessing caregiver burden were identified by measure and item descriptions and cross-checked in previous reviews (Rigby et al., 2009).

**Table 2:** Outcome measures

Outcome	Measure
Quality of Life	Stroke Specific QoL (SSQoL) (Williams et al., 1999) Chinese SSQoL—aneurysmal (Wong et al., 2013) Short Form—36 (SF-36)/ RAND-36 (Ware & Sherbourne, 1992) Short Form—12 (SF-12)(Ware et al., 1996; Ware et al., 2002) Life Satisfaction Questionnaire (LiSAT-9 and LiSAT-11) (Fugl-Meyer et al., 1991, 2002) Sickness Impact Profile (SIP) (Bergner et al., 1981) Stroke Adapted SIP (SA-SIP) (van Straten et al., 1997) Functional Limitations Profile (FLP) (Patrick, 2014) Stroke Impact Scale (SIS) (Duncan et al., 1999) SATIS—Stroke (Bouffioulx et al., 2008) WHOQoL-BREF (The WHOQOL Group, 1998) EuroQoL-5D (EQ-5D) + / − Visual Analogue Scale (VAS) (Devlin & Brooks, 2017; The EuroQoL Research Foundation, 1987) Nottingham Health Profile (NHP) (Hunt et al., 1985) Health-Related Quality of Life in Stroke Patients (HRQoLISP) (Owolabi & Ogunniyi, 2009)* Visual Analogue Scale (VAS) (Ahlsiö et al., 1984) Stroke and Aphasia Quality of Life Scale (SAQOL-39 g) (Hilari et al., 2009)
Caregiver Burden	Zarit Burden Interview (ZBI) (Bédard et al., 2001; Zarit et al., 1985) Bakas Caregiving Outcomes Scale (BCOS) (Bakas & Champion, 1999; Bakas et al., 2006) Caregiver Strain Index (CSI) (Robinson, 1983; Visser-Meily et al., 2004) Sense of Competence Questionnaire (SCQ) (Reimer et al., 1998) Caregiver Reaction Assessment (CRA): Negative caregiver experience subscale (Given et al., 1992; Visser-Meily et al., 2004) Perceived Burden of Caregivers of people with physical disabilities (Dumont et al., 1998) Burden Scale for Family Caregivers (Graessel et al., 2014)

^*^Use of the HRQoLISP by Akpalu et al. (2018) as a measure of QoL inferred from description in the method section. Citation by authors is to a different instrument, the SIS-16. Authors unable to be contacted for clarification

### Quality Assessment

The methodological quality and risk of bias for each study were independently assessed by two authors (TM and either RS, JR, DW, or DRH) using a modified Quality in Prognostic Studies (Hayden et al., 2006, 2014). The QUIPS is a validated assessment of the quality of prognostic studies which could be easily adapted and applied to fit the purpose of the current review to include both prognostic and non-prognostic studies. The QUIPS rates methodological quality across six domains: (1) study population, (2) study attrition (completeness of data added for non-prognostic studies), (3) prognostic factor measurement (independent variable measurement for non-prognostic studies), (4) outcome measurement, (5) confounding measurement and account, and (6) analysis. Hayden et al. (2014) provide factors to consider for assessment of potential bias under each domain which are designed to be modified based on the research question This framework was used to develop a set of criteria for the present study (see Supplementary materials). Each criterion was rated on a 3-point scale from *met*, *partly met*, or *not met/not enough evidence to determine if the criterion was met*. Each study was rated as (i) *low risk* of bias if four to six domains were rated as ‘met’, one to two as ‘partly met’, and none were rated as ‘not met’; (ii) *moderate risk* of bias if at least three domains were rated as ‘met’, two to three were rated as ‘partly met’, and a maximum of one rated as ‘not met’; and (iii) *high risk* of bias if four or more domains were ‘partly met’ or two or more domains were ‘not met’.

### Effect Measures and Synthesis Methods

Data on the association (*r*) between cognition and QoL outcomes in stroke survivors and caregiver QoL and burden were extracted in the available format (e.g. correlations and sample sizes, odds ratios, means, standard mean differences, and sample sizes, cohort 2 × 2 events) and entered into Comprehensive Meta-Analysis version 3 (Borenstein et al. 1994). A random effects mixed model was used to compute effect sizes with the magnitude of the correlation. A correlation as a measure of effect was chosen because the review explored a relationship between two continuous variables, and it was the most reported measure of effect in the studies meeting inclusion criteria. Effect sizes were categorised as follows: *small* > 0.10, *medium* > 0.30, and *large* > 0.50 (Cohen, 1988). Effect sizes were assigned a positive value if superior cognition was associated with enhanced QoL or reduced caregiver burden. While the majority of studies measured cognition as a continuous variable, five studies classified cognition as intact/impaired using a cut score (Hotter et al., 2018; Kwa et al., 1996; Patel et al., 2007; Meyer et al. (2010); Scott et al., 2008). Results from these studies were converted to correlations for the purposes of the meta-analysis.

Heterogeneity across studies was reported using Q as a total variance statistic, *I*^2^ as a relative measure of the proportion of variance (Higgins & Thompson, 2002), interpreted as low (25%), moderate (50%), or high (75%) (Higgins et al., 2003), and tau^2^ (τ^2^) as an absolute measure of the between-study variance. The risk of publication bias was assessed using visual inspection of funnel plots and Egger’s regression test (one-tailed, alpha < 0.05 suggesting publication bias).

Following a main effects analysis investigating the relationship between overall cognition and stroke survivor QoL, subgroup analyses were completed separately for each cognitive domain (visuospatial, speed, attention, language, memory, executive function, and cognitive screening). Measures of neglect and visuospatial skills were combined into visuospatial skills due to a small number of studies measuring neglect that met inclusion criteria. The majority of the studies investigating caregiver outcomes only used cognitive screening instruments, prohibiting subgroup analysis of cognitive domains.

Moderator variables were identified a priori and included factors previously identified in the research literature as having a potential impact on post-stroke cognitive outcomes. Moderator analysis of categorical variables was conducted using the Q-statistic (alpha = 0.05). Analyses included study quality (low vs moderate/high risk of bias) to explore if lower quality studies are resulting in an overestimation of the overall effect, and the time cognition was measured (concurrent versus sequential assessment). Concurrent assessment of stroke survivor cognition and outcome determines the association between the two variables at one point in time. Sequential assessment determines if cognitive performance earlier in stroke recovery is associated with outcome at a later time (i.e. can be used to predict outcome). Continuous moderator variables were examined using meta-regression and included the mean age of the stroke survivor (to comment whether age influences the relationship between cognition and QoL) and mean time since injury (to comment if the relationship between cognition and outcome changes over time). For caregiver outcomes, the mean age of the caregiver was also evaluated as a potential moderator variable.

### Reporting Bias and Certainty Assessment

The study methodology was registered with the International Prospective Register of Systematic Reviews (PROSPERO ID: CRD42018089092) in March 2018 to minimise the risk of the dissemination of research findings being influenced by the nature and direction of results. The Grading of Recommendations, Assessment, Development, and Evaluations (GRADE) framework (Atkins et al., 2004; Balshem et al., 2011; Guyatt et al., 2008) was used to rate the confidence that the estimated effect between cognition, and stroke survivor QoL and caregiver outcomes (QoL and burden) is correct. All authors were involved in rating the quality and certainty across the 5 GRADE domains (risk of bias/study limitation, imprecision, inconsistency, indirectness, and publication bias), with final ratings labelled as *very low*, *low*, *moderate*, or *high* based on consensus.

## Results

### Study Selection

**Fig. 1:**
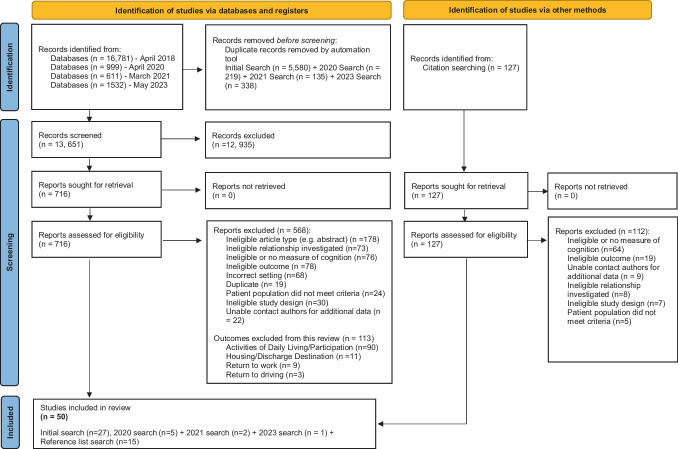
Preferred Reporting Items for Systematic Reviews and Meta-Analyses (PRISMA) Flow Diagram for Identifying Publications included in the Meta-Analytic Review

The selection process is depicted in Fig. 1 and resulted in 50 articles eligible for inclusion. Thirty-eight studies examined the relationship between cognition and QoL of stroke survivors (Table 3). Fifteen studies investigated the relationship between stroke survivor cognitive functioning and caregiver burden and/or caregiver QoL (Table 4). Three studies reported both stroke survivor and caregiver outcomes (Hotter et al., 2018; Khalid et al., 2016; Scott et al., 2008) and were included in both subgroup analyses. Eleven of the 33 authors contacted provided sufficient additional data to include the study in the current review (identifiable by notation ‘AD’ in Table 3 and 4). The studies comprised prognostic or observational cohort designs, conducted across 24 countries. The largest number of studies originated in the Netherlands (*N* = 10) followed by the USA (*N* = 5) and Hong Kong (*N* = 4). An additional 28 studies almost met the inclusion criteria. These ‘near miss’ studies, the findings, and reasons for exclusion are presented in Supplementary Table 1.

**Table 3:** Studies included in the meta-analysis—quality of life

Author (year)	Sample	Time since injury at follow up	Cognitive domain	Cognition measures	Outcome measures	Concurrent/sequential
Adamit et al. (2015) Israel	249 ischemic stroke survivors. Mage = 68.6 (9.9), range = 50–92 years, 57% males	3 months	Screening	MoCA	SIS	Concurrent
Ahmed et al. (2020) India AD	102 stroke survivors. Mage = 53.84 (14.3), 66.7% males, 94.6% ischemic	3 months	Screening	MoCA	WHOQoL-BREF	Concurrent
Akpalu (2018) Ghana	110 stroke survivors. Mage = 61 (17), 54.5% males	> 3 months	Screening	MoCA	HRQoLISP-26	Concurrent
Barker–Collo et al. (2010a) New Zealand AD	43 stroke survivors. Mage = 68.5 (15.31), 60.5% males, 2.4% haemorrhagic, 29.3% unknown stroke type. Six months follow up data available for 17–33 participants	6 months	Speed Attention executive	TMT A IVA-CPT, PASAT TMT B (MMSE < 20 excluded)	SF- 36	Concurrent
Barker–Collo et al. (2010b) New Zealand	307 stroke survivors. Mage = 72.9 (11.1), 82.4% ischemic, 4.9% undefined, 51.8% males	5 years 5.28 (0.30) years	Language Memory	COWAT*, BNT ROCF, VPA	SF- 36—Mental Component Score	Concurrent
Boosman et al. (2011) Netherlands	165 stroke survivors. Mage = 58.6 (10.6), 43% females, 67.9% ischemic	3 years	Screening	MMSE	LiSAT-9	Concurrent
Bouffioulx et al. (2011) Belgium	45 stroke survivors. Mage = 69 (10.7), 64.4% males, excluded SAH	6 months	Screening	MMSE	SATIS–Stroke	Concurrent
Bugge et al. (2001) UK AD	153 stroke survivors (ischaemic and haemorrhagic). Mage = 70.6 (35–93) years, 49% males	6 months	Screening	MMSE (at 1 month)	SF-36	Sequential
Chahal et al. (2011) New Zealand	27 patients with SAH. Mage = 62.22 (12.57); 44.4% males	5.1 (0.53) years	Speed Memory Language Visuo executive	Stoop Dots, TMT A ROCF, VPA COWAT*, BNT BD, MR IVA-CPT*, TMT B, Stroop	SF-36	Concurrent
Chang et al. (2016) Korea	2857 stroke survivors. Mage = 64.3 (12.8), 59.6% males, 80.1% ischemic, 19.9% haemorrhagic	6 months	Screening	Korean MMSE (at d/c from hospital, 18.2 days)	EuroQoL (EQ-5D)	Sequential
D’Aniello et al. (2014) Italy AD	81 stroke survivors. Mage = 62 (12.6), 59.2% males, 83.9% ischemic	1–20 years, *M* = 4 years	Screening Visuo	MMSE, Clock, FAB Ravens’ Colour Progressive Matrices	SF-36	Concurrent
Dvorak et al. (2021) USA	41 left hemisphere stroke survivors. Mage = 60.23 (10.18), 65.86% males. 90% ischemic, 10% haemorrhagic	50.64 (48.93) months, 7–255 months	Language (composite) Overall cognition (composite)	PNT Category and Letter Fluency Pseudoword repetition Reading real words Reading pseudowords WAB–Revised BDAE Spatial Span Cognitive Linguistic Quick Test (selected subtests)	SAQOL-39 g	Concurrent
Goh et al. (2019) Malaysia AD	75 stroke survivors. Mage = 66.6 (6.9), 65% males. 67 with f/u MoCA	16.6 (4.1) months	Screening	MoCA	WHOQoL-BREF	Concurrent
Hochstenbach et al. (2001) Netherlands	164 stroke survivors. Mage = 55.1 (10.9), 62% males, 86.6% ischemic	9.82 (2.05) months, 8–15 months	Screening Neglect Visuo Speed Attention Language Memory Executive	Clock Letter Cancellation BD TMT A, WAIS digit symbol DSF Animals, Sentence comprehension RAVLT, RBMT, Story Recall DSB, TMT B, Si (at 72.2 days)	SIP	Sequential
Hotter et al. (2018) Germany AD	57 ischaemic stroke survivors. Mage = 69.3 (9.8), 42% females	41 (IQR 32–48) months	Screening Language	MoCA (cut-off < 26) Token Test (cut-off > 3)	EuroQoL (EQ-5D-3L) (VAS and Index)	Concurrent
Howitt et al. (2011) Tanzania AD	52 stroke survivors. Mage = 67.1 (13.92), 30–88, 48.3% males	1–5 years post stroke (*M* = 35.6 months)	Screening	CSI-D	WHOQoL-BREF	Concurrent
Jonkman et al. (1998) Netherlands	35 first one-sided MCA ischemic stroke survivors. Mage = 55.3(3.8), 71.4% males	12 months	Decrease IQ/overall cognition	WAIS-R, WMS	SIP	Concurrent
Khalid et al. (2016) Pakistan AD	245 stroke survivors. Mage = 59(17), 68.8% males, 76.8% ischemic, 20.2% haemorrhagic	27.22 (27.76) months Range 3–172	Screening	MMSE (MMSE < 22 excluded)	SSQoL	Concurrent
Kwa et al. (1996) Netherlands	129 ischemic stroke survivors. Mage = 63.2 (14.6), 51% males, QoL data available for 97 due to communication difficulties in other 32 participants	27.6 (9.7) months	Screening	CAMCOG (impairment vs no impairment, cut-off 80)	VAS	Concurrent
Kwok et al. (2006) Hong Kong	215 stroke survivors. Mage = 70.5 (11.6), 50% males, 88.8% ischemic	12 months (3,6, and 12 months available)	Screening	Cantonese MMSE (at 3 months)	WHOQOL-BREF [HK] (12 months)	Sequential
Larson et al. (2003) USA	158 stroke survivors. Mage = 65 (31–85), 59% females. *N* = 34 f/u	6 months	Attention Language Memory Visuo	RBANS domains (at 28 days)	LiSAT-9	Sequential
Lopez-Cancio et al. (2017) Spain	206 patients with stroke of the anterior circulation. *N* = 143 f/u: medical = 72; thrombectomy = 71	12 months	Speed Executive	TMT A TMT B	EuroQoL-5D (VAS and Index)	Concurrent
McDowd et al. (2003) USA	55 ischemic stroke survivors, 56% males. 31 left hemisphere. Mage = 71.2 (6.5), 24 right hemisphere. Mage = 71.5 (6.3)	≥ 6 months	Attention	Attention tasks developed for the study (switching and divided attention). (MMSE < 18 excluded)	SIS	Concurrent
Meyer et al. (2010) Germany	113 patients with aneurysmal SAH. Mage = 54.39 (14.10), 32.7% males, 94 with MMSE	12 months	Screening	MMSE (cognitive impairment at discharge ≤ 24)	EuroQoL-5D (VAS and Index)	Sequential
Ones et al. (2005) Turkey	88 stroke survivors. Mage = 62.84 (11.42), 57% males, 83% infarction	> 6 months	Screening	MMSE	NHP	Concurrent
Park et al. (2013) Korea	50 stroke survivors with cognitive impairment but no dementia. Mage = 69 (7), 72% males	At least 90 days after stroke	Screening Speed Visuo Language Memory Executive	MMSE TMT A, Digit Symbol Coding ROCF Copy BNT, COWAT-Semantic Seoul Verbal Learning Test TMT B, COWAT—Phonemic	EuroQoL-5D	Concurrent
Passier et al. (2012) Netherlands	113 aneurysmal SAH patients. Mage = 53.6 (12.2), 83% females	12 months	Attention Visuo Memory Executive	DSF, Stroop* ROCF Copy RAVLT, ROCF, DSB*, Semantic Fluency* Brixton, Phonological Fluency (At 3 months)	SSQoL	Sequential
Patel et al. (2007) UK	397 stroke survivors. 53.4% males, 34% < 65, 33.5% 65–75, and 32.5% > 75 years	12 months	Screening	MMSE (< 24 impaired)	SF-36	Concurrent
Rohde et al. (2019) Ireland AD	101 ischemic stroke survivors. Mage = 64.3 (12.4), 67.6% males	5 years	Screening	MoCA (6 months)	SSQoL	Sequential
Safaz et al.(2016) Turkey AD	114 stroke survivors (109 at f/u). Mage = 58.2 (14.2), 68% males, 73% ischemic	> 6 months (80% of sample > 2 years post-stroke)	Screening	MMSE	SSQoL	Concurrent
Scott et al.(2008) UK	573 SAH survivors	12 months	Neuropsychological assessment	29 measures with overall categories (cog impairment vs no cog impairment)	FLP	Concurrent
Sousa et al. (2020) Portugal	30 stroke survivors. Mage = 67.13 (10.85), 47–89, 63% males, 73% ischemic	> 3 months. *M* = 54.23(59.85) months	Screening	MoCA	SF-36	Concurrent
Springer et al. (2009) USA	232 SAH survivors. Mage = 52 (13), 71% females	12 months	Screening	TICS	SIP	Concurrent
Tang et al. (2013) Hong Kong	374 stroke survivors excluded SAH separated into anxiety (mage = 64.8 (9.8), 62.8% females) and nonanxiety (mage = 66.3 (10.3), 35.1% females)	3 months	Screening	MMSE (MMSE < 20 excluded)	SSQoL	Concurrent
Verhoeven et al. (2011a) Netherlands	*N* = 111 stroke, *N* = 92 f/u, 21.6% haemorrhagic, mage = 63.7 (14.4), 49% males	12 months	Neglect Visuo Language Memory Executive	Letter Cancellation Facial Recognition, JLO Token Test; BNT RAVLT, Doors Test TMT AB	LiSat-9	Concurrent
Verhoeven et al. (2011b) Netherlands	134 stroke survivors. Mage = 56.5 (11.3), 59% males, 68.7% infarction	3 years	Screening	CAMCOG (at 45 days)	SA-SIP (3 years)	Sequential
Vilkki et al. (2012) Finland	101 SAH survivors. Mage = 48.0 (23–70) at the start and 57.7(33–80) at follow up. 49.5% males	9–13 years	Visuo Memory Executive	BD Word List Learning, Modified Benton Visual Retention Test Si, DS (at 1 year, range 9–24 months)	EuroQoL (11 years)	Sequential
Wong et al. (2013) Hong Kong	186 aneurysmal SAH. 54% completed study (*N* = 100), mage = 54(11), 31% males	12 months	Screening	MoCA	Chinese SSQoL-aneurysmal	Concurrent

Cognition: *BD* WAIS Block Design, *BNT* Boston Naming Test, *Brixton* Brixton Spatial Anticipation Test, *CAMCOG* Cambridge Cognition Examination, *COWAT* Controlled Oral Word Association Test, *CSI-D* Community Screening Instrument for Dementia, *DS* Digit Span, *DSB* Digit Span Backwards, *DSF* Digit Span Forward, *FAB* Frontal Assessment Battery, *IVA-CPT* Integrated Visual Auditory Continuous Performance Test, *JLO* Judgement of Line Orientation, *MMSE* Mini Mental Status Examination, *MoCA* Montreal Cognitive Assessment, *MR* WAIS Matrix Reasoning, *PASAT* Paced Auditory Serial Addition Test, *RAVLT* Rey Auditory Verbal Learning Test, *RBANS* Repeatable Battery for the Assessment of Neuropsychological Status, *RBMT* Rivermead Behavioural Memory Test, *ROCF* Rey–Osterrieth Complex Figure, *Si* WAIS Similarities, *TICS* Telephone Interview for Cognitive Status, *TMT* Trail Making Test, *VPA* WMS Visual Paired Associates, *WAIS* Wechsler Adult Intelligence Scale, *WMS* Wechsler Memory Scale. QoL, *FLP* Functional Limitations Profile, *HRQoLISP-26* Health-Related Quality of Life in Stroke Patients, *LiSAT* Life Satisfaction Questionnaire, *NHP* Nottingham Health Profile, *SA-SIP* Stroke Adapted Sickness Impact Profile, *SF-36* Short Form 36, *SIP* Sickness Impact Profile, *SIS* Stroke Impact Scale, *SSQoL* Stroke Specific Quality of Life, *VAS* Visual Analogue Scale, *WHOQoL–BREF* World Health Organisation Quality of Life—Abbreviated Version

^*^Aggregate scores reported for the domain and unable to separate based on available data. Included under the domain most closely represented or in overall cognition but not subdomain analysis

**Table 4:** Studies included in the meta-analysis—caregiver burden and caregiver QoL

Author (year)	Sample	Time since injury at follow up	Cognitive domain	Cognition measures	Outcome	Outcome measures	Concurrent/sequential
Abzhandaze et al. (2017) Sweden	248 ischemic stroke survivors. Mage = 64(11), 66% males, and their spouses mage = 63(11), 35% males	7 years	Screening	MMSE	Caregiver QoL	LiSAT-11	Concurrent
Caro et al. (2017) Brazil	30 stroke survivors. Mage = 70.2 (11.4), 57% ischemic, and their spouses, mage = 58.7 (13.3), 10% males	6–72 months	Screening	MMSE	Caregiver Burden Caregiver QoL	ZBI WHOQoL-BREF	Concurrent
Chen et al. (2010) Hong Kong	123 ischemic stroke survivors. Mage = 72.1 (10.0), 64.2% males, and their caregivers, mage = 61.4 (12.4), 27.6% males	27.9 (5.0) months	Screening	MMSE	Caregiver QoL	SF-36	Concurrent
Choi-Kwon et al. (2005) Korea AD	147 caregivers of stroke survivors (excluded haemorrhagic stroke). Mage = 55 (18–78), 79% females	3.4 years (1–5 years)	Screening	MMSE (23 or lower classified as cognitive dysfunction)	Caregiver Burden	SCQ	Concurrent
Green & King (2010) Canada AD	Additional data provided by authors and TIA excluded. 30 wife-caregivers (mage = 57.54, 11.61) of male stroke survivors (mage = 61.97, 11.94)	> 3 months	Screening	MMSE (MMSE < 24 excluded)	Caregiver Burden Caregiver QoL	BCOS SF-12v2	Sequential
Hotter et al. (2018) Germany AD	57 ischaemic stroke survivors. Mage = 69.3 (9.8), 42% females. No caregiver characteristics were provided	41 (32–48) months	Screening Language	MoCA (cut-off < 26) Token Test (cut-off > 3)	Caregiver Burden	Burden Scale for Family Caregivers	Concurrent
Hung et al. (2012) Taiwan	89 ischemic stroke survivors. Mage = 66.1, 54% males, and their caregivers, Mage = 52.3, 47% males, 44% were spouses, 33% sons of the patient	6 months	Screening	MMSE (≤ 23 considered poor cognition)	Caregiver Burden	CSI (CSI ≥ 7 considered high caregiver strain)	Concurrent
Khalid et al. (2016) Pakistan AD	245 stroke survivors. Mage = 59 (17), 68.8% males, 76.8% ischemic, 20.2% haemorrhagic, and their caregivers, mage = 39.18(13.4), 51.1% females, 42% children, 36% spouses	27.22 months (27.76) Range 3–172	Screening	MMSE (MMSE < 22 excluded)	Caregiver QoL	SF-36 (RAND-36)	Concurrent
Kruithof et al. (2012) Netherlands	121 stroke survivors. Mage = 54.7 (10.00), 40% females, 68.7% infraction, 21% cog impairment and their spouses, mage = 53.4 (9.5), 60% females	3 years	Screening	MMSE (cognitively impaired if ≤ 23 or unable to complete the test because of communication)	Caregiver Burden Caregiver QoL	CRA: Negative caregiver experience subscale LiSat-9	Concurrent
Kruithof et al. (2016) Netherlands	183 stroke survivors. Mage = 64.1 (11.0), 20.8% females, 95.1% infarction and their partners, mage = 62.5 (10.9), 78.7% females	12 months	Screening	MoCA (assessed 2 months post-stroke)	Caregiver Burden	CSI	Sequential
Persson et al. (2015) Sweden	248 ischemic stroke survivors. Mage = 64 (11), 34% females and their spouses, mage = 63 (11), 65% females. Cog f/u = 170	7 years	Screening	MMSE	Caregiver QoL	SF-36	Concurrent
Scott et al. (2008) UK	573 SAH survivors	12 months	Neuropsychological assessment	29 measures with overall categories (cog impairment vs no impairment)	Caregiver QoL	FLP	Concurrent
Vincent et al. (2009) Canada	158 stroke survivors recruited from acute care (*N* = 69, mage = 77.4 (7.2), 59.4% males, 91% ischemic) and inpatient rehabilitation/geriatric day hospital (*N* = 89, mage = 76.3(7.0), 48.3% males, 89.5% ischemic). 158 caregivers from acute care (*N* = 69, mage = 64.5 (11.5), 71% females, 62.3% spouses) and inpatient rehabilitation/geriatric day hospital (*N* = 87, mage = 64.4 (12.2), 70.1% females, 50.6% spouses)	6 months	Visuo Language	MFVPT Token Test (at 18–24 days)	Caregiver Burden	Perceived Burden of Caregivers of people with physical disabilities: Daily Living Skills (DLS), concern for care recipient’s well-being (CCWB), and impact on caregivers social life (ICLS). ICLS not reported	Sequential
Visser-Meily et al. (2005) Netherlands	187 stroke survivors. Mage = 56, 35% females, 74% infraction, and their spouses, mage = 54, 65% females	1 year	Screening	Cognitive impairment if MMSE ≤ 23 or if the UCO score was ≤ 3 (at 50 days)	Caregiver Burden Caregiver QoL	CSI LiSat-9	Sequential
Wu et al. (2019) USA	60 stroke survivors. Mage = 65.87 (13.23), 58.3% females, 73% ischemic, and their caregivers, mage = 59.18 (10.45), 67% females	6 months	Executive	DKEFS—Cognitive fluency	Caregiver burden	ZBI	Concurrent

Cognition: *DKEFS* Delis–Kaplan Executive Function System, *MFVPT* Motor Free Visual Perception Test, *MMSE* Mini Mental Status Examination, *MoCA* Montreal Cognitive Assessment, *UCO* Utrecht Communication Observation. QoL: *FLP* Functional Limitations Profile, *LiSAT* Life Satisfaction Questionnaire, *SF* Short Form, *WHOQoL*–*BREF* World Health Organisation Quality of Life—Abbreviated Version. Caregiver Burden: *BCOS* Bakas Caregiving Outcomes Scale, *CSI* Caregiver Strain Index, *CRA* Caregiver Reaction Assessment, *SCQ* Sense of Competence Questionnaire; *ZBI* Zarit Burden Interview

### Study Quality

Study quality was variable. Regarding stroke survivor QoL outcomes, 12 studies were rated as having an overall *low* risk of bias, 20 a *moderate* risk of bias, and six a *high* risk of bias (see Table 5). Regarding caregiver outcomes, seven studies were rated as *low*, five as *moderate*, and three as a *high* risk of bias (see Table 5). The most common risks were due to excessive study attrition/incompleteness of data, incomplete description of the study population, and lack of consideration of confounding variables. The less common risks of bias are related to inadequate measurement of prognostic factors/independent variables (cognition) and outcomes. Lower ratings in these domains were usually associated with the use of cut-off scores for continuous variables.

**Table 5:** Risk of bias ratings (Modified QUIPS)

Author (year)	Methodological quality by domain						Overall risk of bias
	1	2	3	4	5	6	
Quality of life							
Adamit et al. (2015)	Y	P	Y	Y	N	Y	Moderate
Ahmed et al. (2020)	Y	N	Y	Y	P	Y	Moderate
Akpalu et al. (2018)	Y	P	P	P	N	P	High
Barker–Collo et al. (2010a)	Y	N	Y	Y	P	Y	Moderate
Barker–Collo et al. (2010b)	Y	P	Y/P	P	Y	N	Moderate
Boosman et al. (2011)	P	N	Y	Y	P	Y	Moderate
Bouffioulx et al. (2011)	Y	P	Y	Y	Y	Y	Low
Bugge et al. (2001) AD	P	Y	Y	Y	Y	Y	Low
Chahal et al. (2011)	P	P	Y	Y	N	Y	Moderate
Chang et al. (2016)	P	N	Y	P	Y	Y	Moderate
D’Anello et al. (2014) AD	P	N	Y	Y	N	Y	High
Dvorak et al. (2021)	P	N	P	P	P	Y	Moderate
Goh et al. (2019) AD	P	P	Y	Y	Y	Y	Low
Hochstenbach et al. (2001)	Y	P	P	Y	Y	P	Moderate
Hotter et al. (2018)	P	P	P	Y	N	Y	Moderate
Howitt et al. (2011) AD	P	Y/P	Y	Y	N	Y	Moderate
Jonkman et al. (1998)	P	P	P	Y	P	P	High
Khalid et al. (2016) AD	Y	P	Y	Y	Y	Y	Low
Kwa et al. (1996)	P	N	P	P	Y	Y	Moderate
Kwok et al. (2006) AD	Y	P	Y	Y	Y	Y	Low
Larson et al. (2003)	Y/P	N	Y	Y	N	Y	High
Lopez-Cancio et al. (2017)	P	P	Y	Y	N	Y	Moderate
McDowd et al. (2003)	Y	P	P	Y	N	Y	Moderate
Meyer et al. (2010)	Y	P	P	Y	Y	Y	Low
Ones et al. (2005)	P	P	Y	Y	N	Y	Moderate
Park et al. (2013)	P	P	Y	Y	P	Y	Moderate
Passier et al. (2012)	Y	Y	P	Y	Y	Y	Low
Patel et al. (2007)	P	P	P	Y/P	Y	Y	Moderate
Rhode et al. (2019)	Y	P	Y	Y	Y	Y	Low
Safaz et al. (2016)_AD	P	P	Y	Y	Y	Y	Low
Scott et al. (2008)	P/N	P	P	Y	N	P	High
Sousa et al. (2020)	Y	P	Y	Y	N	Y	Moderate
Springer et al. (2009)	Y	P	Y	Y	N	Y	Moderate
Tang et al. (2013)	Y/P	P	Y	Y	Y	Y	Low
Verhoeven et al. (2011a)	Y	P	Y	Y	Y	Y	Low
Verhoeven et al. (2011b)	Y	P	Y	Y	Y	Y	Low
Vilkki et al. (2012)	Y	N	Y	P	P	Y	Moderate
Wong et al. (2013)	P	N	Y	Y	N	Y	High
Caregiver outcomes							
Abzhandaze et al. (2017)	Y	P	Y	Y	Y	Y	Low
Caro et al. (2017)	P	P	Y	Y	P	Y	Moderate
Chen et al. (2010)	P	P	Y	Y	Y	Y	Low
Choi-Kwon et al. (2005)	Y	P	P	Y	Y	Y	Low
Green & King (2010) AD	P	N	Y	Y	N	P	High
Hotter et al. (2018)	P/N	P	P	N	N	Y	High
Hung et al. (2012)	Y	Y	P	P	Y	Y	Low
Khalid et al. (2016) AD	P	P	Y	Y	N	Y	Moderate
Kruithof et al. (2012)	Y	N	P	Y	P	Y	Moderate
Kruithof et al. (2016)	Y	Y	Y	Y	Y	Y	Low
Persson et al. (2015)	Y	P	Y	Y	Y	Y	Low
Scott et al. (2008)	P/N	P/N	P	Y	N	P	High
Vincent et al. (2009)	Y	P	Y	Y	Y	P/N	Moderate
Visser–Meily et al. (2005)	Y	Y	P	Y	Y	Y	Low
Wu et al. (2019)	P	N	Y	Y	Y	Y	Moderate

Study quality domain 1, study population; 2, study attrition or completeness of data for non-prognostic studies; 3, prognostic factor measurement or independent variable measurement for non-prognostic studies (i.e. cognition); 4, outcome measurement; 5, confounding measurement; 6, analysis

Y criterion met, P criterion partly met, N criterion not met/not enough evidence to determine if criterion was met

### Study Characteristics

The characteristics of the studies investigating the relationship between stroke survivor cognition and their QoL and caregiver outcomes (QoL and burden) are presented in Table 6.

**Table 6:** Study characteristics

	Stroke survivor QoL studies					Caregiver outcome studies				
	*N*	%	Median	IQR	Range	*N*	%	Median	IQR	Range
Number of studies	38					15				
Sample size	7365		102	54–177	27–2271	2421		147	60–187	30–573
Stroke survivor age (years)			63.02	57–69	25–93*			64.05	60–69	26–83*
Stroke survivor gender (%male)			57	49–64	17–72			64.6	55–68	42–100
Caregiver age (years)								58.12	54–62	18–82*
Caregiver gender (male)							33		21–38	0–49
Caregiver relationship										
Spouse/any/not reported						6/6/3				
Time since injury at follow-up (months)			12	6–36	3–132			12.5	6–41	3–84
**Stroke type**										
Ischemic and haemorrhagic	16	42.1				8	53.3			
Ischemic	9	23.7				6	40.0			
Haemorrhagic	7	18.4				1	6.7			
Not reported	6	15.8								
**Cognitive assessment**										
Screening	22	57.9				11	73.3			
Neuropsychological	10	26.3				2	13.3			
Both	4	10.5				1	6.7			
Global	2	5.3				1	6.7			
**Timing of assessment**										
* Concurrent*	28	73.7				11	73.3			
Assessed (months)			12	6–36	3–63			28	12–41	6–84
* Sequential*	10	26.3				4	26.7			
Cognition assessed (days)			72	29–135	18–365			34	8–58	5–60
Outcome assessed (months)			12	6–42	6–132			9	4–12	3–12
**Caregiver outcomes**										
Burden						6	40.0			
QoL						5	33.3			
QoL and burden						4	26.7			

Cognitive Assessment—Global: test batteries with no individual cognitive domains reported

^*^Age range reported within individual studies

### Cognition and Quality of Life in Stroke Survivors

**Fig. 2:**
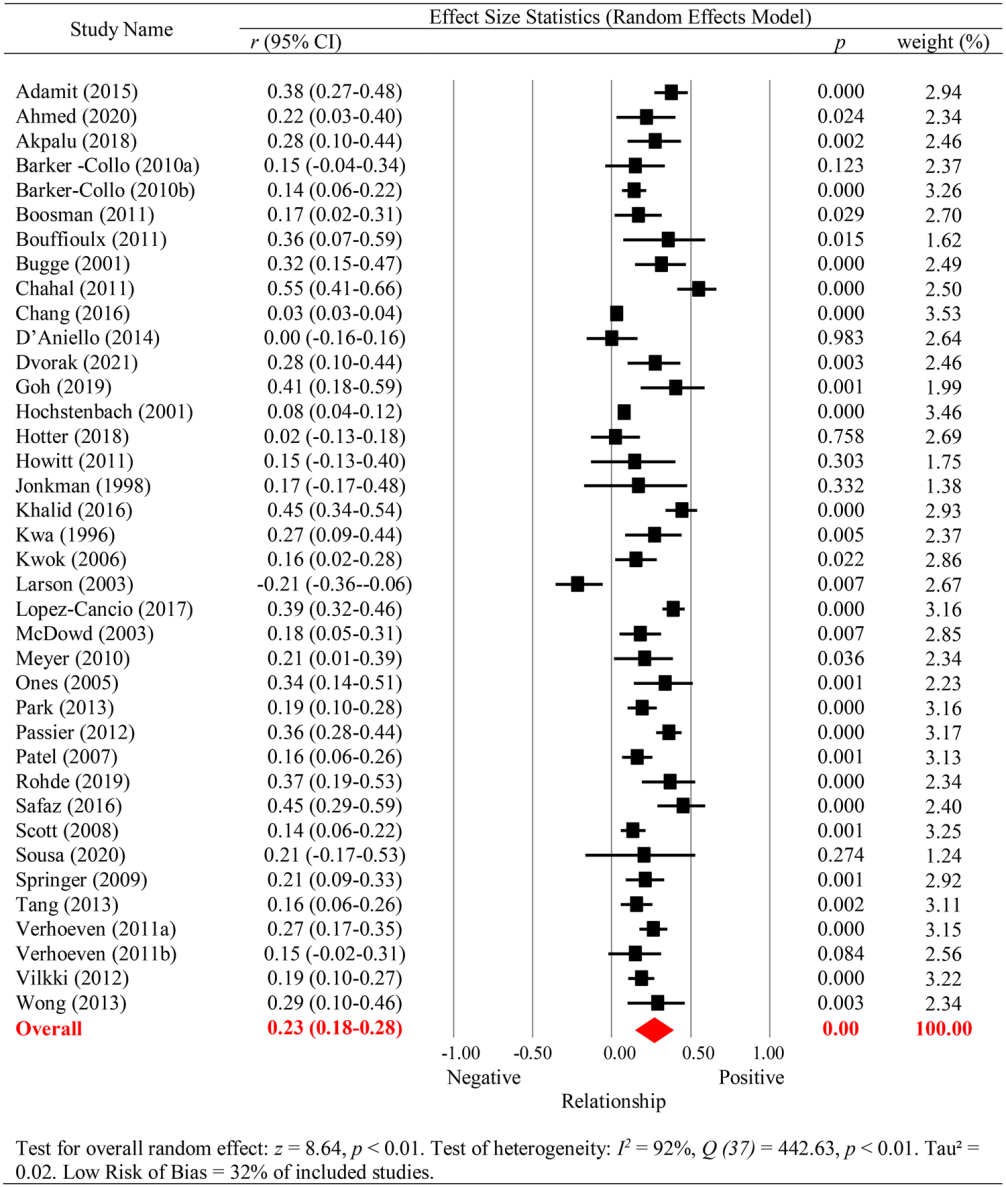
Cognition and Stroke Survivor QoL

In the 38 studies, overall reduced cognitive functioning (all neuropsychological tests and screening measures combined) had a significant *small to medium* association with diminished QoL, *r* = 0.23 (95% CI 0.18–0.28), *p* < 0.001 (see Fig. 2). Inspection of the funnel plot revealed significant asymmetry (Egger’s intercept = 2.81, *p* < 0.001, one-tailed). While there was a lack of small studies with negative findings, there appeared to be even more random variation in the large studies. This heterogeneity was explored using meta-regression where the sample size was not a significant variable (*β* =  − 0.0001, SE = 0.0001, *z* =  − 1.66, *p* = 0.10). Heterogeneity was significant, *I*^2^ = 92%, *Q* (37) = 442.63, *p* < 0.001; τ^2^ = 0.02.

**Fig. 3:**
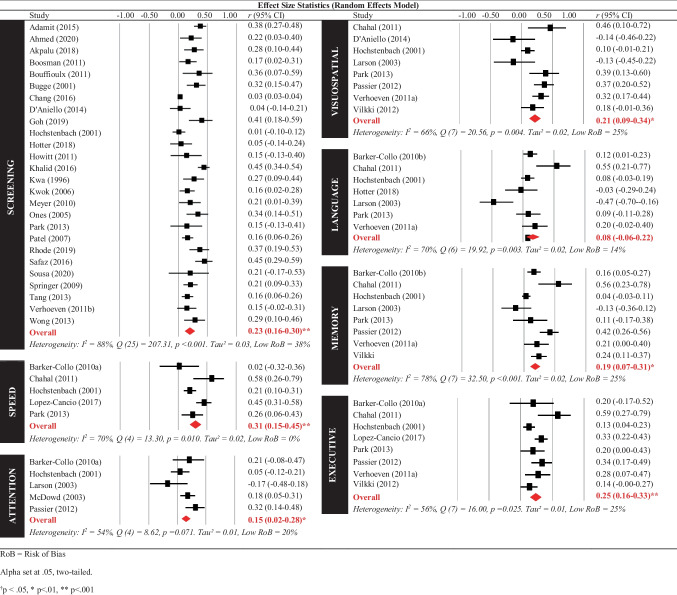
The Relationship between Cognitive Domains and Stroke Survivor QoL

**Fig. 4:**
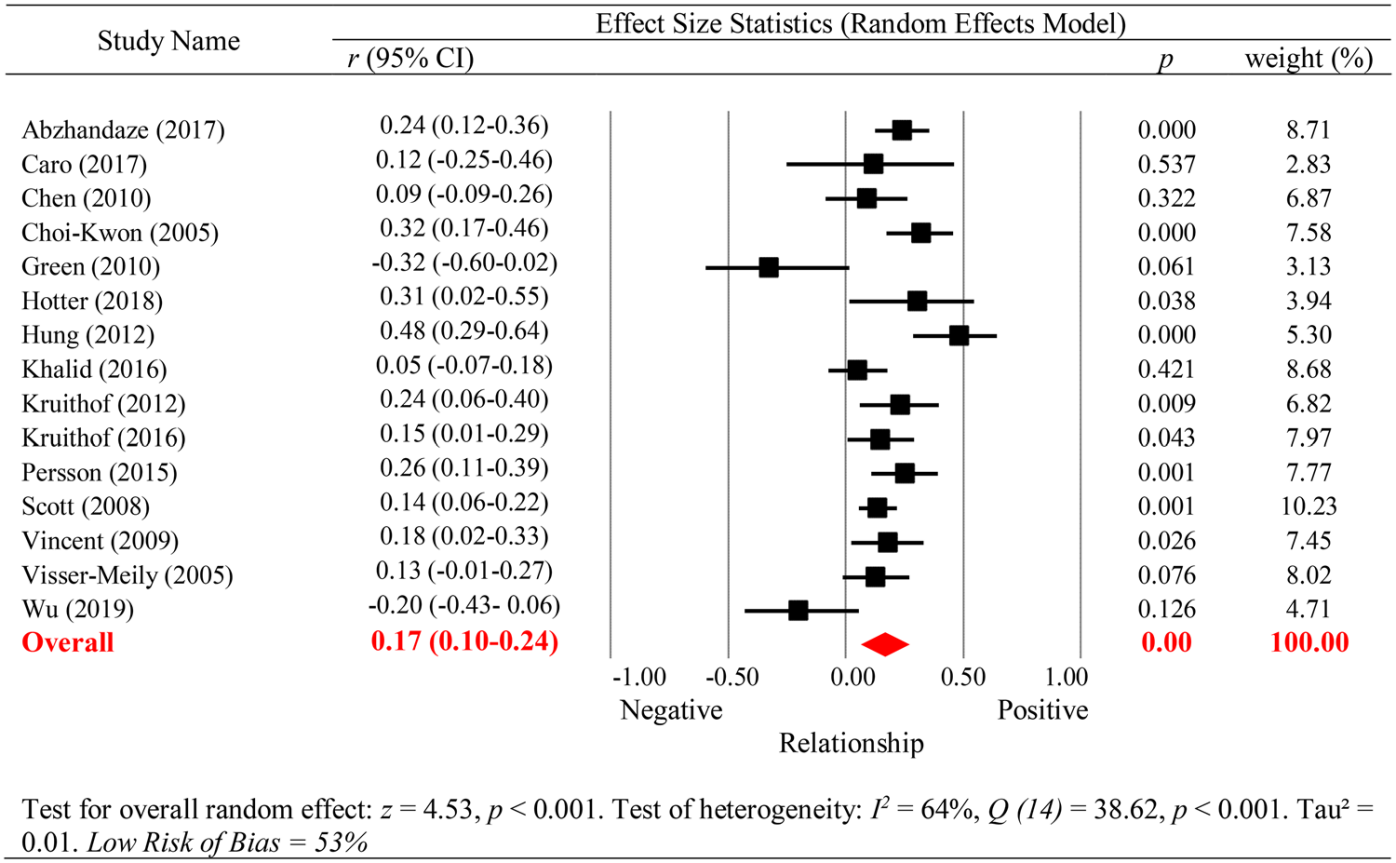
Cognition and Caregiver Outcomes

The observed main effect was moderated by study quality, with studies rated as *low* risk of bias (*N* = 12) demonstrating larger effect sizes, *r* = 0.30 (95% CI 0.23–0.37), than studies rated as *moderate*/*high* risk of bias (*N* = 26), *r* = 0.19 (95% CI 0.14–0.24), *p* = 0.013. The mean age of the stroke survivor, mean time since injury, and timing of the cognitive assessment (i.e. concurrent versus sequential) were not significant moderators (see Supplementary Tables 2 and 3). As noted within the methods section, the majority of included studies measured cognition as a continuous variable, with only five studies using cut points to infer impairment on some measures. Visual inspection of effect sizes statistics (Fig. 2) of these five studies did not reveal any discernible differences in the pattern of results compared to the rest of the included studies. This also applies to cognitive domain-specific analyses (Fig. 3) and caregiver outcome analyses (Fig. 4) below.

The relationship between QoL and individual cognitive domains is depicted in Fig. 3. Two studies were unable to be included in the cognitive domain subgroup analysis: Scott et al. (2008) investigated global cognition (i.e. multiple neuropsychological tests combined) and Jonkman et al. (1998) investigated a decrease in intellectual function (i.e. global cognition in comparison to premorbid estimates of cognitive functioning). All cognitive domains, except language, demonstrated a *small to medium* significant relationship with QoL outcomes for stroke survivors. Those with poorer speed, attention, visuospatial skills, memory, executive skills, and poorer performances on general cognitive screening exhibited lower QoL. Risk of bias (*low* versus *moderate*/*high*), timing of assessment (i.e. concurrent versus sequential), mean age, and mean time since injury did not significantly moderate the relationship between any cognitive domains (i.e. speed, attention, visuospatial, language, memory, or executive function) and QoL in stroke survivors.

### Stroke Survivor Cognitive Function and Caregiver Outcomes

Fifteen studies reported data on caregiver outcomes. Caro and colleagues (2017), Green and King (2010), and Visser-Meily and colleagues (2005) investigated both caregiver burden and caregiver QoL. Therefore, data were available for ten studies investigating burden and eight investigating caregiver QoL. Given the small number of studies, caregiver outcomes (caregiver burden and caregiver QoL) were primarily examined combined, before also examined separately. Cognition was primarily assessed using cognitive screening instruments (80.0%, 12/15 studies). Two studies also assessed language function (Hotter et al., 2018; Vincent et al., 2009), one visuospatial skill (Vincent et al., 2009), one executive skill (Wu et al., 2019), and one investigated global cognition based on neuropsychological assessment (Scott et al., 2008). In sum, there was insufficient power (Jackson & Turner, 2017) to complete separate cognitive domain subgroup analysis for caregiver outcomes.

Overall diminished cognitive functioning (all neuropsychological tests and screening measures combined) had a significant *small to medium* association with poorer overall caregiver outcome (caregiver burden and QoL combined), *r* = 0.17 (95% CI 0.10–0.24), *p* < 0.001 (see Fig. 4). Inspection of funnel plots revealed reasonable symmetry (Egger’s intercept =  − 0.25, *p* = 0.42, one-tailed). Heterogeneity was significant, *I*^2^ = 64%, *Q* (14) = 38.62, *p* < 0.001; τ^2^ = 0.01. The effect was moderated by study quality, with studies rated as *low* risk of bias (*N* = 8) reporting higher effect sizes, *r* = 0.22 (95% CI 0.15–0.30) than those rated as *moderate*/*high* risk of bias (*N* = 7), *r* = 0.07 (95% CI =  − 0.05–0.19), *p* = 0.038. The time point that cognition was assessed (concurrent vs sequential), mean stroke survivor age, mean caregiver age, or mean time since injury were not significant moderators (see Supplementary Tables 2 and 3). Finally, effect sizes were similar when caregiver outcomes (i.e. caregiver burden and caregiver QoL) were analysed separately. Poorer cognitive functioning demonstrated a *small to medium* significant association with higher caregiver burden, *r* = 0.17 (95% CI 0.06–0.29), *p* < 0.001 and lower caregiver QoL,* r* = 0.14 (95% CI 0.06–0.21), *p* < 0.001.

### Reporting Bias and Certainty Assessment

The GRADE framework was used to rate the confidence in the observed effects between cognition and stroke survivor QoL and caregiver outcomes. As this systematic review included observational studies, the starting level of confidence was rated as low. No points were deducted for imprecision, indirectness, or publication bias, or risk of bias. As noted above, moderator analyses revealed that high-quality studies demonstrate a larger effect than low-quality studies. However, there was significant heterogeneity which reduced our confidence based on inconsistency. Overall, the confidence in the overall effect between cognition and stroke survivor QoL and caregiver outcomes was rated as Low.

## Discussion

This systematic literature review and meta-analysis investigated the association between adult stroke survivor cognitive functioning and stroke survivor QoL, caregiver QoL and caregiver burden at least 3 months post-stroke. The review identified 50 studies, spanning the last two decades and 24 countries worldwide. Thirty-eight of the studies reported on the association between cognition and stroke survivor QoL, and 15 reported on the association between cognition and caregiver outcomes. Reduced post-stroke cognition was significantly associated with poorer QoL of stroke survivors and their informal carers and higher caregiver burden, regardless of the mean age of the stroke survivor or caregiver, mean time since injury, or the timing of the cognitive assessments.

There was a small to medium but significant association (*r* = 0.23) between stroke survivor cognition and QoL. Consistent with previous reviews (Watson et al., 2020), individuals with reduced cognitive performance reported lower QoL. That finding has now been replicated utilising a larger number of studies specifically focusing on the stroke population in later stages of recovery (> 3 months) and including both screening and neuropsychological measures of cognition. The finding that cognition is associated with QoL is consistent with observations in other populations including individuals diagnosed with various neurological conditions (Gorgoraptis et al., 2019; Lawson et al., 2014; Mitchell et al., 2010) and older adults (Borowiak & Kostka, 2004; Pan et al., 2015).

Subgroup analysis of multiple cognitive domains found that speed, attention, visuospatial, memory, and executive skills were all important in determining QoL in stroke survivors. Stroke survivors with reduced performance in these domains reported a lower QoL. No significant association was observed between language and QoL. This was surprising as the increasing severity of aphasia is negatively correlated with QoL (Hilari et al., 2012). However, in the current review, measures of language were comprised of basic expressive and receptive function assessments (e.g. Boston Naming Test, Token Test, and language index from the RBANS) which may not capture pragmatic communication difficulties. One finding suggests that language production, rather than comprehension, may have a stronger association with stroke survivor QoL (Dvorak et al., 2021). Furthermore, from a methodological perspective, individuals with greater communication impairments are frequently excluded from studies due to difficulties participating in cognitive testing, completing patient-reported outcome measures, or engaging in informed consent procedures (Brady et al., 2013). It is indeed important to consider the validity of comprehensive neuropsychological assessment, particularly in people with severe language comprehension impairment (Crivelli et al., 2023). To wit, of the eight studies included in this review that assessed language function, five reported procedures for managing individuals with aphasia which included omitting verbal assessments or excluding this population altogether. Aphasia post-stroke is common (16–30% at follow up; Flowers et al. (2016)) and stroke survivors with aphasia often experience other cognitive difficulties (Fonseca et al., 2019). Future research should aim to accommodate individuals with aphasia to provide a more representative sample of stroke survivors.

Cognitive screening measures also demonstrated a significant association with QoL. Cognitive screening tools are widely used in many clinical and research settings, as they are quick to administer, feasible to embed in clinical practice and research protocols, and are generally effective in detecting global post-stroke cognitive difficulties. The observed association emphasises the importance of advocating for cognitive screening as part of routine care to identify reduced post-stroke cognitive functioning and those at risk of poor outcomes. Nevertheless, we also recommend further comprehensive neuropsychological assessment of stroke survivors who present with low cognitive performance on screening instruments or those with subjective (van Rijsbergen et al., 2014) or informant-reported cognitive complaints. This is because many cognitive screening tools have the potential to under- or over-estimate cognition for those with low or high premorbid cognitive function; fail to assess low prevalence stroke-related cognitive impairments (e.g. prosopagnosia); and many are unable to provide a profile of intact and impaired cognitive domains (i.e. produce just one global score/cut-point; Jokinen et al., 2015; Stolwyk et al., 2014). Comprehensive neuropsychological assessments address many of these aforementioned limitations by producing a detailed cognitive profile of cognitive strengths and weaknesses that can be interpreted not only in relation to age-related peers but also compared to a person’s estimated premorbid cognitive function. This provides a more informative characterisation of how a recent stroke may have impacted a person’s specific cognitive functions, which can, in turn, be used as part of a broader biopsychological formulation to address diagnostic considerations and inform ongoing management and further rehabilitation. There are many evidence-based neuropsychological rehabilitation interventions available (Rogers et al., 2018) representing an opportunity to improve patient outcomes. Selecting the best intervention, however, would rely on a thorough understanding of the stroke survivor’s cognitive strengths and weaknesses.

Regarding caregiver outcomes, meta-analysis revealed a small to medium association between stroke survivor cognitive functioning and caregiver outcomes (*r* = 0.17). Caregivers reported lower QoL and higher caregiver burden when providing support to stroke survivors with reduced cognitive ability. In the current review, we identified 15 studies that met inclusion criteria; 10 reported caregiver burden and eight caregiver QoL data, providing a synthesis of evidence from a larger number of studies than previous reviews (Rigby et al., 2009; Zhu & Jiang, 2018). The majority of studies investigating caregiver outcomes (80%) utilised cognitive screening instruments to measure stroke survivor cognition, as opposed to comprehensive neuropsychological evaluation, and so cognitive subdomain analysis was not possible.

This review provides evidence that the cognition of stroke survivors can impact caregiver outcomes. Further research is required to explore this association in more detail using comprehensive neuropsychological assessment to allow for domain-specific analysis which was not possible based on currently available studies. It is possible that some cognitive domains may be more predictive of caregiver outcomes than others due to unique challenges experienced in managing and supporting the stroke survivor. It could be hypothesised, for example, that cognitive functions which particularly impact personality and behaviour (e.g. executive functions and social cognition) may be particularly difficult for caregivers to manage.

In clinical practice, caregivers often receive information and support on managing the physical aspects of stroke recovery, particularly early post-stroke when the focus is on leaving the hospital. However, in an interview-based study, caregivers identified managing cognitive, emotional, and behaviour changes as one of the primary problems in the first-month post-stroke and reported that they were initially unaware or underestimated how cognitive difficulties would impact on safety (e.g. falls; Grant et al., 2004). The current review demonstrates that poorer cognitive functioning post-stroke negatively impacts the caregiver experience, further highlighting the importance of providing caregivers with early education, training, and ongoing access to services to help manage cognitive changes post-stroke.

The current meta-analysis found that study quality moderated the relationship between stroke-survivor cognition, and both stroke-survivor QoL and caregiver outcomes (QoL and burden). Low risk of bias studies demonstrated larger effect sizes than those rated as moderate to high risk of bias; suggesting that with methodologically rigorous study designs, the relationships are more likely to be demonstrated. As lower-quality studies outnumbered higher-quality studies, the current reported association may underestimate the true overall effect. The strength of the relationship between stroke survivor cognitive ability and either stroke survivor QoL or caregiver outcomes was not significantly moderated by mean stroke survivor age, mean caregiver age, mean time point that cognition was assessed (concurrent vs sequential), or mean time since injury. This indicates that cognition is an important variable to consider across age groups of stroke survivors and caregivers and across stages of stroke recovery. It is important to note; however, that we examined these factors as means within each study, and it was not possible to conduct an individual patient data meta-analysis of trajectories of functioning, which may have influenced study findings (see Lo et al., 2022). Overall, these findings highlight the importance of providing ongoing short-term and long-term support to stroke survivors with cognitive difficulties and their informal caregivers to optimise their QoL and mitigate caregiver burden.

## Limitations

Several limitations in the current review are acknowledged. Firstly, the initial aim of this review was to investigate the relationship between cognition and multiple outcomes including quality of life, ADL, participation, institutionalisation, mortality, caregiver burden, and dementia diagnosis. An initial search in March 2021 resulted in a large volume of publications and a lengthy synthesis process that was found to be beyond the scope of a single review and the focus was subsequently narrowed. Therefore, database searches of desired outcomes were repeated at later dates to capture any advancement in the field, ADL and participation outcomes were published elsewhere (Stolwyk et al., 2021), and QoL and caregiver burden were presented. The final search for the current review was completed in May 2023. The authors acknowledge that additional work may have become available subsequently and recommend an ongoing review of literature to update the knowledge base.

Second, while the review identified associations between post-stroke cognition and QoL and caregiver outcomes, these associations do not infer causation. Based on current evidence, we cannot conclude with high certainty that cognition directly impacts these outcomes, as opposed to sharing variance with other variables that may be more directly influencing QoL and burden. For example, there is high-comorbidity between stroke-related cognitive and physical impairments. While some studies in this review reported a unique association between cognition and QoL using regression modelling, other studies using simple correlations were not able to control for potential shared variance with other factors. Future studies using regression-based approaches and including potential confounds such as overall stroke severity and physical impairment are recommended. Studies investigating if ameliorating cognitive difficulties results in improved QoL and caregiver outcomes may also further shed light on this relationship. Further, given the observational nature of study designs; the quality of the studies available (32% and 53% rated as *low* risk of bias for stroke survivor and caregiver outcomes, respectively), and unexplained variability in findings across studies, the confidence in the effect identified in this study is limited. This is reflected in the GRADE ratings (low), and therefore, the recommendations and conclusions are provided with this in mind.

Third, a wide range of tests was used to assess cognition with no clear consensus within the literature on which test should be used to assess a particular cognitive domain. This is likely due to the multifaceted nature of several cognitive domains and the fact that most neuropsychological tests in fact measure multiple cognitive processes. For example, executive functions refer to a set of processes required for complex and goal-directed behaviour, including planning, set-shifting, problem-solving, reasoning, and inhibitory control (Diamond, 2013). There is significant overlap with other cognitive domains such as attention and a range of measures are generally required to be administered to capture this constellation of skills. The authors took a pragmatic approach to test categorisation as described in the Method section, and it is recognised that there will be some differences of opinion on decisions made. It is also recognised that the tests targeting some domains (e.g. visuospatial) are heterogeneous and in fact measure a range of more discrete skills, including neglect, object recognition and visual abstract reasoning. Even tests considered ‘purer’ measures of specific cognitive domains tend to differ in terms of the motor and sensory skills required to complete them. Further delineation of these cognitive constructs in future research is warranted in addition to selecting the most ‘process pure’ tasks where possible and acknowledging the inherent multifaceted nature of our cognitive measures (Kessels, 2019). Further, some authors grouped the same measures under different cognitive domains, reporting only aggregate scores of multiple tests which could not be separated (as denoted by the asterisk in Table 1) or used test batteries. For example, phonemic verbal fluency was grouped with assessments of language by some authors and executive skills by others. If the data for each test could not be separated, the current review identified the domain most closely represented based on classification by the published study and agreement by the research team; this method may result in a less delineation of cognitive domains. A lack of consensus on cognitive test selection and interpretation likely accounts for some of the variability observed across studies (Stolwyk et al., 2021). To increase the interpretability and generalisability of findings, the creation and use of consensus-based assessments and cognitive domain classifications are recommended. In addition, the current review did not include studies utilising measures of social cognition, meta-cognitive skills, or ecologically-based measures of cognition to be able to comment on their impact on stroke survivor or caregiver outcomes (i.e. QoL, burden). It is possible that ecologically-based measures may be more predictive of outcomes and inform targeted interventions (Hogan et al., 2023). Social cognition may also be important in predicting quality of life due to its known impact on emotion processing, social perception, and interpersonal relationships (Adams et al., 2019). It is recommended that future studies consider the inclusion of these tests, in additional to traditional neuropsychological measures, to provide a more comprehensive understanding of the support required for stroke survivors and their carers.

Similarly, studies utilised a wide range of QoL measures which vary widely in the domains encompassed by each instrument (e.g. physical function, role activities, psychological well-being, social functioning, cognitive functioning, and health perceptions). The lack of consensus and standardisation in this field has been identified and critiqued previously (Geyh et al., 2007; Salter et al., 2008) and the differences amongst the QoL measures also likely contributed to some of the variability across studies. The WHO definition of QoL as the individuals’ perceptions of their position in life within their context, goals, and expectations should be in the forefront when selecting QoL instruments to measure outcomes, to avoid merely assessing the presence of impairments (mobility, ADL), symptoms or participation restrictions, but the individual’s satisfaction and perception of their life in relation to their physical, occupational, psychological, and social functioning. With regards to our specific research question, only some studies used a QoL measure containing cognition-related items (e.g. SSQoL, WHOQoL-BREF), with many other measures limited to physical and/or mental-health items (e.g. EuroQoL, SF-36). This may have led to some underestimation of the association between cognition and QoL in our analyses. To optimise methodological rigour, future studies in this field should consider QoL measures that include cognition-related items. There was relatively more consistency in the caregiver burden instruments. Similar to the work within the traumatic brain injury literature (Honan et al., 2019), guidelines and recommendations for test selection and outcome measurement would be beneficial in the stroke population to improve synthesis of evidence and provide stronger and more meaningful clinical and research recommendations.

Finally, our review identified that, overwhelmingly, cognitive screening instruments are used in the literature to measure cognitive functioning. Almost 60% of studies investigating stroke survivor QoL outcomes relied on cognitive screening instruments, and an even larger proportion relied on cognitive screening (80%) when investigating caregiver outcomes. In the context of screening instruments not being as sensitive as a comprehensive neuropsychological assessment to detect impairment and providing a broad overview of cognition with no specific areas for intervention, the clinical recommendations that can be made based on cognitive screening results are limited. Nonetheless, post-stroke cognition is an important variable to consider when identifying the risk of poor outcomes, and interventions and supports are required for the stroke survivor and their informal caregivers. We suggest that neuropsychological rehabilitation interventions should be person-centred and embedded in the everyday experience of the individual, targeting life roles that have the most impact on QoL and incorporating caregiver support and training as a core part of the intervention.

## Conclusion

Predictors of outcome following stroke have historically relied upon physical measurements of stroke severity. The current review provides Level 1 evidence (OCEBM) that post-stroke cognition is not only associated with stroke survivor QoL but also the outcomes of their informal caregivers. Increased clinician awareness of the importance of cognition in determining outcomes is needed, along with embedding early and ongoing cognitive assessment of all stroke survivors within services to inform the provision of support, monitoring, and interventions aimed at stroke survivors and their caregivers. While the current review provides evidence that cognition is associated with stroke survivor QoL and caregiver outcomes (QoL and burden), the efficacy of neuropsychological rehabilitation in improving QoL outcomes and reducing caregiver burden requires further research.

## Other information

### Registration and Protocol

The current systematic literature review and meta-analysis was conducted and reported in accordance with the Preferred Reporting Items for Systematic Reviews and Meta-Analyses (PRISMA) statement (Page et al., 2021). Protocol details for the review were registered with the online International Prospective Register of Systematic Reviews (PROSPERO ID: CRD42018089092) and can be accessed from: https://www.crd.york.ac.uk/prospero/display_record.php?ID=CRD42018089092.

The original protocol database search included multiple outcomes which resulted in a large volume of studies deemed beyond the scope of a single review. Therefore, the relationship between cognition and ADL and participation outcomes was published elsewhere (Stolwyk et al., 2021), and this review focuses on QOL and caregiver outcomes. Outcomes that were excluded are outlined in Fig. 1. Given the large volume of studies, the search was updated on 29th May 2023.

## Supplementary Information

Below is the link to the electronic supplementary material.

Supplementary file1 (DOCX 429 KB)

## Data Availability

The data that support the findings of this study are available from the corresponding author upon request.

## References

[CR1] Abzhandadze, T., Forsberg-Wärleby, G., Holmegaard, L., Redfors, P., Jern, C., Blomstrand, C., & Jood, K. (2017). Life satisfaction in spouses of stroke survivors and control subjects: A 7-year follow-up of participants in the Sahlgrenska Academy study on ischaemic stroke. *Journal of Rehabilitation Medicine,**49*(7), 550–557. 10.2340/16501977-2242 28657641 10.2340/16501977-2242

[CR2] Adamit, T., Maeir, A., Ben Assayag, E., Bornstein, N. M., Korczyn, A. D., & Katz, N. (2015). Impact of first-ever mild stroke on participation at 3 and 6 month post-event: The TABASCO study. *Disability & Rehabilitation,**37*(8), 667–673. 10.3109/09638288.2014.923523 24889677 10.3109/09638288.2014.923523

[CR3] Adams, A. G., Schweitzer, D., Molenberghs, P., & Henry, J. D. (2019). A meta-analytic review of social cognitive function following stroke. *Neuroscience & Biobehavioral Reviews,**102*, 400–416. 10.1016/j.neubiorev.2019.03.011 30922978 10.1016/j.neubiorev.2019.03.011

[CR4] Adamson, J., Beswick, A., & Ebrahim, S. (2004). Is stroke the most common cause of disability? *Journal of Stroke and Cerebrovascular Diseases,**13*(4), 171–177. 10.1016/j.jstrokecerebrovasdis.2004.06.003 17903971 10.1016/j.jstrokecerebrovasdis.2004.06.003

[CR5] Ahlsiö, B., Britton, M., Murray, V., & Theorell, T. (1984). Disablement and quality of life after stroke. *Stroke,**15*(5), 886–890. 10.1161/01.str.15.5.886 6236588 10.1161/01.str.15.5.886

[CR6] Ahmed, T., Kumar, R., & Bahurupi, Y. (2020). Factors affecting quality of life among post-stroke patients in the Sub-Himalayan region. *Journal of Neurosciences in Rural Practice,**11*(4), 616–622. 10.1055/s-0040-1716927 33144800 10.1055/s-0040-1716927 PMC7595802

[CR7] Akpalu, A., Calys-Tagoe, B. N., & Kwei-Nsoro, R. N. (2018). The effect of cognitive impairment on the health-related quality of life among stroke survivors at a major referral hospital in Ghana. *West African Journal of Medicine,**35*(3), 199–203. 30387094

[CR8] Aliyu, S., Ibrahim, A., Saidu, H., & Owolabi, L. (2018). Determinants of health-related quality of life in stroke survivors in Kano, Northwest Nigeria. *Journal of Medicine in the Tropics,**20*(1), 11–16. 10.4103/jomt.jomt_26_17

[CR9] Anderson, C. S., Linto, J., & Stewart-Wynne, E. G. (1995). A population-based assessment of the impact and burden of caregiving for long-term stroke survivors. *Stroke,**26*(5), 843–849. 10.1161/01.STR.26.5.843 7740578 10.1161/01.str.26.5.843

[CR10] Andrew, N. E., Kilkenny, M., Naylor, R., Purvis, T., Lalor, E., Moloczij, N., & Cadilhac, D. A. (2014). Understanding long-term unmet needs in Australian survivors of stroke. *International Journal of Stroke,**9*, 106–112. 10.1111/ijs.12325 25042019 10.1111/ijs.12325

[CR11] Atkins, D., Best, D., Briss, P. A., Eccles, M., Falck-Ytter, Y., Flottorp, S., Guyatt, G. H., Harbour, R. T., Haugh, M. C., Henry, D., Hill, S., Jaeschke, R., Leng, G., Liberati, A., Magrini, N., Mason, J., Middleton, P, Mrukowicz, J., O’Connell, D., Oxman, A. D., . . . , & Zaza, S. (2004). Grading quality of evidence and strength of recommendations. *BMJ, 328*(7454), 1490–1488. 10.1136/bmj.328.7454.1490 10.1136/bmj.328.7454.1490 PMC428525 15205295

[CR12] Atteih, S., Mellon, L., Hall, P., Brewer, L., Horgan, F., Williams, D., & Hickey, A. (2015). Implications of stroke for caregiver outcomes: Findings from the ASPIRE-S study. *International Journal of Stroke,**10*(6), 918–923. 10.1111/ijs.12535 26061711 10.1111/ijs.12535

[CR13] Baddleley, A., Emslie, H., & Nimmo-Smith, I. (1994). *Doors and People*. Thames Valley Test Company.

[CR14] Baek, M. J., Kim, H. J., & Kim, S. (2012). Comparison between the Story Recall Test and the Word-List Learning Test in Korean patients with mild cognitive impairment and early stage of Alzheimer’s disease. *Journal of Clinical and Experimental Neuropsychology,**34*(4), 396–404. 10.1080/13803395.2011.645020 22263656 10.1080/13803395.2011.645020

[CR15] Bakas, T., & Champion, V. (1999). Development and psychometric testing of the Bakas Caregiving Outcomes Scale. *Nursing Research,**48*(5), 250–259. 10.1097/00006199-199909000-00005 10494909 10.1097/00006199-199909000-00005

[CR16] Bakas, T., Champion, V., Perkins, S. M., Farran, C. J., & Williams, L. S. (2006). Psychometric testing of the revised 15-item Bakas Caregiving Outcomes Scale. *Nursing Research,**55*(5), 346–355. 10.1097/00006199-200609000-00007 16980835 10.1097/00006199-200609000-00007

[CR17] Balshem, H., Helfand, M., Schünemann, H. J., Oxman, A. D., Kunz, R., Brozek, J., Vist, G. E., Falck-Ytter, Y., Meerpohl, J., Norris, S., & Guyatt, G. H. (2011). GRADE guidelines: 3. Rating the quality of evidence. *Journal of Clinical Epidemiology, 64*(4), 401–406. 10.1016/j.jclinepi.2010.07.015 10.1016/j.jclinepi.2010.07.015 21208779

[CR18] Barker-Collo, S., Feigin, V. L., Parag, V., Lawes, C. M., & Senior, H. (2010b). Auckland Stroke Outcomes Study. Part 2: Cognition and functional outcomes 5 years poststroke. *Neurology, 75*(18), 1608–1616. 10.1212/WNL.0b013e3181fb44c8 10.1212/WNL.0b013e3181fb44c8 21041784

[CR19] Barker-Collo, S., Feigin, V., Lawes, C., Senior, H., & Parag, V. (2010a). Natural history of attention deficits and their influence on functional recovery from acute stages to 6 months after stroke. *Neuroepidemiology,**35*(4), 255–262. 10.1159/000319894 20881428 10.1159/000319894

[CR20] Bédard, M., Molloy, D. W., Squire, L., Dubois, S., Lever, J. A., & O’Donnell, M. (2001). The Zarit burden interview: A new short version and screening version. *The Gerontologist,**41*(5), 652–657. 10.1093/geront/41.5.652 11574710 10.1093/geront/41.5.652

[CR21] Benton, A. L., Abigail, B., Sivan, A. B., Hamsher, K. d., Varney, N. R., & Spreen, O. (1994). *Contributions to neuropsychological assessment: A clinical manual*: Oxford University Press.

[CR22] Benton, A. L. (1945). A visual retention test for clinical use. *Archives of Neurology & Psychiatry,**54*(3), 212–216. 10.1001/archneurpsyc.1945.02300090051008 10.1001/archneurpsyc.1945.02300090051008 21004267

[CR23] Bergner, M., Bobbitt, R. A., Carter, W. B., & Gilson, B. S. (1981). The sickness impact profile: Development and final revision of a health status measure. *Medical Care*, 787–805. 10.1097/00005650-198108000-00001. 10.1097/00005650-198108000-00001 7278416

[CR24] Borenstein, M., Hedges, L., Higgins, J., & Rothstein, H. (1994) Comprehensive meta-analysis (Version 3.3.070). Biostat: Englewood, NJ USA.

[CR25] Boosman, H., Schepers, V. P. M., Post, M. W. M., & Visser-Meily, J. M. A. (2011). Social activity contributes independently to life satisfaction three years post stroke. *Clinical Rehabilitation,**25*(5), 460–467. 10.1177/0269215510388314 21059668 10.1177/0269215510388314

[CR26] Borowiak, E., & Kostka, T. (2004). Predictors of quality of life in older people living at home and in institutions. *Aging Clinical and Experimental Research,**16*(3), 212–220. 10.1007/BF03327386 15462464 10.1007/BF03327386

[CR27] Bouffioulx, É., Arnould, C., & Thonnard, J.-L. (2008). Satis-stroke: A satisfaction measure of activities and participation in the actual environmental experienced by patients with chronic stroke. *Journal of Rehabilitation Medicine,**40*(10), 836–843. 10.2340/16501977-0272 19242621 10.2340/16501977-0272

[CR28] Bouffioulx, É., Arnould, C., & Thonnard, J.-L. (2011). Satisfaction with activity and participation and its relationships with body functions, activities, or environmental factors in stroke patients. *Archives of Physical Medicine and Rehabilitation,**92*(9), 1404–1410. 10.1016/j.apmr.2011.03.031 21878211 10.1016/j.apmr.2011.03.031

[CR29] Brady, M. C., Fredrick, A., & Williams, B. (2013). People with aphasia: Capacity to consent, research participation and intervention inequalities. *International Journal of Stroke,**8*(3), 193–196. 10.1111/j.1747-4949.2012.00900.x 23130972 10.1111/j.1747-4949.2012.00900.x

[CR30] Brandt, J., Spencer, M., & Folstein, M. (1988). The telephone interview for cognitive status. *Neuropsychiatry, Neuropsychology, and Behavioral Neurology,**1*(2), 111–117.

[CR31] Bugge, C., Hagen, S., & Alexander, H. (2001). Measuring stroke patients’ health status in the early post-stroke phase using the SF36. *International Journal of Nursing Studies,**38*(3), 319–327. 10.1016/S0020-7489(00)00066-3 11245868 10.1016/s0020-7489(00)00066-3

[CR32] Burgess, P. W., & Shallice, T. (1997). *The Hayling and Brixton Tests*. Thames Valley Test Company: Bury St Edmonds, UK.

[CR33] Canadian Stroke Best Practices. (2019). Canadian best practice recommendations for stroke care: Mood, cognition and fatigue following stroke. 6th. Retrieved from https://www.strokebestpractices.ca/recommendations/mood-cognition-and-fatigue-following-stroke

[CR34] Caro, C. C., Mendes, P. V., Costa, J. D., Nock, L. J., & Cruz, D. M. (2017). Independence and cognition post-stroke and its relationship to burden and quality of life of family caregivers. *Topics in Stroke Rehabilitation,**24*(3), 194–199. 10.1080/10749357.2016.1234224 27646977 10.1080/10749357.2016.1234224

[CR35] Chahal, N., Barker-Collo, S., & Feigin, V. (2011). Cognitive and functional outcomes of 5-year subarachnoid haemorrhage survivors: Comparison to matched healthy controls. *Neuroepidemiology,**37*(1), 31–38. 10.1159/000328647 21757962 10.1159/000328647

[CR36] Chang, W. H., Sohn, M. K., Lee, J., Kim, D. Y., Lee, S.-G., Shin, Y.-I., Oh, G.-J., Lee, Y.-S., Joo, M. C., Han, E. Y., Kang, C., & Kim, Y.-H. (2016). Predictors of functional level and quality of life at 6 months after a first-ever stroke: The KOSCO study. *Journal of Neurology,**263*(6), 1166–1177. 10.1007/s00415-016-8119-y 27113602 10.1007/s00415-016-8119-y

[CR37] Chen, Y., Lu, J., Wong, K. S., Mok, V. C. T., Ungvari, G. S., & Tang, W. K. (2010). Health-related quality of life in the family caregivers of stroke survivors. *International Journal of Rehabilitation Research,**33*(3), 232–237. 10.1097/MRR.0b013e328338b04b 20308912 10.1097/MRR.0b013e328338b04b

[CR38] Chiu, H., Lee, H.-C.B., Chung, W. S., & Kwong, P. K. (1994). Reliability and validity of the Cantonese version of Mini-Mental State Examination-A preliminary study. *Hong Kong Journal of Psychiatry,**4*, 25–28.

[CR39] Choi-Kwon, S., Kim, H.-S., Kwon, S. U., & Kim, J. S. (2005). Factors affecting the burden on caregivers of stroke survivors in South Korea. *Archives of Physical Medicine and Rehabilitation,**86*(5), 1043–1048. 10.1016/j.apmr.2004.09.013 15895355 10.1016/j.apmr.2004.09.013

[CR40] Cohen, J. (1988). *Statistical power analysis for the behavioral sciences* (2nd ed.). Lawrence Erlbaum Associates.

[CR41] Colarusso, R. P., & Hammill, D. D. (1972). *Motor-free visual perception test.* Academic Therapy Publications: Novato, CA USA.

[CR42] Coons, S. J., Rao, S., Keininger, D. L., & Hays, R. D. (2000). A comparative review of generic quality-of-life instruments. *PharmacoEconomics,**17*(1), 13–35. 10.2165/00019053-200017010-00002 10747763 10.2165/00019053-200017010-00002

[CR43] Crivelli, D., Spinosa, C., Angelillo, M. T., & Balconi, M. (2023). The influence of language comprehension proficiency on assessment of global cognitive impairment following Acquired Brain Injury: A comparison between MMSE, MoCA and CASP batteries. *Applied Neuropsychology: Adult,**30*(5), 546–551. 10.1080/23279095.2021.1966430 34420468 10.1080/23279095.2021.1966430

[CR44] Cumming, T. B., Brodtmann, A., Darby, D., & Bernhardt, J. (2014). The importance of cognition to quality of life after stroke. *Journal of Psychosomatic Research,**77*(5), 374–379. 10.1016/j.jpsychores.2014.08.009 25217449 10.1016/j.jpsychores.2014.08.009

[CR45] D’Aniello, G. E., Scarpina, F., Mauro, A., Mori, I., Castelnuovo, G., Bigoni, M., Baudo, S., & Molinari, E. (2014). Characteristics of anxiety and psychological well-being in chronic post-stroke patients. *Journal of the Neurological Sciences,**338*(1), 191–196. 10.1016/j.jns.2014.01.005 24439199 10.1016/j.jns.2014.01.005

[CR46] De Renzi, A., & Vignolo, L. A. (1962). Token test: A sensitive test to detect receptive disturbances in aphasics. *Brain,**85*, 665–678. 10.1093/brain/85.4.665 14026018 10.1093/brain/85.4.665

[CR47] Delis, D. C., Kaplan, E., & Kramer, J. H. (2001). *Delis-Kaplan Executive Function System (D–KEFS)*. APA PsycTests.

[CR48] Devlin, N. J., & Brooks, R. (2017). EQ-5D and the EuroQol group: Past, present and future. *Applied Health Economics and Health Policy,**15*(2), 127–137. 10.1007/s40258-017-0310-5 28194657 10.1007/s40258-017-0310-5 PMC5343080

[CR49] Dewey, H. M., Thrift, A. G., Mihalopoulos, C., Carter, R., Macdonell, R. A., McNeil, J. J., & Donnan, G. A. (2002). Informal care for stroke survivors: Results from the North East Melbourne Stroke Incidence Study (NEMESIS). *Stroke,**33*(4), 1028–1033. 10.1161/01.str.0000013067.24300.b0 11935056 10.1161/01.str.0000013067.24300.b0

[CR50] Di Carlo, A. (2009). Human and economic burden of stroke. *Age and Ageing,**38*(1), 4–5. 10.1093/ageing/afn282 19141505 10.1093/ageing/afn282

[CR51] Diamond, A. (2013). Executive functions. *Annual Review of Psychology,**64*, 135–168. 10.1146/annurev-psych-113011-143750 23020641 10.1146/annurev-psych-113011-143750 PMC4084861

[CR52] Dubois, B., Slachevsky, A., Litvan, I., & Pillon, B. (2000). The FAB: A frontal assessment battery at bedside. *Neurology,**55*(11), 1621–1626. 10.1212/WNL.55.11.1621 11113214 10.1212/wnl.55.11.1621

[CR53] Dumont, C., St-Onge, M., Fougeyrollas, P., & Renaud, L.-A. (1998). Le fardeau perçu par les proches de personnes ayant des incapacités physiques. *Canadian Journal of Occupational Therapy,**65*(5), 258–270. 10.1177/000841749806500503

[CR54] Duncan, W. P., Wallace, M. D., Lai, J. S., Johnson, J. D., Embretson, J. S., & Laster, J. L. (1999). The Stroke Impact Scale Version 2.0: Evaluation of reliability, validity, and sensitivity to change. *Stroke, 30*(10), 2131–2140. 10.1161/01.STR.30.10.2131 10.1161/01.str.30.10.2131 10512918

[CR55] Dvorak, E. L., Gadson, D. S., Lacey, E. H., DeMarco, A. T., & Turkeltaub, P. E. (2021). Domains of health-related quality of life are associated with specific deficits and lesion locations in chronic aphasia. *Neurorehabilitation and Neural Repair.,**35*(7), 634–643. 10.1177/15459683211017507 34018866 10.1177/15459683211017507 PMC8225581

[CR56] Flowers, H. L., Skoretz, S. A., Silver, F. L., Rochon, E., Fang, J., Flamand-Roze, C., & Martino, R. (2016). Poststroke aphasia frequency, recovery, and outcomes: A systematic review and meta-analysis. *Archives of Physical Medicine and Rehabilitation,**97*(12), 2188–2201. 10.1016/j.apmr.2016.03.006 27063364 10.1016/j.apmr.2016.03.006

[CR57] Folstein, M. F., Folstein, S. E., & McHugh, P. R. (1975). “Mini-mental state”. A practical method for grading the cognitive state of patients for the clinician. *Journal of Psychiatric Research, 12*(3), 189–198. 10.1016/0022-3956(75)90026-6 10.1016/0022-3956(75)90026-6 1202204

[CR58] Fonseca, J., Raposo, A., & Martins, I. P. (2019). Cognitive functioning in chronic post-stroke aphasia. *Applied Neuropsychology Adult,**26*(4), 355–364. 10.1080/23279095.2018.1429442 29432034 10.1080/23279095.2018.1429442

[CR59] Stroke Foundation. (2021). Clinical Guidelines for Stroke Management. Retrieved from https://informme.org.au/en/Guidelines/Clinical-Guidelines-for-Stroke-Management

[CR60] Fugl-Meyer, A. R., Bränholm, I.-B., & Fugl-Meyer, K. S. (1991). Happiness and domain-specific life satisfaction in adult northern Swedes. *Clinical Rehabilitation,**5*(1), 25–33. 10.1177/026921559100500105

[CR61] Fugl-Meyer, A. R., Melin, R., & Fugl-Meyer, K. S. (2002). Life satisfaction in 18- to 64-year-old Swedes: In relation to gender, age, partner and immigrant status. *Journal of Rehabilitation Medicine,**34*(5), 239. 10.1080/165019702760279242 12392240 10.1080/165019702760279242

[CR62] Geyh, S., Cieza, A., Kollerits, B., Grimby, G., & Stucki, G. (2007). Content comparison of health-related quality of life measures used in stroke based on the International Classification of Functioning, Disability and Health (ICF): A systematic review. *Quality of Life Research,**16*(5), 833–851. 10.1007/s11136-007-9174-8 17294283 10.1007/s11136-007-9174-8

[CR63] Given, C. W., Given, B., Stommel, M., Collins, C., King, S., & Franklin, S. (1992). The caregiver reaction assessment (CRA) for caregivers to persons with chronic physical and mental impairments. *Research in Nursing & Health,**15*(4), 271–283. 10.1002/nur.4770150406 1386680 10.1002/nur.4770150406

[CR64] Goh, H.-T., Tan, M.-P., Mazlan, M., Abdul-Latif, L., & Subramaniam, P. (2019). Social participation determines quality of life among urban-dwelling older adults with stroke in a developing country. *Journal of Geriatric Physical Therapy,**42*(4), E77–E84. 10.1519/JPT.0000000000000196 29851747 10.1519/JPT.0000000000000196

[CR65] Golomb, B. A., Vickrey, B. G., & Hays, R. D. (2001). A review of health-related quality-of-life measures in stroke. *PharmacoEconomics,**19*(2), 155–185. 10.2165/00019053-200119020-00004 11284381 10.2165/00019053-200119020-00004

[CR66] Goodglass, H., Kaplan, E., & Barresi, B. (2000). *Boston Diagnostic Aphasia Examination-Third Edition (BDAE-3)*. Lippincott.

[CR67] Gorgoraptis, N., Zaw-Linn, J., Feeney, C., Tenorio-Jimenez, C., Niemi, M., Malik, A., Ham, T., Goldstone, A. P., & Sharp, D. J. (2019). Cognitive impairment and health-related quality of life following traumatic brain injury. *NeuroRehabilitation,**44*, 321–331. 10.3233/NRE-182618 31177238 10.3233/NRE-182618

[CR68] Graessel, E., Berth, H., Lichte, T., & Grau, H. (2014). Subjective caregiver burden: Validity of the 10-item short version of the Burden Scale for Family Caregivers BSFC-s. *BMC Geriatrics,**14*(1), 23–23. 10.1186/1471-2318-14-23 24555474 10.1186/1471-2318-14-23 PMC3942019

[CR69] Grant, J. S., Glandon, G. L., Elliott, T. R., Giger, J. N., & Weaver, M. (2004). Caregiving problems and feelings experienced by family caregivers of stroke survivors the first month after discharge. *International Journal of Rehabilitation Research,**27*(2), 105–111. 10.1097/01.mrr.0000127639.47494.e3 15167107 10.1097/01.mrr.0000127639.47494.e3

[CR70] Green, T. L., & King, K. M. (2010). Functional and psychosocial outcomes 1 year after mild stroke. *Journal of Stroke & Cerebrovascular Diseases,**19*(1), 10–16. 10.1016/j.jstrokecerebrovasdis.2009.02.005 20123221 10.1016/j.jstrokecerebrovasdis.2009.02.005

[CR71] Gronwall, D. (1977). Paced auditory serial-addition task: A measure of recovery from concussion. *Perceptual and Motor Skills,**44*, 367–373. 10.2466/pms.1977.44.2.367 866038 10.2466/pms.1977.44.2.367

[CR72] Guyatt, G. H., Oxman, A. D., Kunz, R., Vist, G. E., Falck-Ytter, Y., & Schünemann, H. J. (2008). What is “quality of evidence” and why is it important to clinicians? *BMJ,**336*(7651), 995–998. 10.1136/bmj.39490.551019.BE 18456631 10.1136/bmj.39490.551019.BE PMC2364804

[CR73] Haley, W. E., Roth, D. L., Hovater, M., & Clay, O. J. (2015). Long-term impact of stroke on family caregiver well-being: A population-based case-control study. *Neurology,**84*(13), 1323–1329. 10.1212/WNL.0000000000001418 25740862 10.1212/WNL.0000000000001418 PMC4388745

[CR74] Hall, K. S., Gao, S., Emsley, C. L., Ogunniyi, A. O., Morgan, O., & Hendrie, H. C. (2000). Community screening interview for dementia (CSI ‘D’); performance in five disparate study sites. *International Journal of Geriatric Psychiatry,**15*(6), 521–531. 10.1002/1099-1166(200006)15:6%3c521::AID-GPS182%3e3.0.CO;2-F 10861918 10.1002/1099-1166(200006)15:6<521::aid-gps182>3.0.co;2-f

[CR75] Hayden, J. A., Côté, P., & Bombardier, C. (2006). Evaluation of the quality of prognosis studies in systematic reviews. *Annals of Internal Medicine,**144*(6), 427–437. 10.7326/0003-4819-144-6-200603210-00010 16549855 10.7326/0003-4819-144-6-200603210-00010

[CR76] Hayden, J. A., Tougas, M. E., Riley, R., Iles, R., & Pincus, T. (2014). Individual recovery expectations and prognosis of outcomes in non-specific low back pain: Prognostic factor exemplar review. *Cochrane Database of Systematic Reviews*. 10.1002/14651858.CD011284 10.1002/14651858.CD011284.pub2 PMC6877336 31765487

[CR77] Higgins, J. P. T., & Thompson, S. G. (2002). Quantifying heterogeneity in a meta-analysis. *Statistics in Medicine,**21*(11), 1539–1558. 10.1002/sim.1186 12111919 10.1002/sim.1186

[CR78] Higgins, J. P. T., Thompson, S. G., Deeks, J. J., & Altman, D. G. (2003). Measuring inconsistency in meta-analyses. *British Medical Journal,**327*(7414), 557–560. 10.1136/bmj.327.7414.557 12958120 10.1136/bmj.327.7414.557 PMC192859

[CR79] Hilari, K., Lamping, D. L., Smith, S. C., Northcott, S., Lamb, A., & Marshall, J. (2009). Psychometric properties of the Stroke and Aphasia Quality of Life Scale (SAQOL-39) in a generic stroke population. *Clinical Rehabilitation,**23*(6), 544–557. 10.1177/0269215508101729 19447841 10.1177/0269215508101729

[CR80] Hilari, K., Needle, J. J., & Harrison, K. L. (2012). What are the important factors in health-related quality of life for people with aphasia? A systematic review. *Archives of Physical Medicine and Rehabilitation,**93*(1), S86–S95. 10.1016/j.apmr.2011.05.028 22119074 10.1016/j.apmr.2011.05.028

[CR81] Hochstenbach, J. B., Anderson, P. G., van Limbeek, J., & Mulder, T. T. (2001). Is there a relation between neuropsychologic variables and quality of life after stroke? *Archives of Physical Medicine and Rehabilitation,**82*(10), 1360–1366. 10.1053/apmr.2001.25970 11588738 10.1053/apmr.2001.25970

[CR82] Hogan, C., Cornwell, P., Fleming, J., Man, D. W. K., & Shum, D. H. K. (2023). Assessment of prospective memory after stroke utilizing virtual reality. *Virtual Reality,**27*(1), 333–346. 10.1007/s10055-021-00576-5

[CR83] Honan, C. A., McDonald, S., Tate, R., Ownsworth, T., Togher, L., Fleming, J., Anderson, V., Morgan, A., Catroppa, C., Douglas, J., Francis, H., Wearne, T., Sigmundsdottir, L., & Ponsford, J. (2019). Outcome instruments in moderate-to-severe adult traumatic brain injury: Recommendations for use in psychosocial research. *Neuropsychological Rehabilitation,**29*(6), 896–916. 10.1080/09602011.2017.1339616 28671050 10.1080/09602011.2017.1339616

[CR84] Hotter, B., Padberg, I., Liebenau, A., Knispel, P., Heel, S., Steube, D., Wissel, J., Wellwood, I., & Meisel, A. (2018). Identifying unmet needs in long-term stroke care using in-depth assessment and the Post-Stroke Checklist-The Managing Aftercare for Stroke (MAS-I) study. *European Stroke Journal,**3*(3), 237–245. 10.1177/2396987318771174 31008354 10.1177/2396987318771174 PMC6453198

[CR85] Howitt, S. C., Jones, M. P., Jusabani, A., Gray, W. K., Aris, E., Mugusi, F., Swai, M., & Walker, R. W. (2011). A cross-sectional study of quality of life in incident stroke survivors in rural northern Tanzania. *Journal of Neurology,**258*(8), 1422–1430. 10.1007/s00415-011-5948-6 21336782 10.1007/s00415-011-5948-6

[CR86] Hung, J. W., Huang, Y. C., Chen, J. H., Liao, L. N., Lin, C. J., Chuo, C. Y., & Chang, K. C. (2012). Factors associated with strain in informal caregivers of stroke patients. *Chang Gung Medical Journal,**35*(5), 392–401. 10.4103/2319-4170.105479 23127344 10.4103/2319-4170.105479

[CR87] Hunt, S. M., McEwen, J., & McKenna, S. P. (1985). Measuring health status: A new tool for clinicians and epidemiologists. *The Journal of the Royal College of General Practitioners,**35*(273), 185–188. 3989783 PMC1960139

[CR88] Veritas Health Innovation. Covidence systematic review software. Melbourne, Australia. Available at www.covidence.org

[CR89] Jackson, D., & Turner, R. (2017). Power analysis for random-effects meta-analysis. *Research Synthesis Methods,**8*(3), 290–302. 10.1002/jrsm.1240 28378395 10.1002/jrsm.1240 PMC5590730

[CR90] Jaillard, A., Naegele, B., Trabucco-Miguel, S., LeBas, J. F., & Hommel, M. (2009). Hidden dysfunctioning in subacute stroke. *Stroke,**40*(7), 2473–2479. 10.1161/strokeaha.108.541144 19461036 10.1161/STROKEAHA.108.541144

[CR91] Jokinen, H., Melkas, S., Ylikoski, R., Pohjasvaara, T., Kaste, M., Erkinjuntti, T., & Hietanen, M. (2015). Post-stroke cognitive impairment is common even after successful clinical recovery. *European Journal of Neurology,**22*(9), 1288–1294. 10.1111/ene.12743 26040251 10.1111/ene.12743

[CR92] Jonkman, E. J., de Weerd, A. W., & Vrijens, N. L. H. (1998). Quality of life after a first ischemic stroke. *Acta Neurologica Scandinavica,**98*(3), 169–175. 10.1111/j.1600-0404.1998.tb07289.x 9786613 10.1111/j.1600-0404.1998.tb07289.x

[CR93] Kang, Y., & Na, D. L. (2004). *Seoul verbal learning test*. Human Brain Research & Consulting: Seoul, South Korea.

[CR94] Kang, Y., Na, D. A., & Hanh, S. (1997). A validity study on the Korean mini-mental state examination (K-MMSE) in dementia patients. *Journal of the Korean Neurological Association,**15*(2), 300–308.

[CR95] Kaplan, E., Goodglass, H., & Weintraub, S. (2001). *Boston naming test (2nd Ed).* Pro-ed: Austin TX USA.

[CR96] Kertesz, A. (2006). *Western Aphasia Battery-Revised (WAB-R): Examiner’s manual*. Harcourt Assessment Incorporation: Philadelphia, PA USA.

[CR97] Kessels, R. P. C. (2019). Improving precision in neuropsychological assessment: Bridging the gap between classic paper-and-pencil tests and paradigms from cognitive neuroscience. *The Clinical Neuropsychologist,**33*(2), 357–368. 10.1080/13854046.2018.1518489 30394172 10.1080/13854046.2018.1518489

[CR98] Khalid, W., Rozi, S., Ali, T. S., Azam, I., Mullen, M. T., Illyas, S., Un-Nisa, Q., Soomro, N., & Kamal, A. K. (2016). Quality of life after stroke in Pakistan. *BMC Neurology,**16*(1), 250–250. 10.1186/s12883-016-0774-1 27912744 10.1186/s12883-016-0774-1 PMC5135839

[CR99] Kruithof, W. J., Post, M. W. M., van Mierlo, M. L., van den Bos, G. A. M., de Man-van Ginkel, J. M., & Visser-Meily, J. M. A. (2016). Caregiver burden and emotional problems in partners of stroke patients at two months and one year post-stroke: Determinants and prediction. *Patient Education and Counseling,**99*(10), 1632–1640. 10.1016/j.pec.2016.04.007 27103190 10.1016/j.pec.2016.04.007

[CR100] Kruithof, W. J., Visser-Meily, J. M. A., & Post, M. W. M. (2012). Positive caregiving experiences are associated with life satisfaction in spouses of stroke survivors. *Journal of Stroke & Cerebrovascular Diseases,**21*(8), 801–807. 10.1016/j.jstrokecerebrovasdis.2011.04.011 21640607 10.1016/j.jstrokecerebrovasdis.2011.04.011

[CR101] Kwa, V. I., Limburg, M., & de Haan, R. J. (1996). The role of cognitive impairment in the quality of life after ischaemic stroke. *Journal of Neurology,**243*(8), 599–604. 10.1007/BF00900948 8865027 10.1007/BF00900948

[CR102] Kwok, T., Lo, R. S., Wong, E., Wai-Kwong, T., Mok, V., & Kai-Sing, W. (2006). Quality of life of stroke survivors: A 1-year follow-up study. *Archives of Physical Medicine and Rehabilitation,**87*(9), 1177–1182. 10.1016/j.apmr.2006.05.015 16935051 10.1016/j.apmr.2006.05.015

[CR103] Larson, E. B., Kirschner, K., Bode, R. K., Heinemann, A. W., Clorfene, J., & Goodman, R. (2003). Brief cognitive assessment and prediction of functional outcome in stroke. *Topics in Stroke Rehabilitation,**9*(4), 10–21. 10.1310/84yn-y640-8ueq-pdnv 14523696 10.1310/84YN-Y640-8UEQ-PDNV

[CR104] Lawson, R. A., Yarnall, A. J., Duncan, G. W., Khoo, T. K., Breen, D. P., Barker, R. A., Collerton, D., Tayor, J.-P., & Burn, D. J. (2014). Severity of mild cognitive impairment in early Parkinson’s disease contributes to poorer quality of life. *Parkinsonism & Related Disorders,**20*(10), 1071–1075. 10.1016/j.parkreldis.2014.07.004 25074728 10.1016/j.parkreldis.2014.07.004 PMC4194347

[CR105] Lezak, M. D. (2012). *Neuropsychological assessment* (5th ed.). Oxford University Press: New York.

[CR106] Lo, J. W., Crawford, J. D., Desmond, D. W., Bae, H. J., Lim, J. S., Godefroy, O., Roussel, M., Kang, Y., Jahng, S., Köhler, S., Staals, J., Verhey, F., Chen, C., Xu, X., Chong, E. J., Kandiah, N., Yatawara, C., Bordet, R., Dondaine, T., Mendyk, A. M., … Stroke and Cognition (STROKOG) Collaboration (2022). Long-term cognitive decline after stroke: An individual participant data meta-analysis. *Stroke*, 53(4), 1318–1327. 10.1161/STROKEAHA.121.035796 10.1161/STROKEAHA.121.035796 34775838

[CR107] Lopez-Cancio, E., Jovin, T. G., Cobo, E., Cerda, N., Jimenez, M., Gomis, M., Hernandez-Perez, M., Caceres, C., Cardona, P., Lara, B., Renu, A., Llull, L., Boned, S., Muchada, M., & Davalos, A. (2017). Endovascular treatment improves cognition after stroke: A secondary analysis of REVASCAT trial. *Neurology,**88*(3), 245–251. 10.1212/WNL.0000000000003517 27940648 10.1212/WNL.0000000000003517 PMC5272792

[CR108] MacLeod, C. M. (1991). Half a century of research on the Stroop effect: An integrative review. *Psychological Bulletin,**109*(2), 163–203. 10.1037/0033-2909.109.2.163 2034749 10.1037/0033-2909.109.2.163

[CR109] McDowd, J. M., Filion, D. L., Pohl, P. S., Richards, L. G., & Stiers, W. (2003). Attentional abilities and functional outcomes following stroke. *The Journals of Gerontology Series b: Psychological Sciences and Social Sciences,**58*(1), 45–53. 10.1093/geronb/58.1.P45 10.1093/geronb/58.1.p45 12496301

[CR110] Mendis, S. (2013). Stroke disability and rehabilitation of stroke: World Health Organization perspective. *International Journal of Stroke,**8*(1), 3–4. 10.1111/j.1747-4949.2012.00969.x 23280261 10.1111/j.1747-4949.2012.00969.x

[CR111] Meyer, B., Ringel, F., Winter, Y., Spottke, A., Gharevi, N., Dams, J., Balzer-Geldsetzer, M., Mueller, I. K., Klockgether, T., Schramm, J., Urbach, H., & Dodel, R. (2010). Health-related quality of life in patients with subarachnoid haemorrhage. *Cerebrovascular Diseases,**30*(4), 423–431. 10.1159/000317078 20720412 10.1159/000317078

[CR112] Middleton, L. E., Lam, B., Fahmi, H., Black, S. E., McIlroy, W. E., Stuss, D. T., Danells, C., Ween, J., & Turner, G. R. (2014). Frequency of domain-specific cognitive impairment in sub-acute and chronic stroke. *NeuroRehabilitation,**34*(2), 305–312. 10.3233/nre-131030 24401826 10.3233/NRE-131030

[CR113] Mitchell, A. J., Kemp, S., Benito-León, J., & Reuber, M. (2010). The influence of cognitive impairment on health-related quality of life in neurological disease. *Acta Neuropsychiatrica,**22*(1), 2–13. 10.1111/j.1601-5215.2009.00439.x

[CR114] Mole, J. A., & Demeyere, N. (2020). The relationship between early post-stroke cognition and longer term activities and participation: A systematic review. *Neuropsychological Rehabilitation,**30*(2), 346–370. 10.1080/09602011.2018.1464934 29712538 10.1080/09602011.2018.1464934

[CR115] Montgomery, R. J. V., Stull, D. E., & Borgatta, E. F. (1985). Measurement and the analysis of burden. *Research on Aging,**7*(1), 137–152. 10.1177/0164027585007001007 4059631 10.1177/0164027585007001007

[CR116] Nasreddine, Z. S., Phillips, N. A., Bédirian, V., Charbonneau, S., Whitehead, V., Collin, I., Cummings, J. L., & Chertkow, H. (2005). The Montreal Cognitive Assessment, MoCA: A brief screening tool for mild cognitive impairment. *Journal of the American Geriatrics Society,**53*(4), 695–699. 10.1111/j.1532-5415.2005.53221.x 15817019 10.1111/j.1532-5415.2005.53221.x

[CR117] Nys, G. M., van Zandvoort, M. J., de Kort, P. L., van der Worp, H. B., Jansen, B. P., & Algra, A.,de Haan, E. H., & Kappelle, L. J. (2005). The prognostic value of domain-specific cognitive abilities in acute first-ever stroke. *Neurology,**64*(5), 821–827. 10.1212/01.WNL.0000152984.28420.5A 15753416 10.1212/01.WNL.0000152984.28420.5A

[CR118] OCEBM Levels of Evidence Working Group. “The Oxford Levels of Evidence 2”. *Oxford Centre for Evidence-Based Medicine*. http://www.cebm.net/index.aspx?o=5653

[CR119] Ones, K., Yilmaz, E., Cetinkaya, B., & Caglar, N. (2005). Quality of life for patients poststroke and the factors affecting it. *Journal of Stroke & Cerebrovascular Diseases,**14*(6), 261–266. 10.1016/j.jstrokecerebrovasdis.2005.07.003 17904035 10.1016/j.jstrokecerebrovasdis.2005.07.003

[CR120] Osterrieth, P. A. (1944). Le test de copie d’une figure complexe: Contribution à l’étude de la perception et de la mémoire. *Archives De Psychologie,**30*, 286–356.

[CR121] Owolabi, M. O., & Ogunniyi, A. (2009). Profile of health-related quality of life in Nigerian stroke survivors. *European Journal of Neurology,**16*(1), 54–62. 10.1111/j.1468-1331.2008.02339.x 19087151 10.1111/j.1468-1331.2008.02339.x

[CR122] Page, M. J., McKenzie, J. E., Bossuyt, P. M., Boutron, I., Hoffmann, T. C., Mulrow, C. D., Shamseer, L., Tetzlaff, J. M., Akl, E. A., Brennan, S. E., Chou, R., Glanville, J., Grimshaw, J. M., Hróbjartsson, A., Lalu, M. M., Li, T., Loder, E. W., Mayo-Wilson, E., McDonald, S., McGuinness, L. A., ..., & Moher, D. (2021). The PRISMA 2020 statement: An updated guideline for reporting systematic reviews. *BMJ, 372*, 71. 10.1136/bmj.n71 10.1136/bmj.n71 PMC8005924 33782057

[CR123] Pan, C.-W., Wang, X., Ma, Q., Sun, H.-P., Xu, Y., & Wang, P. (2015). Cognitive dysfunction and health-related quality of life among older Chinese. *Scientific Reports,**5*(1), 17301. 10.1038/srep17301 26601612 10.1038/srep17301 PMC4658548

[CR124] Parag, V., Hackett, M. L., Yapa, C. M., Kerse, N., McNaughton, H., Feigin, V. L., & Anderson, C. S. (2008). The impact of stroke on unpaid caregivers: Results from the Auckland regional community stroke study, 2002–2003. *Cerebrovascular Diseases,**25*(6), 548–554. 10.1159/000131673 18480608 10.1159/000131673

[CR125] Park, J. H., Kim, B. J., Bae, H.-J., Lee, J., Lee, J., Han, M.-K., & O, K. Y., Park, S. H., Kang, Y., Yu, K-H., & Lee, B-C. (2013). Impact of post-stroke cognitive impairment with no dementia on health-related quality of life. *Journal of Stroke,**15*(1), 49–56. 10.5853/jos.2013.15.1.49 24324939 10.5853/jos.2013.15.1.49 PMC3779672

[CR126] Passier, P., Visser-Meily, J., Van Zandvoort, M., Rinkel, G., Lindeman, E., & Post, M. (2012). Predictors of long-term health-related quality of life in patients with aneurysmal subarachnoid hemorrhage. *NeuroRehabilitation,**30*(2), 137–145. 10.3233/NRE-2012-0737 22430579 10.3233/NRE-2012-0737

[CR127] Patel, M. D., McKevitt, C., Lawrence, E., Rudd, A. G., & Wolfe, C. D. A. (2007). Clinical determinants of long-term quality of life after stroke. *Age and Ageing,**36*(3), 316–322. 10.1093/ageing/afm014 17374601 10.1093/ageing/afm014

[CR128] Patrick, D. L. (2014). *Functional limitations profile*. In A. C. Michalos (Ed.), Encyclopedia of quality of life and well-being research. Dordrecht. 10.1007/978-94-007-0753-5_1100

[CR129] Persson, J., Holmegaard, L., Karlberg, I., Redfors, P., Jood, K., Jern, C., Blomstrand, C., & Forsberg-Wärleby, G. (2015). Spouses of stroke survivors report reduced health-related quality of life even in long-term follow-up: Results from Sahlgrenska Academy study on ischemic stroke. *Stroke,**46*(9), 2584–2590. 10.1161/STROKEAHA.115.009791 26294675 10.1161/STROKEAHA.115.009791

[CR130] Randolph, C., Tierney, M. C., Mohr, E., & Chase, T. N. (1998). The Repeatable Battery for the Assessment of Neuropsychological Status (RBANS): Preliminary clinical validity. *Journal of Clinical & Experimental Neuropsychology,**20*(3), 310–319. 10.1076/jcen.20.3.310.823 9845158 10.1076/jcen.20.3.310.823

[CR131] Raven, J., & Raven, J. (2003). *Raven progressive matrices*. In R. S. McCallum (Ed.), Handbook of nonverbal assessment: Kluwer Academic/Plenum Publishers. 10.1007/978-1-4615-0153-4_11

[CR132] Reimer, S. O., & W. J., De Haan, R. J., Pijnenborg, J. M. A., Limburg, M., & Van Den Bos, G. A. M. (1998). Assessment of burden in partners of stroke patients with the sense of competence questionnaire. *Stroke,**29*(2), 373–379. 10.1161/01.STR.29.2.373 9472877 10.1161/01.str.29.2.373

[CR133] Reitan, R. M. (1955). The relation of the trail making test to organic brain damage. *Journal of Consulting Psychology,**19*(5), 393. 13263471 10.1037/h0044509

[CR134] Renjen, P. N., Gauba, C., & Chaudhari, D. (2015). *Cognitive Impairment after Stroke. Cureus,**7*(9), e335. 10.7759/cureus.335 26543693 10.7759/cureus.335 PMC4627858

[CR135] Rey, A. (1941). L’examen psychologique dans les cas d’encéphalopathie traumatique. *Archives De Psychologie,**28*, 286–340.

[CR136] Rey, A. (1964). *L’examen clinique en psychologie (The Clinical Psychological Examination)*. Presse Universitaires de France.

[CR137] Rigby, H., Gubitz, G., & Phillips, S. (2009). A systematic review of caregiver burden following stroke. *International Journal of Stroke,**4*(4), 285–292. 10.1111/j.1747-4949.2009.00289.x 19689757 10.1111/j.1747-4949.2009.00289.x

[CR138] Roach, A., Schwartz, M. F., Martin, N., Grewal, R. S., & Brecher, A. (1996). The Philadelphia Naming Test: Scoring and rationale. *Clinical Aphasiology,**24*, 121–133.

[CR139] Robinson, B. C. (1983). Validation of a Caregiver Strain Index. *Journal of Gerontology,**38*(3), 344–348. 10.1093/geronj/38.3.344 6841931 10.1093/geronj/38.3.344

[CR140] Rogers, J. M., Foord, R., Stolwyk, R. J., Wong, D., & Wilson, P. H. (2018). General and domain-specific effectiveness of cognitive remediation after stroke: Systematic literature review and meta-analysis. *Neuropsychology Review,**28*(3), 285–309. 10.1007/s11065-018-9378-4 30006801 10.1007/s11065-018-9378-4

[CR141] Rohde, D., Gaynor, E., Large, M., Mellon, L., Hall, P., Brewer, L., Bennett, K., Williams, D., Dolan, E., Callaly, E., & Hickey, A. (2019). The impact of cognitive impairment on poststroke outcomes: A 5-year follow-up. *Journal of Geriatric Psychiatry and Neurology,**32*(5), 275–281. 10.1177/0891988719853044 31167593 10.1177/0891988719853044

[CR142] Roth, M., Tym, E., Mountjoy, C. Q., Huppert, F. A., Hendrie, H., Verma, S., & Goddard, R. (1986). CAMDEX. A standardised instrument for the diagnosis of mental disorder in the elderly with special reference to the early detection of dementia. *The British Journal of Psychiatry,**149*(6), 698–709. 10.1192/bjp.149.6.698 3790869 10.1192/bjp.149.6.698

[CR143] Royal College of Physicians. (2016). National Clinical Guideline for Stroke. 5th. Retrieved from https://www.rcplondon.ac.uk/guidelines-policy/stroke-guidelines

[CR144] Safaz, İ, Kesikburun, S., Adigüzel, E., & Yilmaz, B. (2016). Determinants of disease-specific health-related quality of life in Turkish stroke survivors. *International Journal of Rehabilitation Research,**39*(2), 130–133. 10.1097/MRR.0000000000000156 26795717 10.1097/MRR.0000000000000156

[CR145] Salter, K. L., Moses, M. B., Foley, N. C., & Teasell, R. W. (2008). Health-related quality of life after stroke: What are we measuring? *International Journal of Rehabilitation Research,**31*(2), 111–117. 10.1097/MRR.0b013e3282fc0f33 18467925 10.1097/MRR.0b013e3282fc0f33

[CR146] Schulz, R., & Beach, S. R. (1999). Caregiving as a risk factor for mortality: The caregiver health effects study. *JAMA,**282*(23), 2215–2219. 10.1001/jama.282.23.2215 10605972 10.1001/jama.282.23.2215

[CR147] Scott, R. B., Eccles, F., Lloyd, A., & Carpenter, K. (2008). From multidimensional neuropsychological outcomes to a cognitive complication rate: The International Subarachnoid Aneurysm Trial. *Trials,**9*, 13. 10.1186/1745-6215-9-13 18341689 10.1186/1745-6215-9-13 PMC2322951

[CR148] Sivan, A. B. (1992). *Benton visual retention test*: Psychological Corporation. San Antonio TX USA.

[CR149] Sousa, F., Rocha, V., Estima, C., Castro, S. L., & Guerra, M. P. (2020). Cognitive deficits, social support, depression and quality of life of post-stroke patients. *Análise Psicológica,**38*(2), 153–165. 10.14417/ap.1726

[CR150] Springer, M. V., Schmidt, J. M., Wartenberg, K. E., Frontera, J. A., Badjatia, N., & Mayer, S. A. (2009). Predictors of global cognitive impairment 1 year after subarachnoid hemorrhage. *Neurosurgery,**65*(6), 1043–1050. 10.1227/01.NEU.0000359317.15269.20 19934963 10.1227/01.NEU.0000359317.15269.20

[CR151] Stanford, J. A., & Turner, A. (2000). *Integrated visual auditory continuous performance*. Brain Train.

[CR152] Stolwyk, R. J., Mihaljcic, T., Wong, D. K., Chapman, J. E., & Rogers, J. M. (2021). Poststroke cognitive impairment negatively impacts activity and participation outcomes: A systematic review and meta-analysis. *Stroke,**52*(2), 748–760. 10.1161/STROKEAHA.120.032215 33493048 10.1161/STROKEAHA.120.032215

[CR153] Stolwyk, R. J., O’Neill, M. H., McKay, A. J. D., & Wong, D. K. (2014). Are cognitive screening tools sensitive and specific enough for use after stroke?: A systematic literature review. *Stroke,**45*(10), 3129–3134. 10.1161/STROKEAHA.114.004232 25074518 10.1161/STROKEAHA.114.004232

[CR154] Sturm, J. W., Donnan, G. A., Dewey, H. M., Macdonell, R. A. L., Gilligan, A. K., Srikanth, V., & Thrift, A. G. (2004). Quality of life after stroke. *Stroke,**35*(10), 2340–2345. 10.1161/01.STR.0000141977.18520.3b 15331799 10.1161/01.STR.0000141977.18520.3b

[CR155] Tang, W. K. M. D., Lau, C. G. M., Mok, V. M. D., Ungvari, G. S. M. D., & Wong, K.-S.M.D. (2013). Impact of anxiety on health-related quality of life after stroke: A cross-sectional study. *Archives of Physical Medicine and Rehabilitation,**94*(12), 2535–2541. 10.1016/j.apmr.2013.07.012 23911556 10.1016/j.apmr.2013.07.012

[CR156] Taylor, E. M. (1959). *Psychological appraisal of children with cerebral defects*. Harvard University Press.

[CR157] The EuroQoL Research Foundation. (1987). *EQ-5D Instruments*. EuroQol Office: Rotterdam, Netherlands.

[CR158] The WHOQOL Group. (1998). Development of the World Health Organization WHOQOL-BREF quality of life assessment. *Psychological Medicine,**28*(3), 551–558. 10.1017/S0033291798006667 9626712 10.1017/s0033291798006667

[CR159] van Rijsbergen, M. W. A., Mark, R. E., de Kort, P. L. M., & Sitskoorn, M. M. (2014). Subjective cognitive complaints after stroke: A systematic review. *Journal of Stroke and Cerebrovascular Diseases,**23*(3), 408–420. 10.1016/j.jstrokecerebrovasdis.2013.05.003 23800498 10.1016/j.jstrokecerebrovasdis.2013.05.003

[CR160] van Straten, A., De Haan, R. J., Limburg, M., Schuling, J., Bossuyt, P. M., & Van Den Bos, G. A. M. (1997). A stroke-adapted 30-item version of the Sickness Impact Profile to assess quality of life (SA-SIP30). *Stroke,**28*(11), 2155–2161. 10.1161/01.STR.28.11.2155 9368557 10.1161/01.str.28.11.2155

[CR161] Verhoeven, C. L., Post, M. W., Schiemanck, S. K., van Zandvoort, M. J., Vrancken, P. H., & van Heugten, C. M. (2011a). Is cognitive functioning 1 year poststroke related to quality of life domain? *Journal of Stroke & Cerebrovascular Diseases,**20*(5), 450–458. 10.1016/j.jstrokecerebrovasdis.2010.02.018 20813551 10.1016/j.jstrokecerebrovasdis.2010.02.018

[CR162] Verhoeven, C. L., Schepers, V. P., Post, M. W., & van Heugten, C. M. (2011b). The predictive value of cognitive impairments measured at the start of clinical rehabilitation for health status 1 year and 3 years poststroke. *International Journal of Rehabilitation Research,**34*(1), 38–43. 10.1097/MRR.0b013e32833ba577 20523223 10.1097/MRR.0b013e32833ba577

[CR163] Vilkki, J., Juvela, S., Malmivaara, K., Siironen, J., & Hernesniemi, J. (2012). Predictors of work status and quality of life 9–13 years after aneurysmal subarachnoid hemorrahage. *Acta Neurochirurgica (wien),**154*(8), 1437–1446. 10.1007/s00701-012-1417-y 10.1007/s00701-012-1417-y 22736050

[CR164] Vilkki, J., Servo, A., & Surma-aho, O. (1998). Word list learning and prediction of recall after frontal lobe lesions. *Neuropsychology,**12*(2), 268–277. 10.1037/0894-4105.12.2.268 9556773 10.1037//0894-4105.12.2.268

[CR165] Vincent, C., Desrosiers, J., Landreville, P., & Demers, L. (2009). Burden of caregivers of people with stroke: Evolution and predictors. *Cerebrovascular Diseases,**27*(5), 456–464. 10.1159/000210092 19329849 10.1159/000210092

[CR166] Visser-Meily, A., Post, M., Riphagen, I. I., & Lindeman, E. (2004). Measures used to assess burden among caregivers of stroke patients: A review. *Clinical Rehabilitation,**18*(6), 601–623. 10.1191/0269215504cr776oa 15473113 10.1191/0269215504cr776oa

[CR167] Visser-Meily, A., Post, M., Schepers, V., & Lindeman, E. (2005). Spouses’ quality of life 1 year after stroke: Prediction at the start of clinical rehabilitation. *Cerebrovascular Diseases,**20*(6), 443–448. 10.1159/000088983 16230849 10.1159/000088983

[CR168] von Steinbuechel, N., Richter, S., Morawetz, C., & Riemsma, R. (2005). Assessment of subjective health and health-related quality of life in persons with acquired or degenerative brain injury. *Current Opinion in Neurology,**18*(6), 681–691. 16280680 10.1097/01.wco.0000194140.56429.75

[CR169] Ware, J., Jr., & Sherbourne, C. D. (1992). The MOS 36-item short-form health survey (SF-36): I. Conceptual framework and item selection. *Medical Care,**30*(6), 473–83. 1593914

[CR170] Ware, J., Jr., Kosinski, M., Turner-Bowker, D. M., & Gandek, B. (2002). *How to score version 2 of the SF-12 Health Survey.* QualityMetric Incorporated.: Johnston RI USA.

[CR171] Ware, J., Jr., Kosinski, M., & Keller, S. D. (1996). A 12-Item Short-Form Health Survey: Construction of scales and preliminary tests of reliability and validity. *Medical Care,**34*(3), 220–233. 10.1097/00005650-199603000-00003 8628042 10.1097/00005650-199603000-00003

[CR172] Watson, P. A., Gignac, G. E., Weinborn, M., Green, S., & Pestell, C. (2020). A meta-analysis of neuropsychological predictors of outcome following stroke and other non-traumatic acquired brain injuries in adults. *Neuropsychology Review,**30*(2), 194–223. 10.1007/s11065-020-09433-9 32198606 10.1007/s11065-020-09433-9

[CR173] Wechsler, D. (1945). *Wechsler memory scale*. Psychological Corporation: San Antonio TX USA.

[CR174] Wechsler, D. (1955). *Wechsler adult intelligence scale*: Psychological Corporation. San Antonio TX USA.

[CR175] WHO. (2020). The top 10 causes of death. Retrieved from https://www.who.int/news-room/fact-sheets/detail/the-top-10-causes-of-death

[CR176] Williams, L. S., Weinberger, M., Harris, L. E., Clark, D. O., & Biller, J. (1999). Development of a stroke-specific quality of life scale. *Stroke,**30*(7), 1362–1369. 10.1161/01.STR.30.7.1362 10390308 10.1161/01.str.30.7.1362

[CR177] Wilson, B., Cockburn, J., & Baddeley, A. (1985). *The Rivermead Behavioural Memory Test.* Thames Valley Test Company: Bury St Edmunds UK.

[CR178] Wilson, B., Cockburn, J., Baddeley, A., & Hiorns, R. (1989). The development and validation of a test battery for detecting and monitoring everyday memory problems. *Journal of Clinical and Experimental Neuropsychology,**11*(6), 855–870. 10.1080/01688638908400940 2592527 10.1080/01688638908400940

[CR179] Wilson, B., Cockburn, J., & Halligan, P. (1987). Development of a behavioral test of visuospatial neglect. *Archives of Physical Medicine and Rehabilitation,**68*(2), 98–102. 3813864

[CR180] Wong, G. K. C., Lam, S. W., Ngai, K., Wong, A., Poon, W. S., & Mok, V. (2013). Development of a short form of Stroke-Specific Quality of Life Scale for patients after aneurysmal subarachnoid hemorrhage. *Journal of the Neurological Sciences,**335*(1), 204–209. 10.1016/j.jns.2013.09.033 24120271 10.1016/j.jns.2013.09.033

[CR181] Wu, C. Y., Skidmore, E. R., & Rodakowski, J. (2019). Relationship consensus and caregiver burden in adults with cognitive impairments 6 months following stroke. *PM&R,**11*(6), 597–603. 10.1002/pmrj.12009 30844137 10.1002/pmrj.12009 PMC6541546

[CR182] Zarit, S., Orr, N. K., & Zarit, J. M. (1985). *The hidden victims of Alzheimer’s disease: Families under stress*. NYU Press: New York USA.

[CR183] Zhu, W., & Jiang, Y. (2018). A meta-analytic study of predictors for informal caregiver burden in patients with stroke. *Journal of Stroke and Cerebrovascular Diseases,**27*(12), 3636–3646. 10.1016/j.jstrokecerebrovasdis.2018.08.037 30268368 10.1016/j.jstrokecerebrovasdis.2018.08.037

