# Supplementary material for: Post-stroke Cognition is Associated with Stroke Survivor Quality of Life and Caregiver Outcomes: A Systematic Review and Meta-analysis
Source: Neuropsychol Rev. 2024 Mar 11;34(4):1235–64. doi: 10.1007/s11065-024-09635-5 (PMC11607063; doi:10.1007/s11065-024-09635-5)
Supplement: Supplementary file 1 — Supplementary file1 (DOCX 429 KB) [file 11065_2024_9635_MOESM1_ESM.docx]

**SUPPLEMENTAL MATERIAL**

Post-Stroke Cognition is Associated with Stroke Survivor Quality of Life and Caregiver Outcomes: A Systematic Review and Meta-analysis

Renerus J. Stolwyk (DPsych ClinNeuro)^1^, Tijana Mihaljcic (DPsych ClinNeuro)^1^; Dana K. Wong (PhD)^2^, Diana Ramirez Hernandez (PhD)^1^, Brittany Wolff (MPsych ClinNeuro, PhD)^3,^ Jeffrey M. Rogers (PhD)^4^

^1^ School of Psychological Sciences and Turner Institute for Brain and Mental Health, Monash University, Melbourne, Australia.

^2^ School of Psychology & Public Health, La Trobe University, Bundoora, Australia.

^3^ School of Psychological Science, The University of Western Australia, Perth, Australia.

^4^ neuroCare Group, Sydney, Australia.

**Corresponding Author:** Associate Professor Renerus J. Stolwyk, Address: 18 Innovation Walk, Monash Clayton Campus, VIC 3800. Email: rene.stolwyk@monash.edu.

**Database Searches**

**Ovid MEDLINE**

1. cerebrovascular disorders/ or exp basal ganglia cerebrovascular disease/ or exp brain ischemia/ or exp carotid artery diseases/ or exp intracranial arterial diseases/ or exp "intracranial embolism and thrombosis"/ or exp intracranial hemorrhages/ or intracranial hemorrhage, traumatic/ or exp subarachnoid hemorrhage/ or exp stroke/ or exp brain infarction/ or exp stroke, lacunar/

2. (stroke or poststroke or post-stroke or cerebrovasc$ or brain vasc$ or cerebral vasc$ or CVA$ or apoplex$ or SAH).tw.

3. ((brain$ or cerebr$ or cerebell$ or intracran$ or intracerebral or MCA or anterior circulation or posterior circulation or basal ganglia) adj3 (isch?emi$ or infarct$ or thrombo$ or emboli$ or occuls$)).tw.

4. ((brain$ or cerebr$ or cerebell$ or intracran$ or intracerebral or subarachnoid or basal ganglia) adj3 (haemorrhage$ or hemorrhage$ or haematoma$ or hematoma$ or bleed$)).tw.

5. 1 or 2 or 3 or 4

6. exp cognition/ or exp executive function/ or exp memory/ or exp problem solving/ or exp verbal learning/ or exp perception/ or exp visual perception/ or exp decision making/ or exp judgment/

7. exp Attention/

8. exp cognition disorders/ or exp cognitive dysfunction/

9. exp Neuropsychology/

10. ((cognit$ or executive function$ or attention$ or concentrat$ or memory or percept$ or awareness or insight or speed or learning or recall or initiati$ or self-monitoring or self-awareness or organi?ation$ or neuropsych$) adj3 (disorder$ or dysfunct$ or impair$ or deficit$ or abilit$ or difficult$ or problem$ or disturbance$ or disabilit$ or declin$ or profile or reduce*)).tw.

11. 6 or 7 or 8 or 9 or 10

12. exp "Quality of Life"/

13. "Activities of Daily Living"/

14. Institutionalization/

15. Mortality/

16. follow-up studies/ or longitudinal studies/ or prospective studies/

17. Caregivers/

18. "caregiver burden".tw.

19. ((function$ or Activit$) adj3 (outcome$ or participation or independence or level)).tw.

20. ((Activit$ or participation) adj3 (limitation or restriction)).tw.

21. exp DEMENTIA/ or DEMENTIA, VASCULAR/ or DEMENTIA, MULTI-INFARCT

22. ((vascular or Alzhiemer$ or cerebr$) adj3 (dementia or impair$ or decline or disease or deteriorat$)).tw.

23. 12 or 13 or 14 or 15 or 16 or 17 or 18 or 19 or 20 or 21 or 22

24. 5 and 11 and 23

25. limit 24 to (english language and humans)

26. (pediatric* or child* or infant$ or sleep or apn?ea or diabetes or mellitus or atrial filtration or atrial fibrillation or heart failure or cardiovascular or congestive heart failure or aortic valve or cardiac or DNA or HIV or amyloid or toxicity or Parkinson$ disease or Lewy body or frontotemporal or hypertens$ or blood pressure or seizures or epilepsy or cancer or surgery or delirium or exercise or drug or pharmacologic$ or memantine or alcohol or animal or mice or mouse or antioxidants or milk or plasma or postmenopausal or serum or surgical or mutation or acid or extracellular or ganglioside or schizophrenia).tw.

27. 25 not 26

** Subsequent searches (April 2020, March 2021 and May 2023) included only outcomes of interest. Therefore, items 13, 14, 15, 19, 20, 21 and 22 were not included in the updated search.*

**Ovid PsycINFO**

1.cerebrovascular disorders/ or cerebral hemorrhage/ or exp cerebral ischemia/ or cerebrovascular accidents/ or subarachnoid hemorrhage/

2. (stroke or poststroke or post-stroke or cerebrovasc$ or brain vasc$ or cerebral vasc$ or CVA$ or apoplex$ or SAH).tw.

3. ((brain$ or cerebr$ or cerebell$ or intracran$ or intracerebral or MCA or anterior circulation or posterior circulation or basal ganglia) adj3 (isch?emi$ or infarct$ or thrombo$ or emboli$ or occuls$)).tw.

4. ((brain$ or cerebr$ or cerebell$ or intracran$ or intracerebral or subarachnoid or basal ganglia) adj3 (haemorrhage$ or hemorrhage$ or haematoma$ or hematoma$ or bleed$)).tw.

5. 1 or 2 or 3 or 4

6. cognitive impairment/ or cognition/ or cognitive ability/ or dysexecutive syndrome/ or memory disorders/

7. exp neuropsychological assessment/ or exp neuropsychological rehabilitation/ or exp neuropsychology/

8. exp attention/ or attention span/ or concentration/ or perception/

9. exp Executive Function/

10. exp memory/ or metacognition/

11. exp visuospatial ability/ or spatial ability/ or visual perception/ or visuospatial memory/

12. ((cognit$ or executive function$ or attention$ or concentrat$ or memory or percept$ or awareness or insight or speed or learning or recall or initiati$ or self-monitoring or self-awareness or organi?ation$ or neuropsych$) adj3 (disorder$ or dysfunct$ or impair$ or deficit$ or abilit$ or difficult$ or problem$ or disturbance$ or disabilit$ or declin$ or profile or reduce*)).tw.

13. 6 or 7 or 8 or 9 or 10 or 11 or 12

14. exp "Quality of Life"/

15. "activities of daily living"/ or ability level/ or activity level/ or daily activities/ or physical mobility

16. institutionalization/

17. "death and dying"/

18. followup studies/ or longitudinal studies/

19. Caregiver Burden/

20. ((function$ or Activit$) adj3 (outcome$ or participation or independence or level)).tw.

21. ((Activit$ or participation) adj3 (limitation or restriction)).tw.

22. exp DEMENTIA/ or VASCULAR DEMENTIA/

23. ((vascular or Alzhiemer$ or cerebr$) adj3 (dementia or impair$ or decline or disease or deteriorat$)).tw.

24. 14 or 15 or 16 or 17 or 18 or 19 or 20 or 21 or 22 or 23

25. 5 and 13 and 24

26. limit 25 to (human and english language)

27. (pediatric* or child* or infant$ or sleep or apn?ea or diabetes or mellitus or atrial filtration or atrial fibrillation or heart failure or cardiovascular or congestive heart failure or aortic valve or cardiac or DNA or HIV or amyloid or toxicity or Parkinson$ disease or Lewy body or frontotemporal or hypertens$ or blood pressure or seizures or epilepsy or cancer or surgery or delirium or exercise or drug or pharmacologic$ or memantine or alcohol or animal or mice or mouse or antioxidants or milk or plasma or postmenopausal or serum or surgical or mutation or acid or extracellular or ganglioside or schizophrenia).tw.

28. 26 not 27

** Subsequent searches (April 2020, March 2021 and May 2023) included only outcomes of interest. Therefore, items 15, 16, 17, 20, 21, 22 and 23 were not included in the updated search.*

**EBSCOhost CINAHL Plus**

1. (MH "Stroke+") OR (MH "Intracranial Hemorrhage+") OR (MH "Cerebral Hemorrhage+") OR (MH "Cerebrovascular Disorders+") OR (MH "Cerebral Ischemia+") OR (MH "Basal Ganglia Cerebrovascular Disease+") OR (MH "Intracranial Embolism and Thrombosis+")

2. stroke or poststroke or post-stroke or cerebrovasc* or "brain vasc*" or "cerebral vasc*" or CVA* or apoplex* or SAH

3. brain* or cerebr* or cerebell* or intracran* or intracerebral or MCA or "anterior circulation" or "posterior circulation" or "basal ganglia"

4. isch?emi* or infarct* or thrombo* or emboli* or occuls*****

5. S3 N3 S4

6. brain* or cerebr* or cerebell* or intracran* or intracerebral or subarachnoid or "basal ganglia"

7. haemorrhage* or hemorrhage* or haematoma* or hematoma* or bleed*

8. S6 N3 S7

9. S1 OR S2 OR S5 OR S8

10. (MH "Cognition") OR (MH "Executive Function") OR (MH "Learning") OR (MH "Perception") OR (MH "Thinking") OR (MH "Decision Making") OR (MH "Judgment") OR (MH "Problem Solving") OR (MH "Memory")

11. (MH "Neuropsychology")

12. (MH "Cognition Disorders")

13. (MH "Attention")

14. (MH "Visual Perception") OR (MH "Spatial Perception")

15. cognit* or “executive function*” or attention* or concentrat* or memory or percept* or awareness or insight or speed or learning or recall or initiati* or self-monitoring or self-awareness or organi?ation* or neuropsych*

16. disorder* or dysfunct* or impair* or deficit* or abilit* or difficult* or problem* or disturbance* or disabilit* or declin* or profile or reduce*

17. S15 N3 S16

18. S10 OR S11 OR S12 OR S13 OR S14 OR S17

19. (MH "Quality of Life+")

20. (MH "Activities of Daily Living")

21. (MH "Institutionalization")

22. (MH "Mortality")

23. (MH "Prospective Studies+")

24. (MH "Caregiver Burden")

25. function* or Activit*

26. outcome* or participation or independence or level

27. S25 N3 S26

28. Activit* or participation*

29. limitation or restriction

30. S28 N3 S29

31. (MH "Dementia+") OR (MH "Dementia, Vascular+") OR (MH "Dementia, Multi-Infarct")

32. vascular Alzhiemer* or cerebr*

33. dementia or impair* or decline or deteriorat*

34. S32 N3 S33

35. S19 OR S20 OR S21 OR S22 OR S23 OR S24 OR S27 OR S30 OR S31 OR S34

36. S9 AND S18 AND S35

37. pediatric* or child* or infant# or sleep or apn#ea or diabetes or mellitus or "atrial filtration" or "atrial fibrillation" or "heart failure" or cardiovascular or "congestive heart failure" or "aortic valve" or cardiac or DNA or HIV or amyloid or toxicity or "Parkinson# disease" or "Lewy body" or frontotemporal or hypertens$ or "blood pressure" or seizures or epilepsy or cancer or surgery or delirium or exercise or drug or pharmacologic* or memantine or alcohol or animal or mice or mouse or antioxidants or milk or plasma or postmenopausal or serum or surgical or mutation or acid or extracellular or ganglioside or schizophrenia

38. S36 NOT S37

** Subsequent searches (April 2020, March 2021 and May 2023) included only outcomes of interest. Therefore, items 20, 21, 22, 25, 26, 28, 29, 21, 32, 33 were not included in the updated search.*

**Ovid Embase**

1. exp cerebrovascular accident/ or cerebrovascular disease/ or lacunar stroke/

2. (stroke or poststroke or post-stroke or cerebrovasc$ or brain vasc$ or cerebral vasc$ or CVA$ or apoplex$ or SAH).tw.

3. ((brain$ or cerebr$ or cerebell$ or intracran$ or intracerebral or MCA or anterior circulation or posterior circulation or basal ganglia) adj3 (isch?emi$ or infarct$ or thrombo$ or emboli$ or occuls$)).tw.

4. ((brain$ or cerebr$ or cerebell$ or intracran$ or intracerebral or subarachnoid or basal ganglia) adj3 (haemorrhage$ or hemorrhage$ or haematoma$ or hematoma$ or bleed$)).tw.

5. 1 or 2 or 3 or 4

6. exp cognition/ or exp executive function/ or exp learning/ or exp memory/ or exp metacognition/ or exp perception/

7. exp neuropsychological test/ or exp neuropsychology/

8. exp attention/

9. exp auditory memory/ or exp prospective memory/ or exp recall/ or exp recognition/ or exp short term memory/ or exp verbal memory/ or exp visual memory/ or exp working memory/

10. ((cognit$ or executive function$ or attention$ or concentrat$ or memory or percept$ or awareness or insight or speed or learning or recall or initiati$ or self-monitoring or self-awareness or organi?ation$ or neuropsych$) adj3 (disorder$ or dysfunct$ or impair$ or deficit$ or abilit$ or difficult$ or problem$ or disturbance$ or disabilit$ or declin$ or profile or reduce*)).tw.

11. 6 or 7 or 8 or 9 or 10

12. exp "quality of life"/ or exp "quality of life index"/

13. exp daily life activity/

14. exp institutionalization/ or exp institutional care/

15. exp mortality rate/ or exp mortality risk/

16. exp caregiver burden/

17. ((function$ or Activit$) adj3 (outcome$ or participation or independence or level)).tw.

18. ((Activit$ or participation) adj3 (limitation or restriction)).tw.

19. exp dementia/ or alzheimer disease/ or multiinfarct dementia/

20. ((vascular or Alzhiemer$ or cerebr$) adj3 (dementia or impair$ or decline or deteriorat$)).tw.

21. 12 or 13 or 14 or 15 or 16 or 17 or 18 or 19 or 20

22. 5 and 11 and 21

23. limit 22 to (english language and humans)

24. (pediatric* or child* or infant$ or sleep or apn?ea or diabetes or mellitus or atrial filtration or atrial fibrillation or heart failure or cardiovascular or congestive heart failure or aortic valve or cardiac or DNA or HIV or amyloid or toxicity or Parkinson$ disease or Lewy body or frontotemporal or hypertens$ or blood pressure or seizures or epilepsy or cancer or surgery or delirium or exercise or drug or pharmacologic$ or memantine or alcohol or animal or mice or mouse or antioxidants or milk or plasma or postmenopausal or serum or surgical or mutation or acid or extracellular or ganglioside or schizophrenia).tw.

25. 23 not 24

** Subsequent searches (April 2020, March 2021 and May 2023) included only outcomes of interest. Therefore, items 13, 14, 15, 17, 18 , 19 and 20 were not included in the updated search.*

**Elsevier Scopus**

1. TITLE-ABS-KEY ( ( stroke OR "cerebrovascular disorders" OR "cerebrovascular accident" OR cva OR sah ) )

2. TITLE-ABS-KEY ( ( ( brain* OR cerebr* OR cerebell* OR intracran* OR intracerebral OR mca OR "anterior circulation" OR "posterior circulation" OR "basal ganglia" ) W/3 ( isch*emi* OR infarct* OR thrombo* OR emboli* OR occuls* ) ) )

3. TITLE-ABS-KEY ( ( ( brain* OR cerebr* OR cerebell* OR intracran* OR intracerebral OR subarachnoid OR "basal ganglia" ) W/3 ( haemorrhage* OR hemorrhage* OR haematoma* OR hematoma* OR bleed* ) ) )

4. #1 OR #2 OR #3

5. TITLE-ABS-KEY ( ( ( cognit* OR "executive function*" OR attention* OR concentrat* ) W/3 ( disorder* OR dysfunct* OR impair* OR deficit* OR abilit* OR difficult* OR problem* OR disturbance* OR disabilit* OR declin* OR profile OR reduce* ) ) )

6. TITLE-ABS-KEY ( ( ( memory OR percept* OR awareness OR insight OR speed ) W/3 ( disorder* OR dysfunct* OR impair* OR deficit* OR abilit* OR difficult* OR problem* OR disturbance* OR disabilit* OR declin* OR profile OR reduce* ) ) )

7. TITLE-ABS-KEY ( ( ( learning OR recall OR initiati* OR self-monitoring ) W/3 ( disorder* OR dysfunct* OR impair* OR deficit* OR abilit* OR difficult* OR problem* OR disturbance* OR disabilit* OR declin* OR profile OR reduce* ) ) )

8. TITLE-ABS-KEY ( ( ( self-awareness OR organi?at OR neuropsych* ) W/3 ( disorder* OR dysfunct* OR impair* OR deficit* OR abilit* OR difficult* OR problem* OR disturbance* OR disabilit* OR declin* OR profile OR reduce* ) ) )

9. #5 OR #6 OR #7 OR #8

10. TITLE-ABS-KEY ( ( ( {quality of life} OR {activities of daily living} OR institutionalisation OR mortality OR "caregiver burden" ) ) )

11. TITLE-ABS-KEY ( ( ( function* OR activit* ) W/3 ( outcome* OR participation OR independence OR level ) ) )

12. TITLE-ABS-KEY ( ( ( activit* OR participation* ) W/3 ( limitation OR restriction ) ) )

13. TITLE-ABS-KEY ( ( ( vascular OR alzhiemer* OR cerebr* ) W/3 ( dementia OR impair* OR decline OR deteriorat* ) ) )

14. #10 OR #11 OR #12 OR #13

15. #4 AND #9 AND # 14

16. TITLE-ABS-KEY ( pediatric* OR child* OR infant* OR sleep OR apn*ea OR diabetes OR mellitus OR "atrial fibrillation" OR "heart failure" OR cardiovascular OR "congestive heart failure" OR "aortic valve" OR cardiac OR pharmacologic* OR memantine OR alcohol OR animal OR mice OR mouse OR rodent OR dna OR hiv OR amyloid OR toxicity OR "Parkinson* disease" OR "Lewy body" OR frontotemporal OR hypertens* OR "blood pressure" OR seizures OR epilepsy OR cancer OR surgery OR delirium OR exercise or drug or antioxidants or milk or plasma or postmenopausal or serum or surgical or mutation or acid or extracellular or ganglioside or schizophrenia)

17. #15 AND NOT #16

18. #17 (limit to English)

** Subsequent searches (April 2020, March 2021 and Mar 2023) included only outcomes of interest. Therefore, items 11, 12 and 13 were not included in the updated search.*

**QUIPS Tool**

| **QUIPS Original** | **Modified QUIPS Criteria for RoB Ratings in current meta-analysis** | | | | | |
| --- | --- | --- | --- | --- | --- | --- |
| **Potential Bias** |  |  |  |  |  |  |
| **Study Participation**  The study sample represents the population of interest on key characteristics sufficient to limit potential bias to the results. | **Yes response** |  | | | | |
|  | Prospective cohort of stroke survivors  AND key characteristics provided (i.e. demographics, time since stroke)  AND inclusion/exclusion criteria provided.  The sampling frame and recruitment adequately described (e.g. period of recruitment, place of recruitment, how sample was identified)  **Retrospective studies score no better than “moderate bias” for study participation** |  | | | | |
|  |  | YES | PARTLY | | NO/UNSURE | |
| **Study Attrition**  Loss to follow-up (from sample to study population) is not associated with key characteristics (i.e., the study data adequately represent the sample), sufficient to limit potential bias. | **Yes response** | **Mark down if:** | | | | |
|  | Number seen at assessment: >80% (up to 5 years) or >70% (over 5 years)  AND reasons lost to follow-up reported with numbers  AND comparison of lost versus not lost to follow-up with no important differences if response rate OR if importance differences found addressed in the analysis.  **OR (non-prognostic studies)**  Time period for recruiting participants reported  Indicate if subjects were consecutive if not population based | Number seen at assessment: <65% (up to 5 years) or <50% (over 5 years).  Attrition/denominators not reported.  Key characteristics not compared between those followed up and those not followed up  **No if 2 or more of the above**  **OR (non-prognostic studies)**  No mention of recruitment period  No mention if recruitment is consecutive | | | | |
|  |  | YES | PARTLY | | NO/UNSURE | |
| **Prognostic factor measurement**  The prognostic factor of interest is adequately measured in participants to sufficiently limit potential bias | **Yes response**  Clear description of the neuropsychological or screening measures used  AND used in the intended way (i.e. not aggregate scores, combined with language)  AND method of measurement is a validated scale  AND continuous variables left as continuous or rational provided for cut-offs | **Mark down if:**  Definition of cognition is not clear  OR use of non-validated scales  OR aggregates used so it’s difficult to determine which cognitive domains are impaired | | | | |
|  |  |  | | | | |
|  |  | YES | | PARTLY | | NO/UNSURE |
| **Outcome Measurement**  The outcome of interest is adequately measured in study participants to sufficiently limit potential bias. | **Yes response** | **Mark down if:** | | | | |
|  | Clear description and definition of the outcomes provided which may include validated scales and/or well-defined outcomes  AND continuous variables left as continuous or rationale provided for cut-offs | The outcomes are not appropriately defined  Rationale provided for the cut-offs is inadequate  Overall outcome is not significant and subsection scores are explored (i.e. no rationale for this in the introduction)  **No if 2 or more of the above** | | | | |
|  |  | YES | | PARTLY | | NO/UNSURE |
| **Confounding Measurement and Account**  Important potential confounders are appropriately accounted for, limiting potential bias with respect to the prognostic factor of interest. | **Yes response** | **Mark down if:** | | | | |
|  | List of stroke survivor factors includes 1) age, 2) gender, 3) education or SES, 4) stroke severity, 5) physical limitations, 6) psychological (i.e. depression) and 7) treatment factors.  Another rationale for looking into confounders (e.g. all significant relationships entered) is acceptable. | One or more of these factors is not considered (unless those factors are 6 or 7)  No = None of these factors are considered as candidate factors, or if they are considered, are eliminated without statistical testing. | | | | |
|  |  | YES | | PARTLY | | NO/UNSURE |
| **Analysis**  The statistical analysis is appropriate for the design of the study, limiting potential for presentation of invalid results. | **Yes response** | **Mark down if:** | | | | |
|  | Statistical model used appropriate for the study design and type of data  AND a model building strategy used, e.g. stepwise, forward, backward selection  AND strategy and results clearly reported  AND completeness of reporting of results in final multivariable model  If additional data was provided by authors on request this was considered in the rating. | Statistical model not appropriate  Unclear reporting of strategy or results  Selective reporting of results.  NO if 2 or more of the above. | | | | |
|  |  | YES | | PARTLY | | NO/UNSURE |

**Supplementary Table 1:** Near miss studies

| **Author (Year)** | **Measures** | **Findings** | **Reason not included** |
| --- | --- | --- | --- |
| **Quality of Life** |  |  |  |
| Abdullah, Sharip, Rahman, and Bakar (2021) | Screening (MoCA)  WHO-QOL-BREF-21 | MoCA did not significantly correlate with QoL | Authors replied. Follow up did not meet criteria. Participants were between 1-108 months post-stroke |
| Aliyu, Ibrahim, Saidu, and Owolabi (2018) | Screening (modified MMSE)  HRQoLISP-40 | Binary logistic regression and odds ratio showed that the presence of cortical lesion, aphasia, cognitive impairment, and post-stroke depression (PSD) were predictors of poor global HRQoL. Independent predictors of poor global HRQoL were aphasia, lesion location, and PSD. | Unclear follow up (at least 1 month post stroke). Author emailed but data not available/no response. |
| Alvarez-Sabín et al. (2016) | Neuropsychological assessment categorised as global cognitive impairment vs no cognitive impairment  Euro-QoL 5D | Patients with cognitive impairment had a poorer QoL at 2 years. | Ineligible study design investigating effect of citicoline treatment in QoL and cognitive performance in long term stroke survivors. Unable to extract data. |
| Canuto, Nogueira, and Araújo (2016) | Screening (MMSE)  Stroke Specific Quality of Life Scale (SS-QOL) | MMSE was significantly correlated with SS-QoL (r = 0.4) | Unclear if follow up period met criteria. Authors emailed but no response. |
| Carod-Artal, Stieven Trizotto, Ferreira Coral, and Menezes Moreira (2009) | Screening (MMSE)  Stroke Impact Scale (SIS) | SIS Communication significantly correlated with MMSE (r =0.42), but no other domains reported as correlations. Linear regression with significant predictors presented. | Unable to extract data (stepwise linear regression or missing data). Unclear if follow up period met criteria = 20.7 months (SD =24.8) Authors emailed to clarify with no response. |
| Cawood, Visagie, and Mji (2016) | LOTCA Stroke Impact Scale (SIS) | SIS Participation significantly correlated with LOTCA thinking operations, visuomotor organisation and perception (r = 0.50-0.61). | Ineligible measure of QoL. Included only the SIS Participation subscale. |
| Chen et al. (2015) | Screening (MMSE)  Stroke Impact Scale (SIS) | MMSE was significantly correlated with SIS total (r = 0.56). | Unclear if follow up period met criteria = 6.04 months (SD = 4.17). Authors emailed to clarify with no response |
| Chou (2015) | Screening (MMSE)  Stroke-Specific Quality of Life (SS-QoL) | MMSE independently predicted SS-QoL total score (p = .023) in multiple hierarchical regression with stepwise analysis. | No bivariate relationship reported. Unable to extract data. Author emailed but address inactive. |
| Cumming, Brodtmann, Darby, and Bernhardt (2014) | Neuropsychological measures  Assessment of Quality of Life (AQoL) | Multivariate regression, greater degree of cognitive impairment at 3 months was significantly associated with lower AQoL score at 12 months (p = .021). Attention  and visuospatial ability were significantly associated with 12-month AQoL score. Significance was not reached for executive function, language or memory. | No bivariate relationship reported. Unable to extract data. Author emailed but address inactive, other address attempted but no response. |
| Dhamoon et al. (2010) | Screening (MMSE)  Spitzer QOL index (QLI) | QLI declined annually up to 5 years after stroke and was associated with cognition. | Decline in QoL over time so did not meet inclusion criteria. |
| Fatoye et al. (2007) | Screening (MMSE)  WHO-QOL-BREF | Modified MMSE scores had significant positive correlations with scores on all the six aspects of the WHOQOL-BREF | Unclear if follow up period met criteria. Unable to extract data on cognition and QoL relationship. Authors emailed with no response (address not active). |
| Franceschini, La Porta, Agosti, and Massucci (2010) | Screening (MMSE) and neglect (not a validated measure)  EuroQoL-5D and Visual Analogue Score (QoL-VAS) | A lower MMSE and the presence of neglect were highly associated with HRQOL one year after stroke | Unable to extract data. Authors emailed with no response. |
| Haacke et al. (2006) | Screening (MMSE)  EuroQoL Index (EQ-5D) and the Health Utility Index 2 and 3 | Four years after stroke, cognitive impairment contributed to a reduced HRQoL. | TIA was part of the sample (23%). Unable to extract stroke specific data. Authors emailed with no response. |
| Jonsson, Lindgren, HallstrÖM, Norrving, and Lindgren (2005) | Screening (MMSE)  SF-36 | For patient, cognition was only significantly associated with Bodily Pain. | No bivariate relationship reported, regression only. Unable to extract data. Author emailed but address inactive. |
| Kamel, Ghani, Zaiton, El-Motayam, and El-Fattah (2010) | Screening (MMSE)  Stroke Impact Scale (SIS) | MMSE significantly associated with SIS memory and communications (r = 0.43- 0.47), but not SIS physical, emotional or participation (r = 0.09-0.15). | Unclear when SIS was measured to extract correlations. Authors contacted with no response. |
| Mayer et al. (2002) | Neuropsychological assessment and screening (TICS)  Sickness Impact Profile (SIP) | TICS significant relationship with SIP total. Motor function and reaction time showed a significant association with SIP total, but visual memory, visuospatial function, verbal memory, language, and executive function had no relationship. | Relationship between cognitive impairment and outcome adjusted for age, education and race/ethnicity so unable to extract data. Authors emailed with no response. |
| Nys et al. (2006) | Neuropsychological assessment  SS-QOL | Early cognitive impairment was a predictor of QoL after the first 6 months in univariate analyses. | Regression data available only so unable to extract. Authors emailed with no response. |
| Özturk, Akyol, Ulus, Tander, and Kuru (2018) | Screening (MMSE)  Stroke Impact Scale (SIS) | MMSE was significantly correlated with SIS total (r = 0.584) | Unclear if follow up period met criteria (1 -70 months). Authors contacted to clarify with no response. |
| Peixoto et al. (2017) | Screening (MoCA)  Assessment scale of quality of life in stroke (ECVI-38) | MoCA was significantly correlated with ECVI-38 total (r = -0.48) | Authors emailed. 33% of sample were less than 3 months post stroke therefore did not meet inclusion criteria. |
| Silva, Corrêa, Pereira, and Corrêa (2019) | Screening (MMSE)  SS-QOL | Cognitive status was significantly associated with SS-QOL in regression analysis | Regression data available only so unable to extract data. Authors emailed with no response. |
| Tse et al. (2017) | Screening (MoCA)  Stroke Impact Scale (SIS) | Dichotomised MoCA significantly associated with SIS Communication and Participation at 12 months. Not associated with SIS Physical, Memory and Thinking, Emotion, or Perceived Recovery. | Regression data available only so unable to extract. Authors emailed with no response. |
| van Zandvoort, Kessels, Nys, de Haan, and Kappelle (2005) | Neuropsychological Assessment  MOS - SF-36 and VAS | No significant relationship was found between cognitive domain scores and QoL by means of a linear regression. | Regression data available only so unable to extract. Authors emailed with no response (address not active). |
| Wong et al. (2012) | Screening (MMSE, MoCA)  SF-36 | MMSE and MoCA were not correlated with SF-36 physical health and mental health component scores. | Correlations not available. Authors emailed. Do not keep data beyond 7 years. Excluded as unable to extract data. |
| **Caregiver Outcomes** |  |  |  |
| Bugge, Alexander, and Hagen (1999) | Screening (MMSE)  Caregiver Strain Index and SF-36 | Unclear if cognition was entered in regression analysis (patient variables at stage 2). If assumed it was, cognition was not a significant predictor of caregiver strain | No bivariate relationship reported. Unable to extract data. Author emailed with no response. |
| Jonsson et al. (2005) | Screening (MMSE)  SF-36 (caregiver) | Cognition only significantly associated with Role Limitations (Physical), Bodily Pain and Physical Component Summary (PCS) | No bivariate relationship reported, regression only. Unable to extract data. Author emailed but address inactive. |
| Rigby et al. (2009) | Screening (Orientation and clock drawing)  Relatives Stress Scale (RSS) and Bakas Caregiver Outcomes Scale (BOCS) | Orientation and clock drawing significantly associated with RSS but not BCOS. | Sample included individuals with TIA (13.5%) Authors emailed with no response. Excluded because stroke only data was not available. |
| Smith et al. (2004) | Screening (Hodkinson Abbreviated Mental Test)  SF-36 General Health and Carers’  Assessment of Difficulties Index (CADI), Carers’ Assessment of Satisfaction (with caring) Index  (CASI), and the Carers’ Assessment of Managing Index (CAMI) | Cognition not reported as significant predictor of caregiver outcome | Unable to extract data. Authors emailed with no response. |
| Thommessen, Wyller, Bautz-Holter, and Laake (2001) | Screening (MMSE)  Relatives Stress Scale (RSS) | The patient’s cognitive function during the acute phase (MMSE) was the only variable significantly associated with a psychosocial burden 6 months after stroke in bivariate and multiple linear regression analyses | Regression data available only so unable to extract. Authors emailed with no response. |
| Thommessen et al. (2002) | Screening (MMSE)  Relative Stress Scale (RSS) | Lower cognitive function of the patient was associated with higher psychosocial burden on the spouse for patient with stroke. | Regression data available only so unable to extract. As per above, unable to contact authors. |

**References**

Abdullah, A. H., Sharip, S., Rahman, A. H. A., & Bakar, L. (2021). Cognitive reserve in stroke patients. *PsyCh Journal, 10*(3), 444-452. doi:10.1002/pchj.423

Aliyu, S., Ibrahim, A., Saidu, H., & Owolabi, L. (2018). Determinants of health-related quality of life in stroke survivors in Kano, Northwest Nigeria. *Journal of Medicine in the Tropics, 20*(1), 11-16. doi:10.4103/jomt.jomt_26_17

Alvarez-Sabín, J., Santamarina, E., Maisterra, O., Jacas, C., Molina, C., & Quintana, M. (2016). Long-term treatment with citicoline prevents cognitive decline and predicts a better quality of life after a first ischemic stroke. *International Journal of Molecular Sciences, 17*(3), 390-390. doi:10.3390/ijms17030390

Bugge, C., Alexander, H., & Hagen, S. (1999). Stroke patients' informal caregivers: Patient, caregiver, and service factors that affect caregiver strain. *Stroke, 30*(8), 1517-1523. doi:10.1161/01.STR.30.8.1517

Canuto, M. Â. d. O., Nogueira, L. T., & Araújo, T. M. E. d. (2016). Health-related quality of life after stroke. *Acta Paulista de Enfermagem, 29*(3), 245-252. doi:10.1590/1982-0194201600035

Carod-Artal, F. J., Stieven Trizotto, D., Ferreira Coral, L., & Menezes Moreira, C. (2009). Determinants of quality of life in Brazilian stroke survivors. *Journal of the Neurological Sciences, 284*(1), 63-68. doi:10.1016/j.jns.2009.04.008

Cawood, J., Visagie, S., & Mji, G. (2016). Impact of post-stroke impairments on activities and participation as experienced by stroke survivors in a Western Cape setting. *South African Journal of Occupational Therapy, 46*, 10-15. doi:10.17159/2310-3833/2016/v46n2a3

Chen, C.-M., Tsai, C.-C., Chung, C.-Y., Chen, C.-L., Wu, K. P., & Chen, H.-C. (2015). Potential predictors for health-related quality of life in stroke patients undergoing inpatient rehabilitation. *Health and Qualiity of Life Outcomes, 13*(1), 118-118. doi:10.1186/s12955-015-0314-5

Chou, C.-Y. M. A. (2015). Determinants of the health-related quality of life for stroke survivors. *Journal of Stroke & Cerebrovascular Diseases, 24*(3), 655-662. doi:10.1016/j.jstrokecerebrovasdis.2014.10.022

Cumming, T. B., Brodtmann, A., Darby, D., & Bernhardt, J. (2014). The importance of cognition to quality of life after stroke. *Journal of Psychosomatic Research, 77*(5), 374-379. doi:10.1016/j.jpsychores.2014.08.009

Dhamoon, M. S., Moon, Y. P., Paik, M. C., Boden-Albala, B., Rundek, T., Sacco, R. L., & Elkind, M. S. V. (2010). Quality of life declines after first ischemic stroke: The Northern Manhattan Study. *Neurology, 75*(4), 328-334. doi:10.1212/WNL.0b013e3181ea9f03

Fatoye, F. O., Komolafe, M. A., Eegunranti, B. A., Adewuya, A. O., Mosaku, S. K., & Fatoye, G. K. (2007). Cognitive impairment and quality of life among stroke survivors in Nigeria. *Psychological Reports, 100*(3), 876-882. doi:10.2466/pr0.100.3.876-882

Franceschini, M., La Porta, F., Agosti, M., & Massucci, M. (2010). Is health-related-quality of life of stroke patients influenced by neurological impairments at one year after stroke? *European Journal of Physical and Rehabilitation Medicine, 46*(3), 389-399.

Haacke, C., Althaus, A., Spottke, A., Siebert, U., Back, T., & Dodel, R. (2006). Long-term outcome after stroke. *Stroke, 37*(1), 193-198. doi:10.1161/01.STR.0000196990.69412.fb

Jonsson, A.-C., Lindgren, I., HallstrÖM, B., Norrving, B., & Lindgren, A. (2005). Determinants of quality of life in stroke survivors and their informal caregivers. *Stroke, 36*(4), 803-808. doi:10.1161/01.STR.0000160873.32791.20

Kamel, A., Ghani, A. A., Zaiton, M. A., El-Motayam, A. S., & El-Fattah, D. A. (2010). Health related quality of life in stroke survivors measured by the Stroke Impact Scale. *The Egyptian Journal of Neurology, Psychiatry and Neurosurgery, 47*, 267-274.

Mayer, S. A., Kreiter, K. T., Copeland, D., Bernardini, G. L., Bates, J. E., Peery, S., . . . Connolly, E. S. (2002). Global and domain-specific cognitive impairment and outcome after subarachnoid hemorrhage. *Neurology, 59*(11), 1750-1758. doi:10.1212/01.WNL.0000035748.91128.C2

Nys, G. M. S., van Zandvoort, M. J. E., van der Worp, H. B., de Haan, E. H. F., de Kort, P. L. M., Jansen, B. P. W., & Kappelle, L. J. (2006). Early cognitive impairment predicts long-term depressive symptoms and quality of life after stroke. *Journal of the Neurological Sciences, 247*(2), 149-156. doi:10.1016/j.jns.2006.04.005

Özturk, S., Akyol, Y., Ulus, Y., Tander, B., & Kuru, Ö. (2018). Determinants of disease specific health-related quality of life in stroke patients. *Journal of Physical Medicine & Rehabilitation Sciences/Fiziksel Tup ve Rehabilitasyon Bilimleri Dergisi, 21*(3), 107-114. doi:10.31609/jpmrs.2018-60048

Peixoto, B., Silva, S., Carreira, S., Sousa, D., Rezende, V., & Teixeira, A. (2017). Quality of life predictors after first stroke: A study with post-acute patients. *Neurology, Psychiatry, and Brain Research, 23*, 10-15. doi:10.1016/j.npbr.2016.11.003

Rigby, H., Gubitz, G., Eskes, G., Reidy, Y., Christian, C., Grover, V., & Phillips, S. (2009). Caring for stroke survivors: Baseline and 1-year determinants of caregiver burden. *International Journal of Stroke, 4*(3), 152-158. doi:10.1111/j.1747-4949.2009.00287.x

Silva, S. M., Corrêa, J. C. F., Pereira, G. S., & Corrêa, F. I. (2019). Social participation following a stroke: an assessment in accordance with the international classification of functioning, disability and health. *Disability & Rehabilitation, 41*(8), 879-886. doi:10.1080/09638288.2017.1413428

Smith, L. N., Norrie, J., Kerr, S. M., Lawrence, I. M., Langhorne, P., & Lees, K. R. (2004). Impact and influences on caregiver outcomes at one year post-stroke. *Cerebrovascular Diseases, 18*(2), 145-153. doi:10.1159/000079268

Thommessen, B., Aarsland, D., Braekhus, A., Oksengaard, A. R., Engedal, K., & Laake, K. (2002). The psychosocial burden on spouses of the elderly with stroke, dementia and Parkinson's disease. *International Journal of Geriatric Psychiatry, 17*(1), 78-84. doi:10.1002/gps.524

Thommessen, B., Wyller, T. B., Bautz-Holter, E., & Laake, K. (2001). Acute phase predictors of subsequent psychosocial burden in carers of elderly stroke oatients. *Cerebrovascular Diseases, 11*(3), 201-206. doi:10.1159/000047639

Tse, T., Binte Yusoff, S. Z., Churilov, L., Ma, H., Davis, S., Donnan, G. A., & Carey, L. M. (2017). Increased work and social engagement is associated with increased stroke specific quality of life in stroke survivors at 3 months and 12 months post-stroke: a longitudinal study of an Australian stroke cohort. *Topics in Stroke Rehabilitation, 24*(6), 405-414. doi:10.1080/10749357.2017.1318339

van Zandvoort, M. J. E., Kessels, R. P. C., Nys, G. M. S., de Haan, E. H. F., & Kappelle, L. J. (2005). Early neuropsychological evaluation in patients with ischaemic stroke provides valid information. *Clinical Neurology and Neurosurgery, 107*(5), 385-392. doi:10.1016/j.clineuro.2004.10.012

Wong, G. K. C., Lam, S., Ngai, K., Wong, A., Mok, V., & Poon, W. S. (2012). Evaluation of cognitive impairment by the Montreal Cognitive Assessment in patients with aneurysmal subarachnoid haemorrhage: prevalence, risk factors and correlations with 3 month outcomes. *Journal of Neurology, Neurosurgery & Psychiatry, 83*(11), 1112-1117. doi:10.1136/jnnp-2012-302217

**Supplementary Table 2: Moderator analysis of dichotomous factors**

|  |  |  | Effect Size Statistics (Random Effects Model) | | | Heterogeneity | | |
| --- | --- | --- | --- | --- | --- | --- | --- | --- |
|  |  |  | *k* | *r* (95% CI) | *p* | *I^2^* | *Q* | *p* |
| **COGNITION (ALL MEASURES COMBINED)** | **Stroke Survivor**  **QoL** | **Study Quality** |  |  |  |  |  |  |
|  |  | High/Moderate | 26 | 0.19 (0.14-0.24) | <0.01 | 89.96 | 246.46 | <0.01 |
|  |  | Low | 12 | 0.30 (0.23-0.37) | <0.01 | 65.71 | 32.08 | <0.01 |
|  |  | between-group difference |  |  |  |  | 6.21 | 0.013 |
|  |  | **Sequential vs Concurrent Measurement** |  |  |  |  |  |  |
|  |  | Sequential | 10 | 0.16 (0.08-0.24) | <0.01 | 92.02 | 112.79 | <0.01 |
|  |  | Concurrent | 28 | 0.25 (0. 20-0.30) | <0.01 | 71.49 | 94.73 | <0.01 |
|  |  | between-group difference |  |  |  |  | 3.74 | 0.053 |
|  | **Caregiver Outcomes (QoL and Burden)** | **Study Quality** |  |  |  |  |  |  |
|  |  | High/Moderate | 7 | 0.07 (-0.05-0.19) | <0.01 | 64.26 | 16.79 | 0.01 |
|  |  | Low | 8 | 0.22 (0.15-0.30) | <0.01 | 48.96 | 13.72 | 0.06 |
|  |  | between-group difference |  |  |  |  | 4.32 | 0.038 |
|  |  | **Sequential vs Concurrent Measurement** |  |  |  |  |  |  |
|  |  | Sequential | 4 | 0.10 (-0.04-0.23) | 0.17 | 58.44 | 7.22 | 0.07 |
|  |  | Concurrent | 11 | 0.19 (0.11-0.28) | <0.01 | 66.78 | 30.10 | <0.01 |
|  |  | between-group difference |  |  |  |  | 1.33 | 0.25 |

**Supplementary Table 3: Moderator analysis of continuous factors**

|  |  |  | N | Q | β | z | *p* | R^2^ |
| --- | --- | --- | --- | --- | --- | --- | --- | --- |
| **COGNITION (ALL MEASURES COMBINED)** | **Stroke Survivor**  **QoL** | **Sample Size** | 38 | 2.77 | -0.00 | -1.66 | 0.10 | 0.21 |
|  |  | **Age** | 34 | 0.46 | -0.00 | -0.68 | 0.50 | 0* |
|  |  | **Time Since Injury** | 37 | 0.01 | 0.00 | 0.07 | 0.94 | 0.02 |
|  | **Caregiver Outcomes (QoL and Burden)** | **Sample Size** | 15 | 0.01 | 0.00 | 0.10 | 0.92 | 0* |
|  |  | **Age** | 12 | 0.16 | 0.00 | 0.39 | 0.69 | 0* |
|  |  | **Caregiver Age** | 12 | 0.04 | 0.00 | 0.19 | 0.85 | 0* |
|  |  | **Time Since Injury** | 14 | 2.17 | 0.00 | 1.47 | 0.14 | 0.06 |

*Negative values are truncated to zero

**Funnel Plots – Stroke Survivor**

| **Overall Cognition** | **Screening** |
| --- | --- |
| 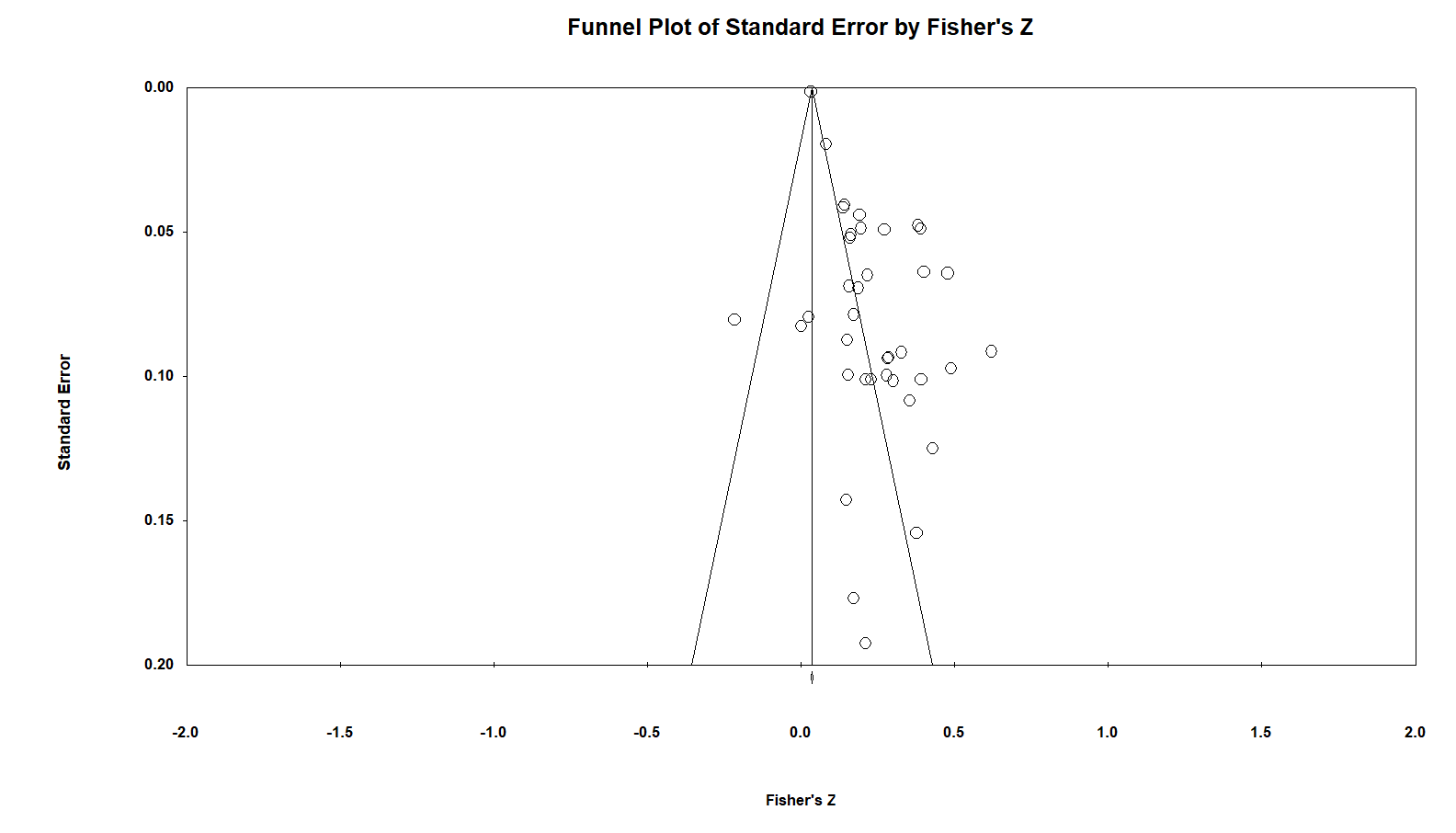 | 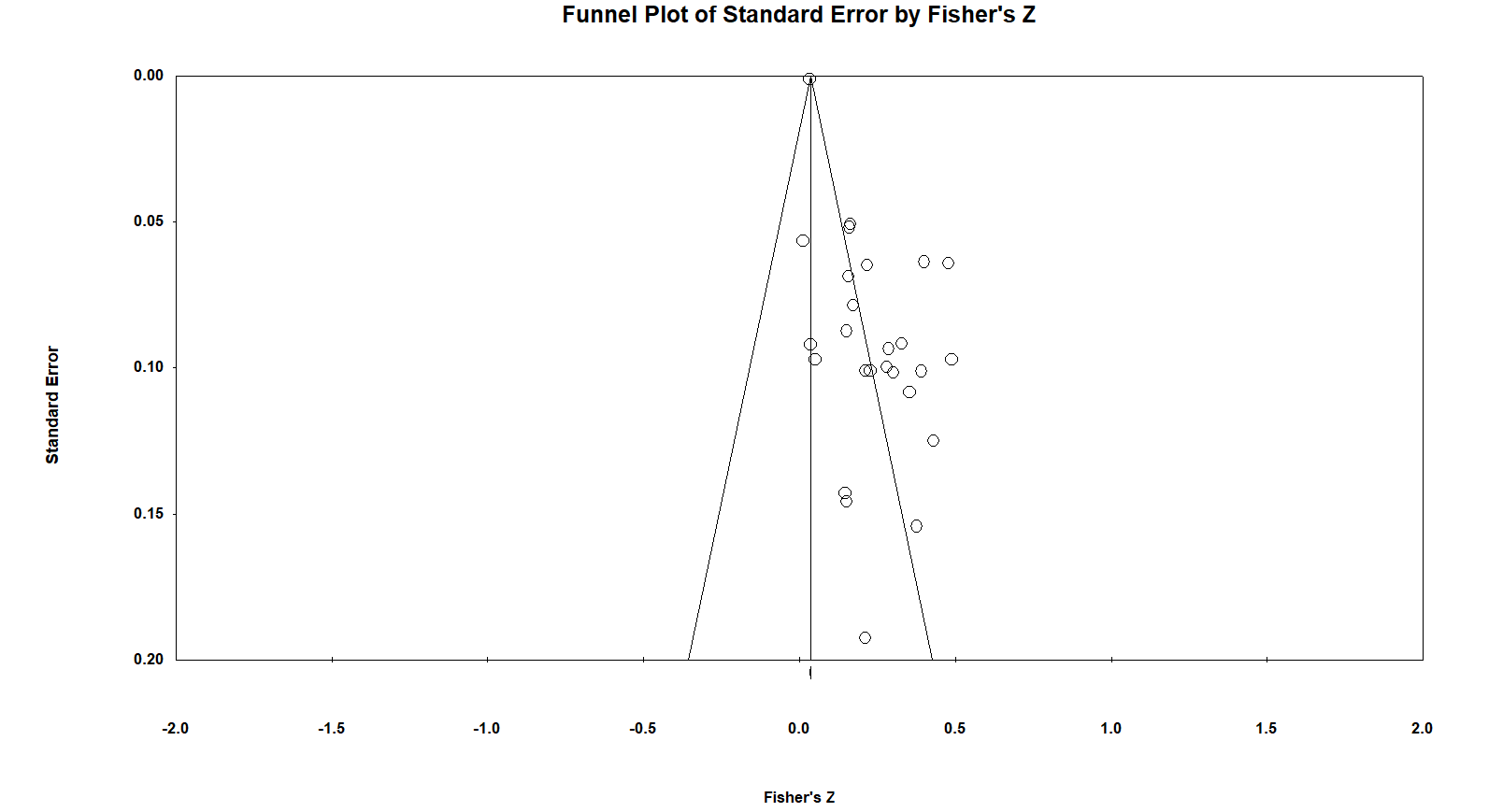 |
| **Speed** | **Attention** |
| 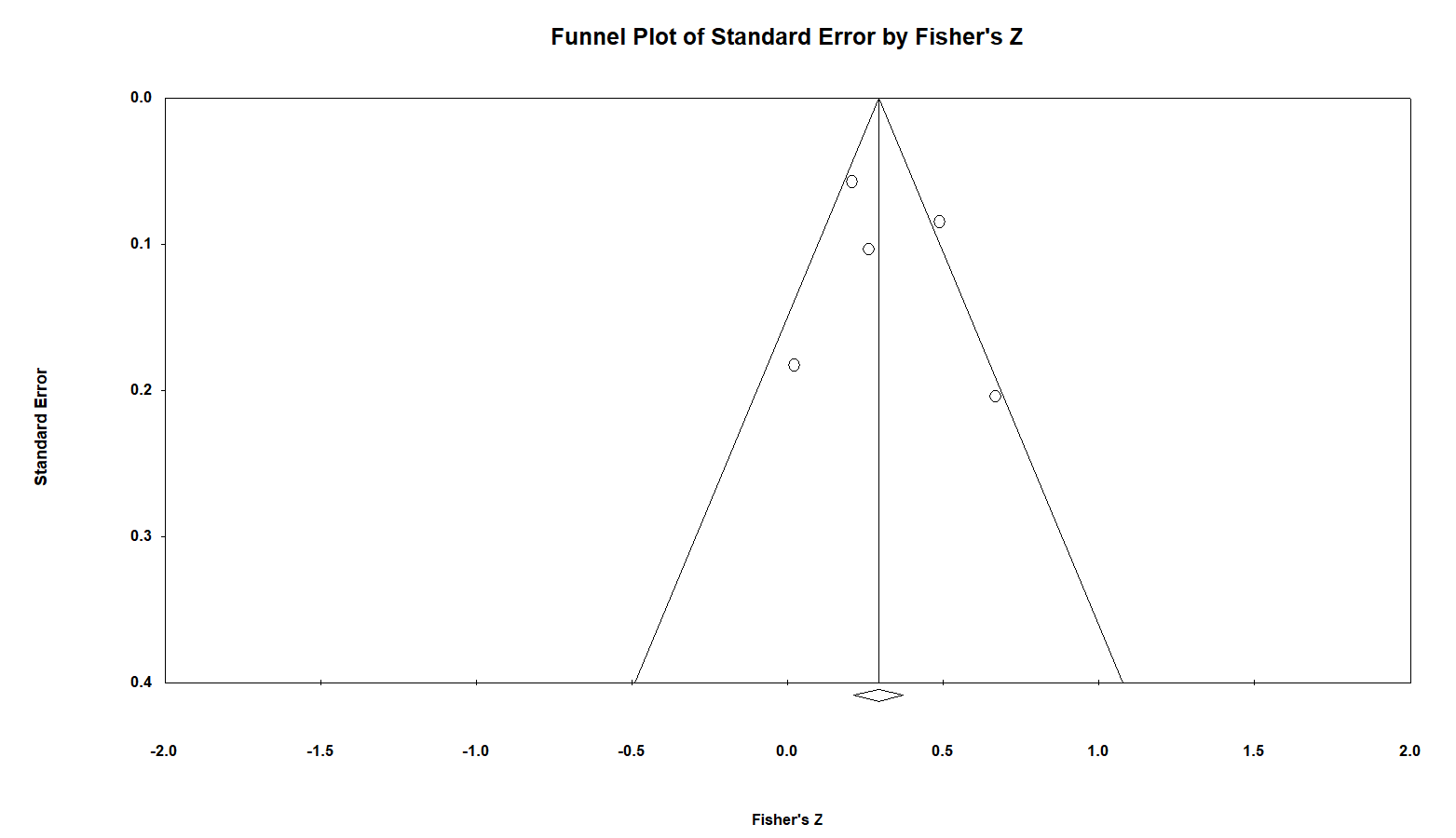 | 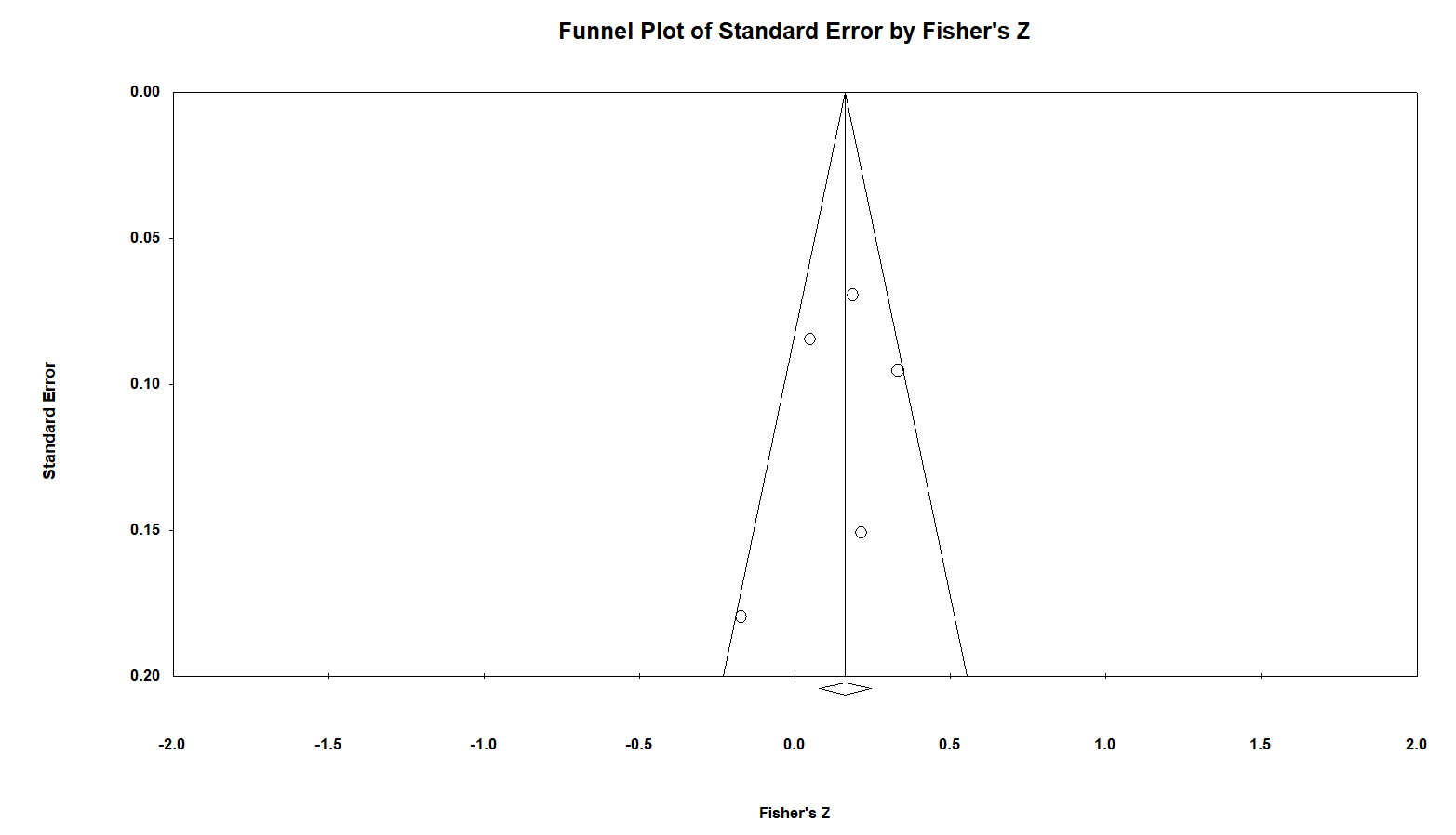 |
| **Visuospatial and Neglect** | **Language** |
| 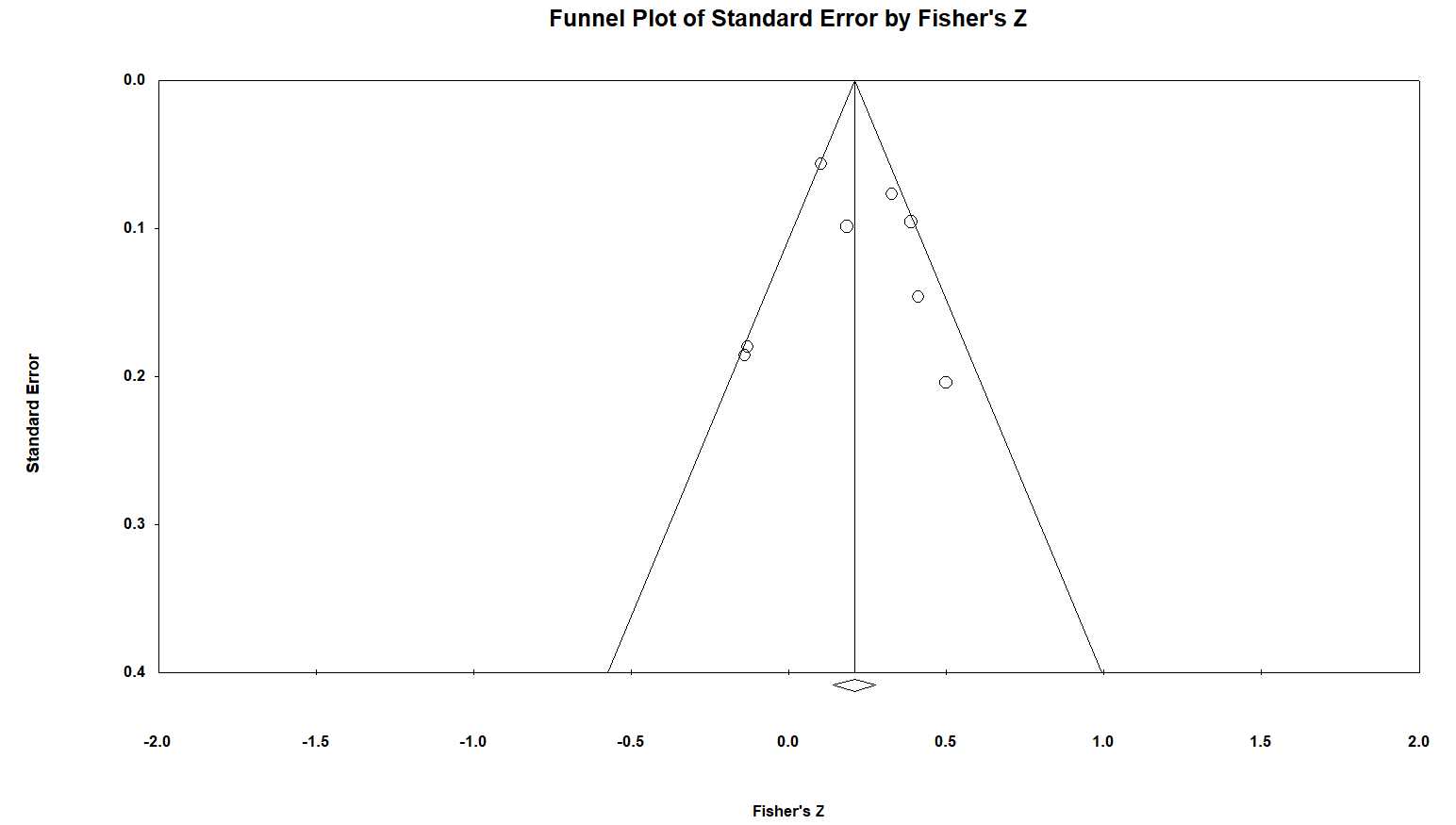 | 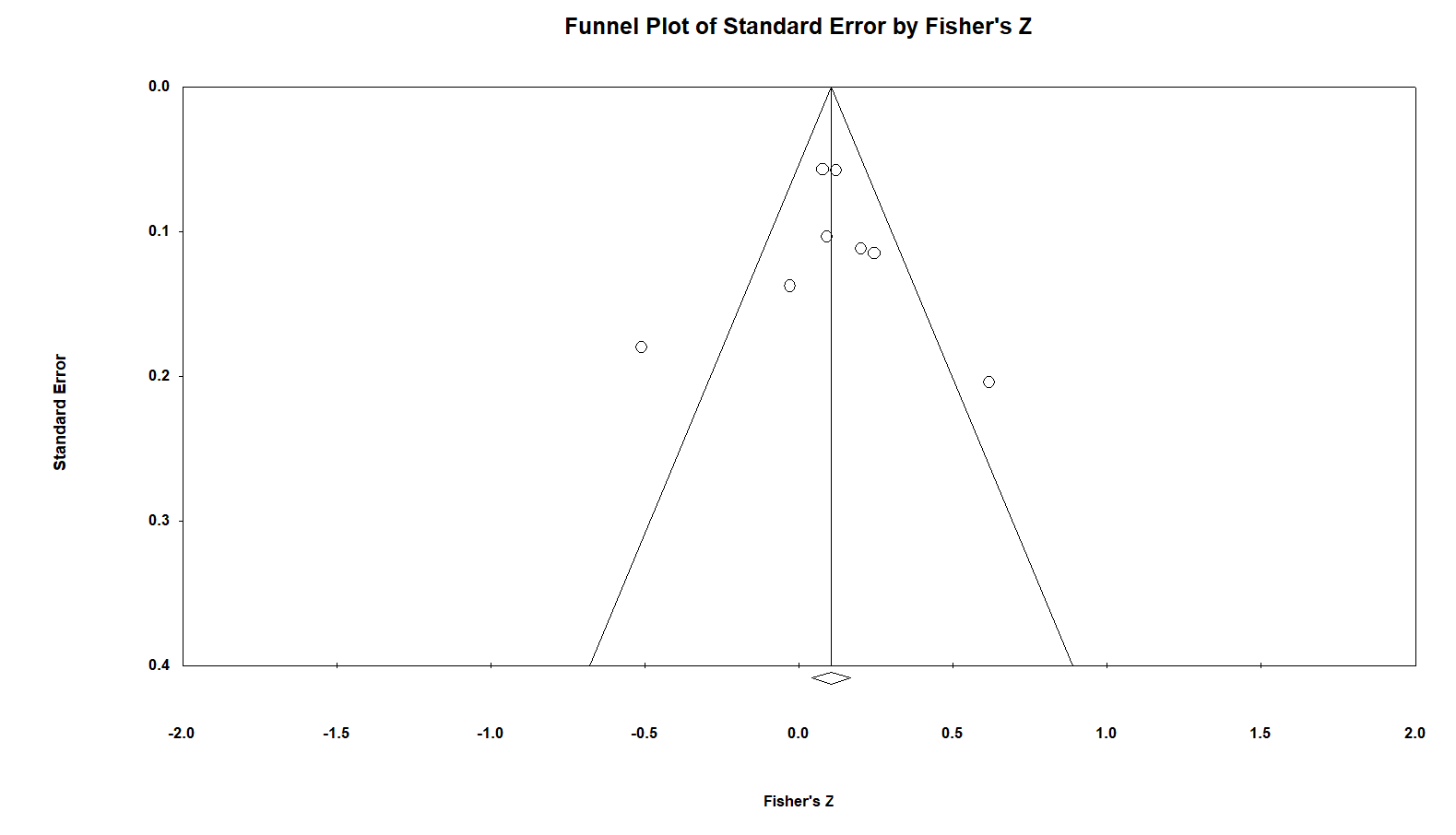 |
| **Memory** | **Executive** |
| 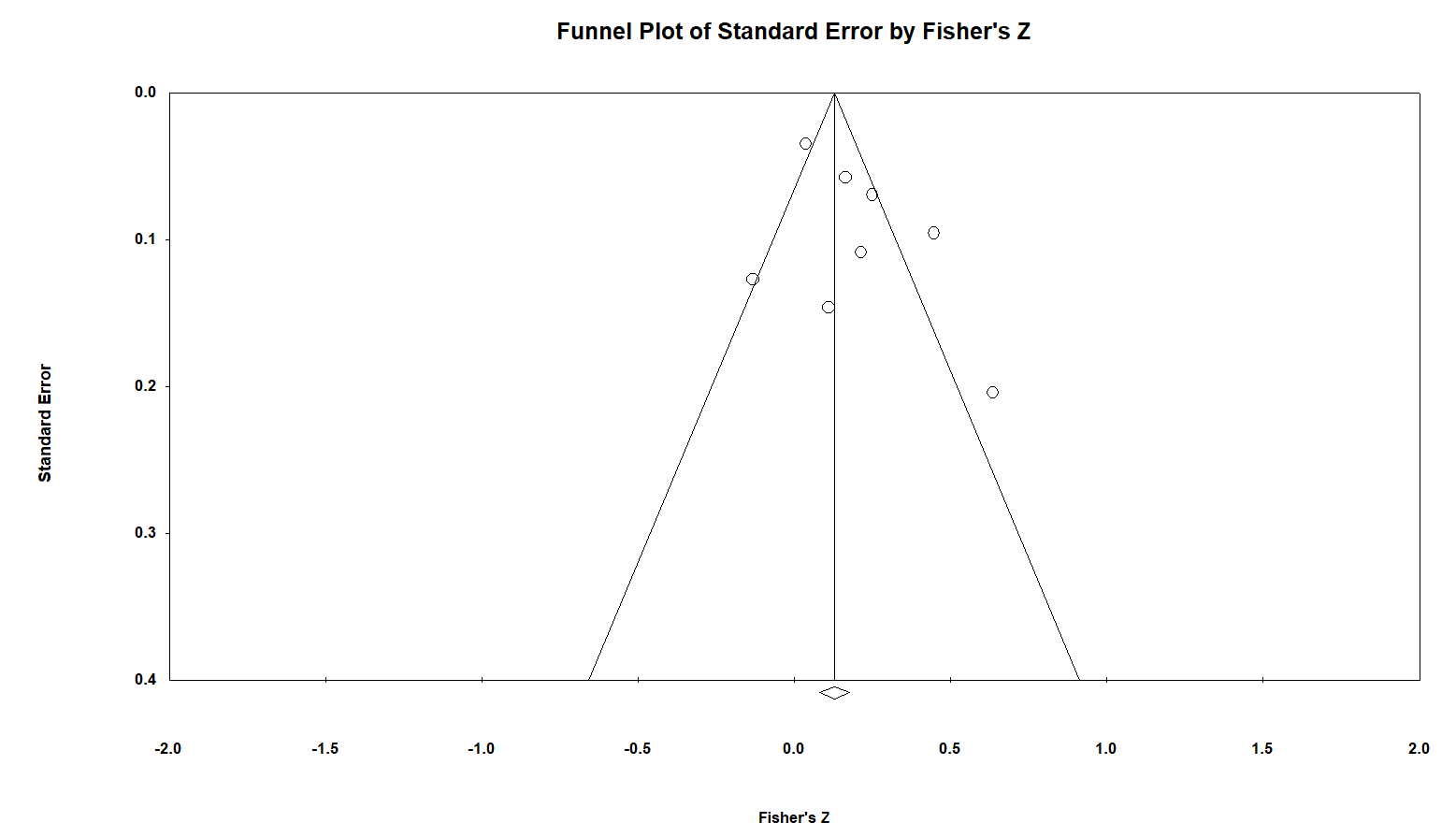 | 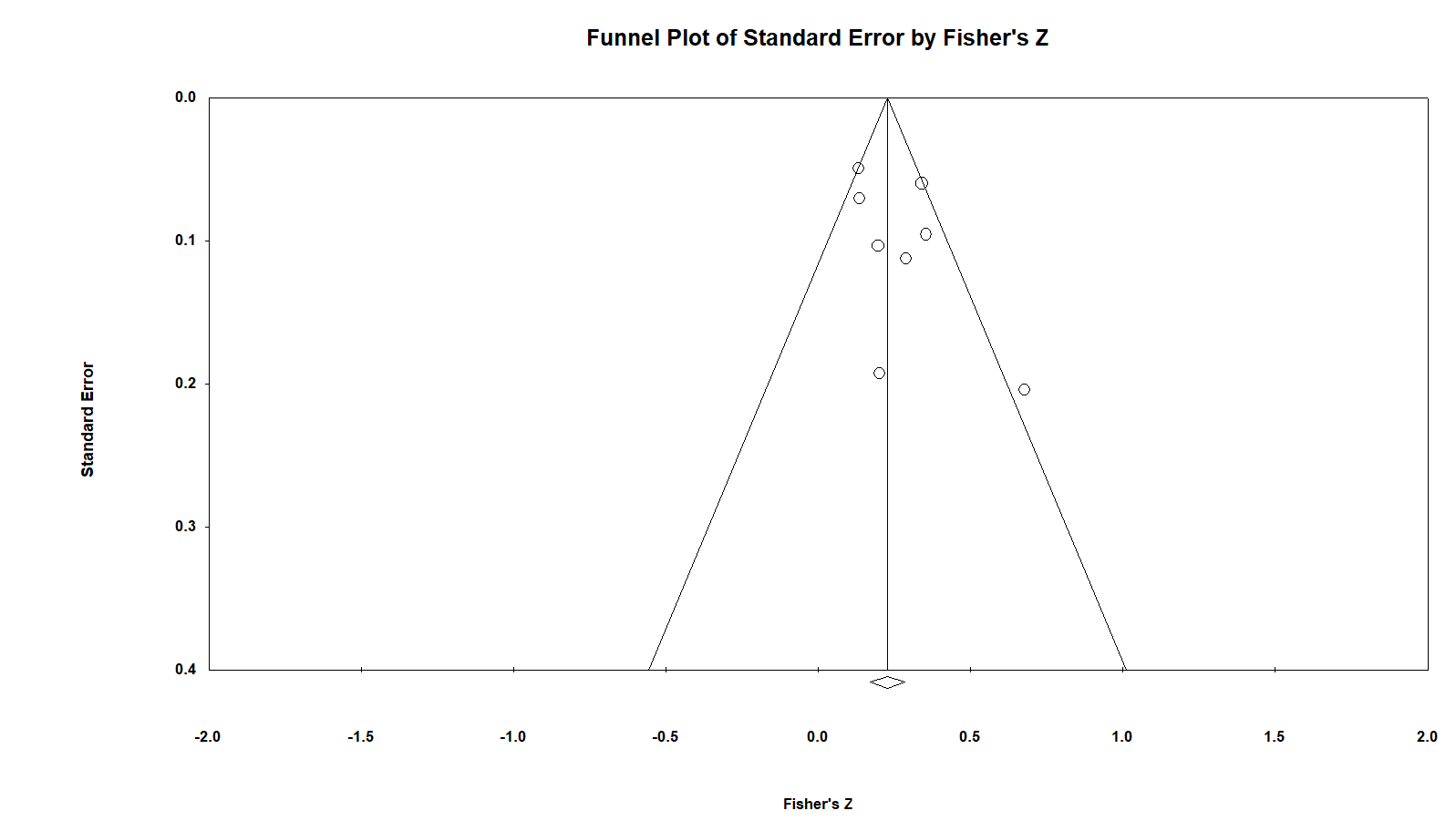 |

**Funnel Plots – Stroke Survivor Cognition and Caregiver Outcomes**

| **All Outcomes (QoL and Burden)** | **Burden** |
| --- | --- |
| 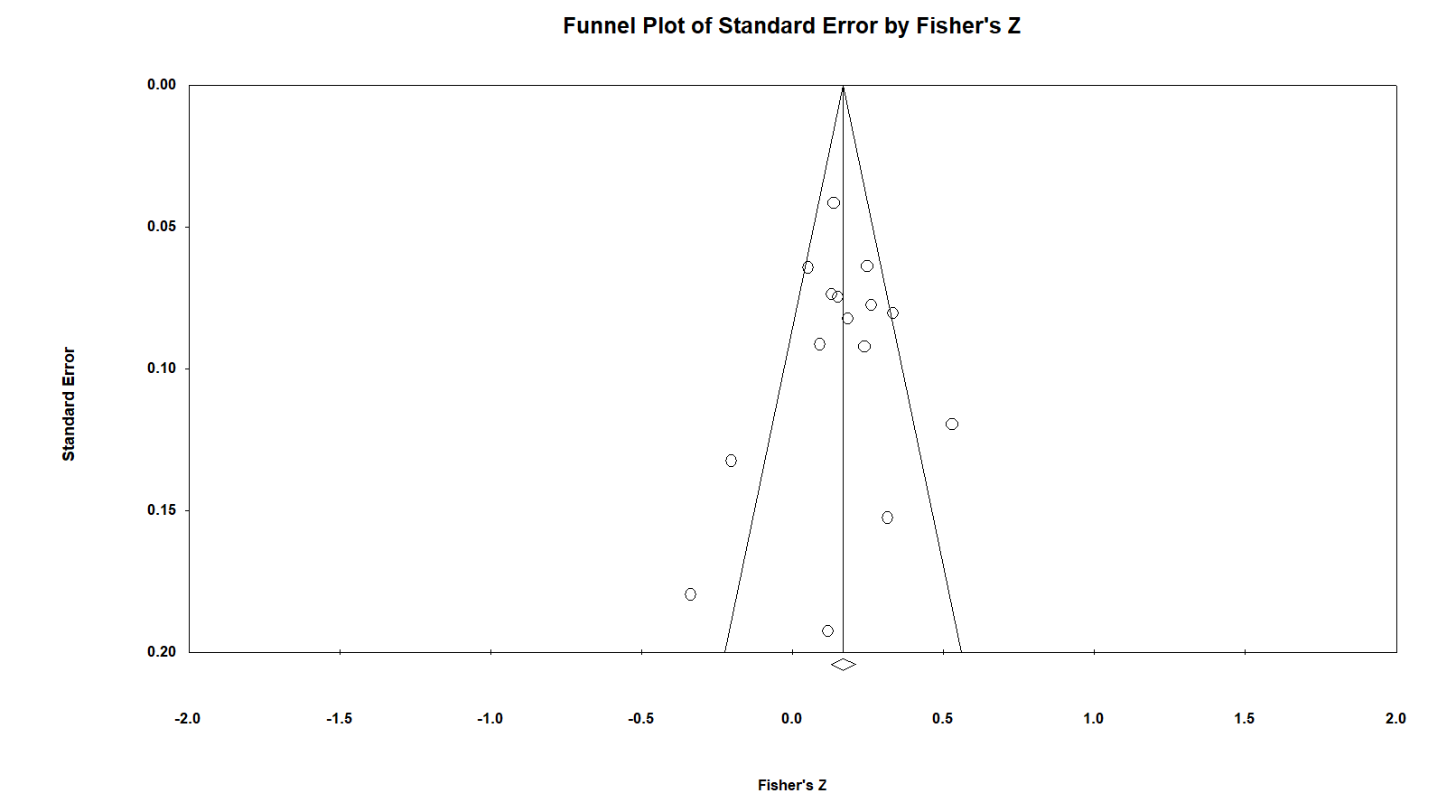 | 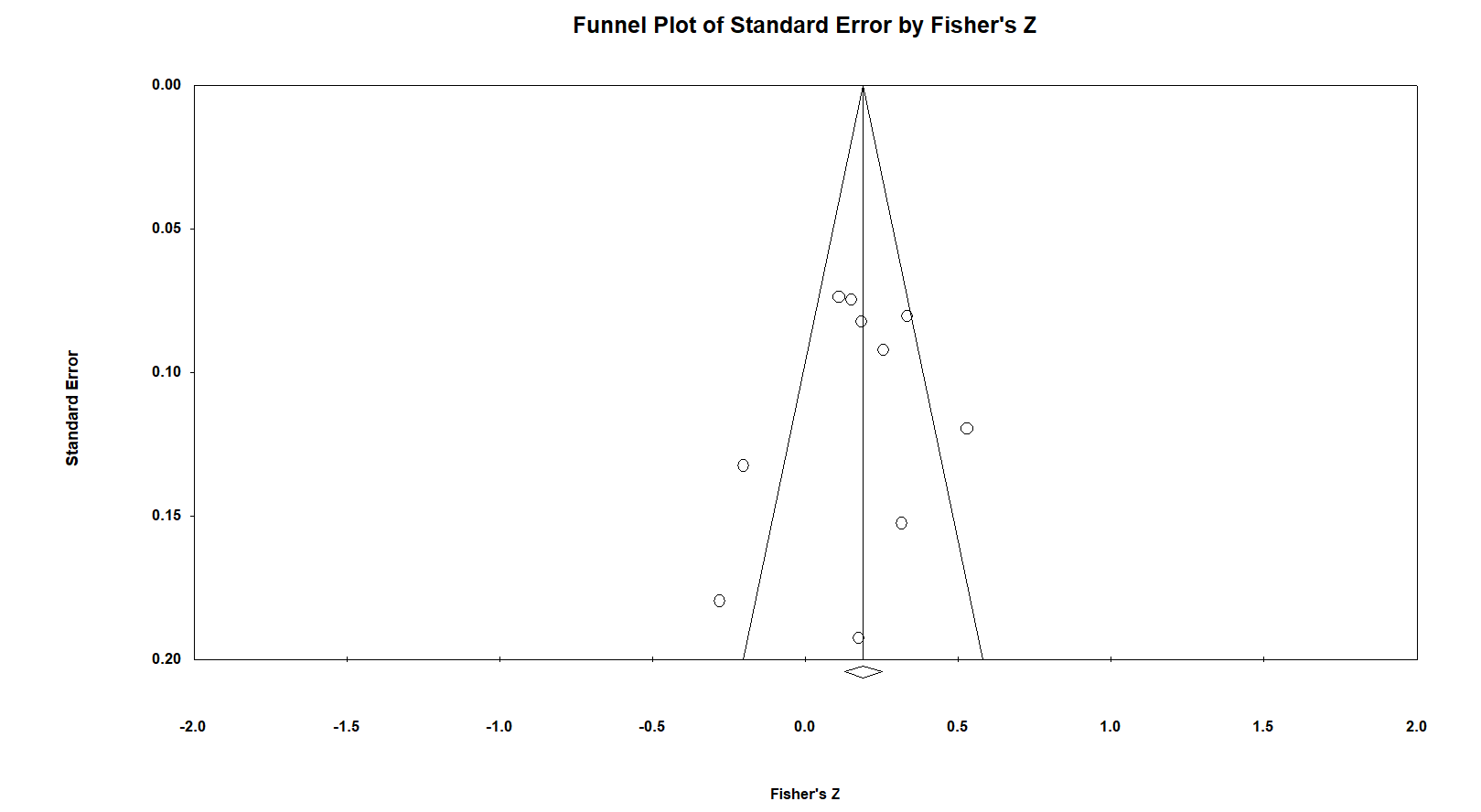 |
| **QoL** |  |
| 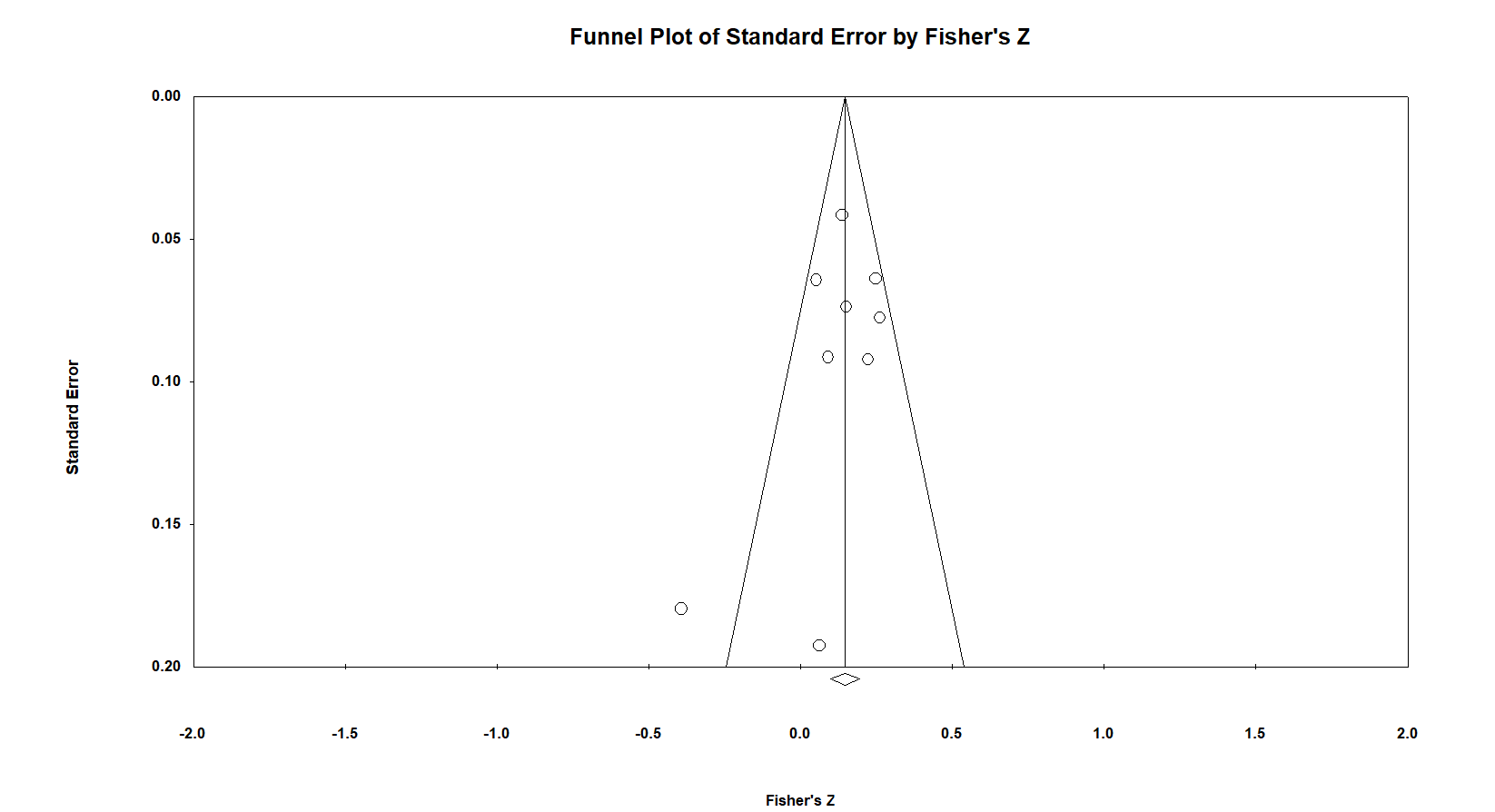 |  |
